# Resveratrol-based cinnamic ester hybrids: synthesis, characterization, and anti-inflammatory activity

**DOI:** 10.1080/14756366.2017.1381090

**Published:** 2017-10-26

**Authors:** Ban-Feng Ruan, Wei-Wei Ge, Hui-Jie Cheng, Hua-Jian Xu, Qing-Shan Li, Xin-Hua Liu

**Affiliations:** a School of Medical Engineering, Hefei University of Technology, Hefei, P. R. China;; b Anhui Province Key Laboratory of Major Autoimmune Diseases, Anhui Institute of Innovative Drugs, School of Pharmacy, Anhui Medical University, Hefei, P. R. China

**Keywords:** Resveratrol derivatives, cinnamic ester, anti-inflammatory, NF-κB signaling pathway, molecular docking

## Abstract

Twenty-three novel resveratrol-based cinnamic ester hybrids were designed and synthesized. All the compounds were evaluated for their anti-inflammatory activity using RAW264.7 cells. Among them, compound **D15** was found to be the most potent one in inhibiting NO production in LPS-stimulated RAW264.7 cells. The further study indicated that compound **D15** could suppress expression of proteins iNOS, COX-2, p-p65, and p-IκB LPS-induced. Immunofluorescence further revealed compound **D15** could reduce activation p65 in nuclei. All the results indicated that the anti-inflammatory activity of title compound may partly due to its inhibitory effect on the NF-κB signaling pathway.

## Introduction

Inflammation is a common and essential pathological process that the immune system uses as response to a large variety of stimuli such as injury or infection^1^. The ubiquitously expressed nuclear transcription factor-κB (NF-κB), plays a core role in the inflammatory response by regulating the expression of various genes encoding pro-inflammatory cytokines, adhesion molecules, chemokines, growth factors, and inducible enzymes such as cyclooxygenase-2 (COX-2) and inducible nitric oxide synthase (iNOS)^2^^,^^3^. A growing body of evidences indicate that the inflammatory process may cause tissue damage and a host of diseases such as cancer^4^^,^^5^. Nonsteroidal anti-inflammatory drugs (NSAIDs) have been considered as one of the most widely used medicines for alleviation of pain, fever as well as inflammations. However, the traditional NSAIDs drug is often accompanied by severe adverse effects, the main of which include gastrointestinal adverse effects^6^. Therefore, the development of novel therapeutic agentswith improved pharmaceutical profiles is now in urgent need.

**Figure 1.**
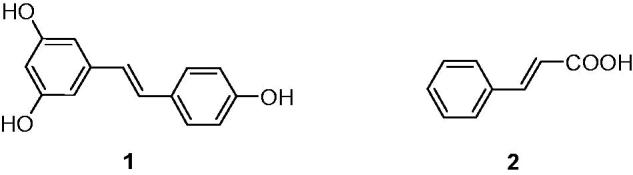
Structure of resveratrol (1) and cinnamic acid (2).

Natural products have long been deemed as lead templates for the design and discovery of novel anti-inflammatory agents^7^^,^^8^. Resveratrol (trans-3,5,4’-trihydroxylstilbene, **1**, Figure 1), a natural product with a stilbene structure, is without a doubt the most famous dietary polyphenol presents in medicinal plants^9^. In recent years, resveratrol has been extensively investigated as cardioprotective, anti-oxidative, anticancer, anti-aging agents^10–15^, and treatment of type II diabetes^16–18^. Resveratrol also plays an important anti-inflammatory role in human umbilical vascular endothelial cell^19^. More and more evidences have indicated that resveratrol is expected to be a new moiety for treatment of inflammation by reducing proinflammatory cytokines. It also can alleviate inflammation LPS-induced in Caco-2 and SW480 human colon cancer cell through inhibiting NF-κB pathway, protect LPS-induced extracellular lipoperoxidation^20–22^. Besides, resveratrol shows anti-inflammatory activity by inhibiting the TLR4/NF-κB/STAT signaling cascade^23^, modulating the cytokines-stimulated activation of SAPK/JNK pathway^24^, affecting MAPKs signaling cascades^25^. Furthermore, resveratrol has been reported to be endowed with the capacity of protecting the gastric mucosa against the side effect NSAID-induced^26^.

On the other hand, cinnamic acid (**2**, Figure 1) is a natural organic acid in plants with high safety and a variety of pharmacological activities, such as antioxidant, antimicrobial, anticancer, and anti-inflammatory activities^27^^,^^28^. Due to their common occurrence in plants and low toxicity, cinnamic acid derivatives have been evaluated as pharmacologically active compounds^29^. Among them, cinnamamide derivatives were identified as neuroprotective, anti-microbial, anti-nociceptive, and anti-inflammatory^28^^,^^30–32^. In our previous research, we have reported a series of novel resveratrol-cinnamamide hybrids with significant antitumor activity^33^. As a part of our continuous interest in search of active natural analogs with anti-inflammatory activity, herein, some new resveratrol-based cinnamic esters were synthesized and evaluated as anti-inflammatory. Molecular docking studies were consequently performed to identify the possible binding mode.

## Experimental part

### Chemistry

#### General

The ^1^H NMR and ^13 ^C NMR spectra were recorded on a VNMRS600 model spectrometer in DMSO solutions at room temperature with TMS as an internal standard. Chemical shifts (d) for ^1^H NMR and ^13 ^C NMR spectra were reported in parts per million to residual solvent protons. Melting points were measured on a Boetius micro melting point apparatus. EI-MS were obtained on a Mariner System 5304 mass spectrometer.

#### Synthesis of compounds D1–23

To a solution of compound **3** (1 mmol, 0.340 g), *N, N*'-dicyclohexylcarbodiimide (DCC) (1.2 mmol, 0.206 mg), 4-dimethylaminopyridine (DMAP) (0.1 mmol, 0.012 mg) in dichloromethane (10 ml) was added the proper aromatic substituted phenolic compound (1.1 mmol). The mixture was stirred for 2 h at room temperature. After completion of the reaction (TLC analysis), the mixture was diluted with water and extracted with dichloromethane. The combined organic layers were dried over anhydrous sodium sulfate, concentrated *in vacuo*, and purified by column chromatography on silica gel (PE:EtOAc = 12:1), to afford the corresponding pure products **D1**–**23**.

### Biological test

#### Cell culture

Mouse macrophage cell line RAW 264.7 was purchased from the Type Culture Collection of Chinese Academy of Sciences (Shanghai, China). Cells were cultured in DMEM medium supplemented with 10% FBS (Tianhang Biotechnology, Zhejiang, China), 100 U/mL penicillin-G and 100 g/ml streptomycin (Beyotime, Shanghai, China) at 37 °C in an atmosphere of 5% CO_2_.

#### Assay for NO production

NO production was quantified by nitrite accumulation in the culture medium using the Griess reaction. Briefly, RAW264.7 cells were pretreated with compounds for 4 h, and then stimulated with or without LPS (1 µg/mL) for 24 h. The isolated supernatants were mixed with an equal volume of Griess reagent (Beyotime Biotechnology, China). NaNO_2_ was used to generate a standard curve, and nitrite production was determined by measuring the optical density at 540 nm by a microplate reader (M1000, TECAN, Austria GmbH, Austria)^34^.

#### Cell viability assay

Cell cytotoxicity was evaluated by MTT assay as reported^35^. The medium was changed before the assay. Mouse macrophage cell line RAW 264.7 were seeded in 96-well plates, after serum starvation overnight, and cells were exposed to **D15** (6.25–50 µM) for 24 h. MTT dissolved in phosphate buffered saline (PBS) and was added to the culture medium to reach a final concentration of 0.5 mg/mL. After incubation at 37 °C for 4 h, the culture media containing MTT were removed, and then DMSO was added into each well and the absorbance at 570 nm was measured by a microplate reader (TECAN M1000, Austria GmbH, Austria).

#### Western blot

The RAW 264.7 cells were plated at a density of 5 × 10^5^ cells/well, which were treated with compound **D15** (10, 20, 40 µM) and Bay 11–7082 (0.3 µg/mL) for 12 h, and then stimulated by LPS (1 ng/mL) for 3 h (p65, IκB). Subsequently, cells were lysed with RIPA lysis buffer (Beyotime, Shanghai, China). Whole extracts were prepared, and the protein concentrations were determined using a BCA protein assay kit (Boster, Wuhan, China). Equal amounts of protein lysates (30 μg) were separated by SDS-PAGE (10%, 80 V for 30 min and then 120 V for 60 min). The proteins were transferred onto a PVDF membrane (Millipore Corp, Billerica, MA). Then the PVDF membranes will be incubated in TBS/Tween-20 containing 5% nonfat dry milk at 37 °C for 3 h. After blocking, the PVDF membranes were incubated with specific primary antibodies overnight at 4 °C. Rabbit monoclonal antibodies against NF-κB p65, NF-κB phospho-p65, NF-κB IκB, NF-κB phospho-IκB (Cell Signaling Technology) and mouse monoclonal anti-β-actin (ZSGB-BIO, Beijing, China) were used at 1:1000. Following incubation with primary antibodies, blots were washed three times in TBS/Tween-20 before incubation at 37 °C for about 1 h in goat anti-mouse or goat anti-rabbit horseradish peroxidase (Santa Cruz Biotechnology, Santa Cruz) conjugate antibody at 1:10 000 dilution in TBS/Tween-20 containing 5% nonfat dry milk. After extensive washing in TBS/Tween-20 for another three times, the membranes were detected by the enhanced chemiluminescence system. Proteins were visualized with ECL chemiluminescent kit (ECL-plus, Thermo Fisher Scientific, Waltham, MA). Autoradiographs were scanned using an Image-Pro Plus Imaging analysis software (Media Cybernetics, Rockville, MD)^35^.

#### Immunofluorescence assay

Cells were pretreated with compound **D15** and Bay 11–7082 (0.3 µg/mL) for 12 h before stimulation with 1 ng/ml LPS for 3 h. The cells were fixed with ice-acetone for 15 min, permeabilized with 0.3% TritonX-100 in PBS for 15 min, and then blocked with PBS (Boster, Wuhan, China) containing 5% bovine serum albumin (BSA, Sigma, St. Louis, MO) for 1 h. The cells were then incubated with the primary antibody against NF-κB p65 (1:500) overnight at 4 °C, followed by detection with a FITC-conjugated anti-rat IgG (Molecular Probes, Beijing, China) in the dark for 40 min at 37 °C. Nuclear staining was incubated with 4',6-diamidino-2-phenylindole, dilactate (DAPI; Invitrogen, Carlsbad, CA). Cells were washed and imaged using an inverted fluorescence microscope (Olympus, Tokyo, Japan)^35^.

### X-ray crystallography

The crystallographic data for compound **D2** were collected on a Bruker Smart 1000 CCD area detector diffractometer. Equipped with Mo Kα (λ = 0.71073 Å) radiation using ω-scan mode. Empirical absorption correction was applied to the data. The structures were solved by direct methods and refined by full-matrix least-squares methods on *F*^2^. All non-hydrogen atoms were located from the trial structure and then refined anisotropically. All hydrogen atoms were generated in idealized positions and were assigned fixed isotropic thermal parameters at 1.2 times the equivalent isotropic U of the atoms to which they are attached and allowed to ride on their respective parent atoms. The contributions of these hydrogen atoms were included in the structure-factors calculations.

### Molecular docking

Molecular docking of compound **D15** into the three-dimensional COX-2 complex structure (PDB code: 1cx2) was carried out by using the Discovery Studio 2017 (D S 2017, Accelrys, Inc., San Diego, CA) software. The three-dimensional structures of the compounds were constructed by using Chem. 3 D ultra 12.0 software (Chemical Structure Drawing Standard, Cambridge Soft Corporation, Cambridge, MA), then they were energetically minimized by using MMFF94 with 5000 iterations and minimum RMS gradient of 0.10. The crystal structures of protein complex complexity were retrieved from the RCSB Protein Data Bank (http://www.rcsb.org/pdb/home/home.do) and prepared by DS 2017 with all bound waters and ligands eliminated from the protein and the polar hydrogen added to the protein. The molecular docking procedure was performed by using CDOCKER protocol for receptor-ligand interactions section of DS 2017.

## Results and discussion

### Chemistry

**Scheme 1.**
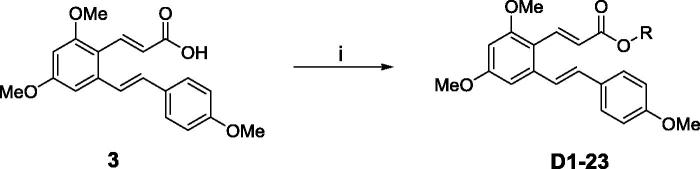
Reagents and conditions: (i) DCC, DMAP, DCM, substituted aromatic phenolic compounds, room temperature, 2 h.

The synthetic route was depicted in Scheme 1. Compound **3**, which was prepared as previously described^33^, was reacted with substituted aromatic phenolic compounds, in presence of N, N’-dicyclohexylcarbodiimide (DCC) and 4-dimethylaminopyridine (DMAP) to give the title compounds, see Table 1.

**Table 1.** Chemical structures of the title compounds.


Compd.	R	Compd.	R
**D1**		**D13**	
**D2**		**D14**	
**D3**		**D15**	
**D4**		**D16**	
**D5**		**D17**	
**D6**		**D18**	
**D7**		**D19**	
**D8**		**D20**	
**D9**		**D21**	
**D10**		**D22**	
**D11**		**D23**	
**D12**			

The purity and spectra of all compounds were detailed in Supporting Information. All the obtained compounds gave satisfactory elementary analysis and spectroscopic data. ^1^H NMR,^13 ^C NMR and ESI MS spectra were consistent with the structures. Furthermore, the structure of compound **D2** was also confirmed by single crystal X-ray diffraction analysis.

**Figure 2.**
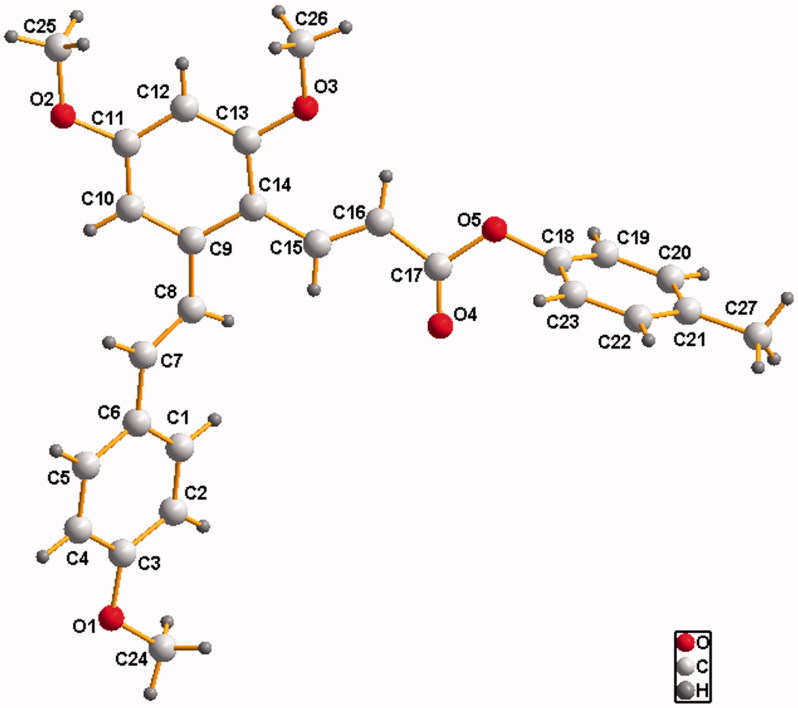
Crystal structure of compound **D2**.

Compound **D2** was crystallized in the monoclinic space group *P*_1_c1. The crystal data and refinement data are listed in Table 2. Selected bond lengths and angles are given in Table 3. All bond lengths are within normal ranges. As shown in Figure 2, C7-C8 and C15-C16 are both in trans form and the bond lengths of 1.327(7) and 1.321(7) Å conform to the value for double C-C bond, respectively. Similarly, the bond length of C17-O4 (1.179(7) Å) conforms to the value for C=O bond, which is a little shorter than the length of C=O (1.225(5) Å) that we observed previously in a resveratrol amide derivative (E)-3–(2,4-dimethoxy-6-((E)-4-methoxystyryl)phenyl)-1–(3,5-dimethylpiperidin-1-yl)prop-2-en-1-one^33^. The dihedral angle between the phenyl rings C9-C10-C11-C12-C13-C14 and C18-C19-C20-C21-C22-C23-C24 is 87.8 (2)° while the dihedral angle between the phenyl rings C1-C2-C3-C4-C5-C6 and C18-C19-C20-C21-C22-C23-C24 is 41.4 (2)°.

**Table 2.** Crystallographic data and structure refinements for compound **D2.**

Compound	**D2**
Empirical formula	C_27_H_26_O_5_
Molecular weight	430.48
Crystal size (mm^3^)	0.24 × 0.18 × 0.04
Temperature(K)	293.15
Crystal system	Monoclinic
Space group	P1c1
a (Å)	16.3309(7)
b (Å)	7.3529(5)
c (Å)	9.5704(4)
α (°)	90.00
β (°)	100.726(4)
γ (°)	90.00
V (Å^3^)	1129.13(10)
Z	2
Dc (g cm^−3^)	1.266
μ (mm^−1^)	0.087
F (000)	456.0
θ rang (deg)	3.52-26.01
Reflections collected	3782 (*R*_int_ = 0.0395)
Indep. reflns	2565
Refns obs. [I > 2σ(I)]	1725
Data/restr./paras	2565/2/293
Goodness-of-fit on F2	1.098
R1, wR2(all data)	R1 = 0.0894, wR2 = 0.1515
R1, wR2 [I > 2σ(I)]	R1 = 0.0584, wR2 = 0.1238
Larg.peak/hole(e. Å)	0.165/-0.218
CCDC NO.	1549457

**Table 3.** Selected bond lengths (Å) and angles (°) for compound **D2.**

Bond lengths			
C9-C8	1.476(7)	C8-C7	1.327(7)
C7-C6	1.466(8)	C14-C15	1.461(6)
C15-C16	1.321(7)	C16-C17	1.451(7)
C3-O1	1.373(8)	C11-O2	1.373(5)
C13-O3	1.362(8)	C17-O4	1.179(4)
C18-O5	1.402(6)	C17-O5	1.359(6)
C26-O3	1.425(7)	C25-O2	1.445(7)
C24-O1	1.432(8)		
Bond angles			
C14-C9-C8	121.7(4)	C10-C9-C8	118.0(4)
C9-C8-C7	124.5(5)	C8-C7-C6	128.3(5)
C7-C6-C1	124.0(5)	C7-C6-C5	119.9(5)
C13-C14-C15	123.1(4)	C14-C15-C16	129.8(5)
C9-C14-C15	120.2(4)	C15-C16-C17	123.0(4)
C16-C17-O4	128.8(5)	O4-C17-O5	121.9(5)
C17-O5-C18	120.9(4)	O5-C18-C19	118.0(5)
O5-C18-C23	120.7(6)	C16-C17-O5	109.3(4)

### Biological evaluation

#### Inhibition of NO production LPS-stimulated RAW264.7 cells

**Figure 3.**
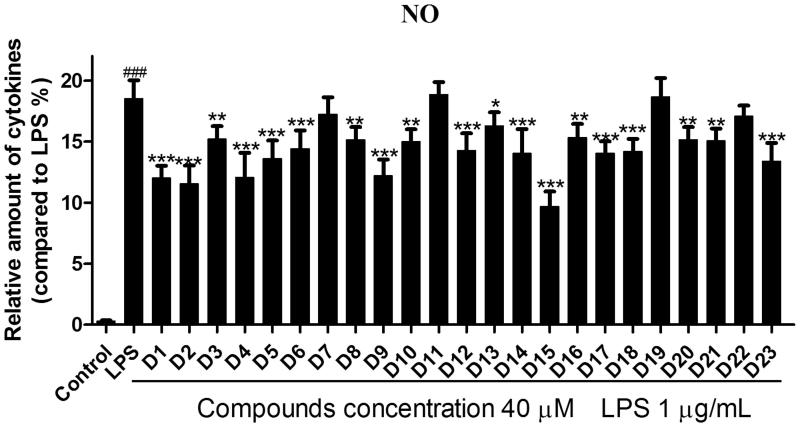
Effects of compounds **D1–D23** on production of NO by RAW264.7 cell RAW264.7 cells were pretreated with **D1–D23** (40 μM) for 4 h, and then stimulated with or without LPS (1 μg/mL) for 24 h. NO production was measured using nitrite and nitrate assay. ###*p* < .001 compared with unstimulated cells, *p < .05, **p < .01 and ***p < .001 compared with LPS-stimulated cells; Data were from at least three independent experiments, each performed in duplicate.

NO is a key pro-inflammatory mediator and excessive production of NO was proved to be associated with the pathogenesis of inflammation diseases^34^. It is generally accepted that NO inhibitors may offer potential opportunity to identify new therapeutic method for the inflammatory diseases^36^. To determine the potential anti-inflammation activity of these hybrids, LPS-induced RAW 264.7 cells were used as inflammatory cell model to evaluate the effects of compounds **D1–23** against release of NO. As shown in Figure 3, it could be seen that most compounds reflected certain effects at 40 µM. Compound **D1** exhibited relatively good inhibitory activity. In general, the introduction of methyl group to the phenyl ring (**D1**) could lead to an obvious decline in potency (**D3**–**D8**). However, compound **D2** is an exception. The introduction ofpara-CH_3_ group to **D1** exhibited better inhibitory activity. Moreover, there was no positive influence on inhibitory activity appending electron-with drawing group like chlorine and iodine at the benzene ring of compound **D1**. The 4-chloro substituted analog compound **D9** exhibited almost the same inhibitory activity as compound **D1**. However, he replacement of the chlorine atom with an iodine one caused significant decrease of activity. What is more, there was a great decrease in inhibitory activity of compound **D11** when introduced 3′,5′-dimethyl groups onto compound **D9**. Compound **D15** showed the most potent anti-inflammatory activity. Modifications of **D15** with naphthalene ring and biphenyl group generated **D16–23** leaded to obviously weaker inhibitory activity than compound **D15**. Comparison of the activity of compounds **D19**, **D20** and **D21**, it could be seen that the position of the substituent grouphad an obvious effect on activity. These preliminary SAR results could be very helpful for our further SAR study. To get a better insight into the mechanism of LPS-induced NO release inhibition, the most potent compound **D15** was subjected to the further study.

#### Compound D15 inhibited LPS-induced inflammatory mediators

**Figure 4.**
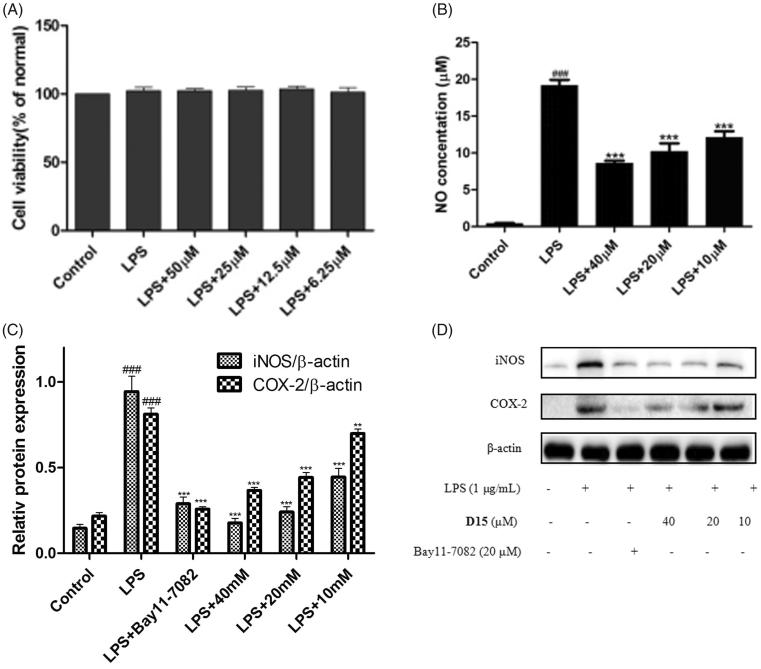
Compound **D15** inhibited LPS-induced inflammatory response in RAW 264.7 cells. Cells were treated with compound **D15** (10, 20, 40 µM) for 12 h, and then stimulated by LPS (1 µg/ml) for 3 h. Cell viability was evaluated using the MTT assay. NO production was measured using nitrite and nitrate assay. iNOS and COX-2 expression were detected by Western blot analysis. (A) Cell viability assay; (B) Quantitative analysis of NO production. (C) Quantitative analysis of iNOS and COX-2 expression, β-actin was used as loading control. ###*p <* .001 compared with unstimulated cells, **p < .01 and ***p < .001 compared with LPS-stimulated cells. Data were from at least three independent experiments, each performed in duplicate.

To investigate the safety of the selected compounds, the potential cytotoxicity of compound **D15** against the RAW264.7 cells was evaluated using MTT assay. As depicted in Figure 4(A), compound **D15** did not affect the cell viability from 6.25 to 50 μM, indicating that compound **D15** was non-cytotoxic at the above concentrations. Therefore, compound **D15** was used for the subsequent study from 6.25 to 50 μM.

Inflammation-related diseases are closely related to the expressions of iNOS and COX-2. Thus, the inhibitory effects of compound **D15** on LPS-mediated expression of iNOS and COX-2 were analyzed by Western blot. As shown in Figure 4(B,C), it could be seen that the LPS (1 µg/mL) stimulation significantly induced generation of NO production as well as the expressions of iNOS and COX-2. The results preliminary demonstrated that title compound could significantly inhibited LPS-induced expressions of iNOS and COX-2 in RAW264.7 cells.

#### Compound D15 inhibited LPS-induced NF-κB activation

**Figure 5.**
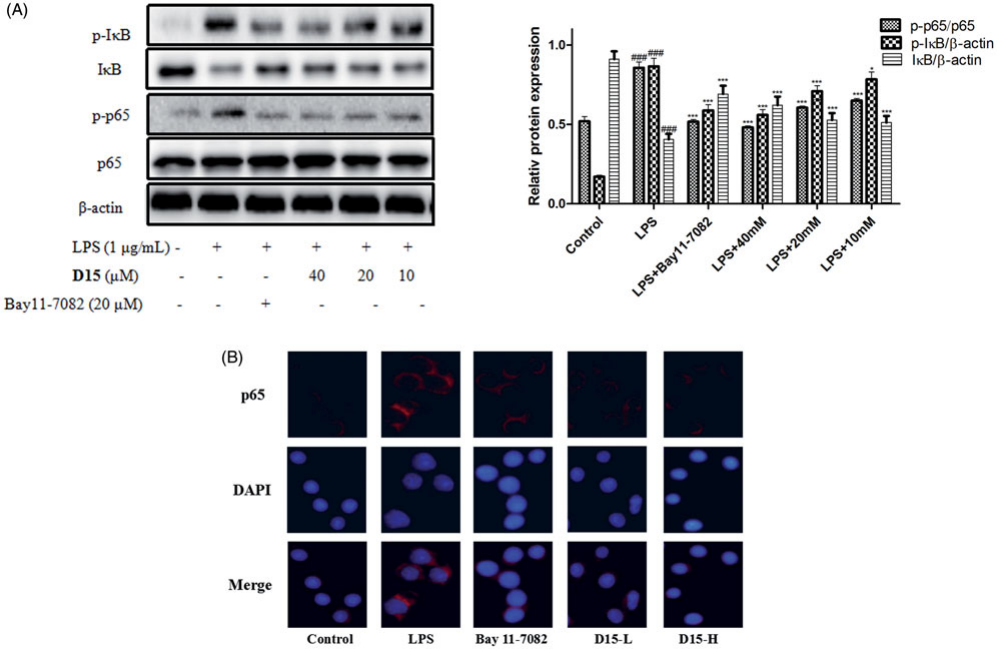
Compound **D15** suppressed LPS-induced activation of NF-κB signaling pathway in RAW 264.7 cells. After pretreatment with **D15** (10 ∼ 40 µM), RAW 264.7 cells were stimulated with LPS (1 µg/mL) for 30 min. The total and phosphorylation levels of NF-κB were detected by Western blot. (A) Quantitative analysis of p-IκB and p-p65, total IκB and p65 were used as loading control, respectively. (B) Immunofluorescence analysis of compound **D15**. ###*p* < .001 compared with unstimulated cells, *p < .05 and ***p < .001 compared with LPS-stimulated cells. Data were from at least three independent experiments, each performed in duplicate.

The NF-κB transcription factor family is a pleiotropic regulator of many cellular signaling pathways, providing a mechanism for the cells in response to a wide variety of stimuli to inflammation, which can activate the NF-κB signaling pathway^37^. Subsequently, NF-κB will be phosphorylated and the activated NF-κB will translocate from cytoplasm to nucleus promoting transcription of various inflammatory marker genes, including cytokines, chemokines, iNOS, and COX-2^35^. In order to understand the effect of title compound **D15** on NF-κB signaling LPS-induced, the relative levels of proteins p-IκB, IκB, p-p65 and p65 were examined by western blot. As shown in Figure 5(A), LPS markedly upregulated the expressions of p-p65 and p-IκB compared to normal group. However, compound **D15** concentration-dependently inhibited the expressions of above mentioned proteins LPS-induced, preliminary indicating that compound **D15**couldinhibit the activation of NF-κB. Consistently with the inhibitory effects of compound **D15** on the expressions of proteins p-p65 and p-IκB LPS-induced, immunofluorescence staining further confirmed that LPS stimulated cells showed a clear and positive labeling for the activation p65 in nuclei. Compared to bay 11–7082, compound **D15** could reduce this effect (Figure 5(B)).

#### Molecular docking

Molecular docking is an application wherein molecular modeling techniques are used to predict how a protein (enzyme) interacts with small molecules (ligands). In order to get more insight into anti-inflammatory mechanism of compound **D15** and obtain more SAR clues, molecular docking studies were performed using the reported COX-2 inhibitor complex structure (PDB code: 1cx2) which obtained from the RCSB protein data bank (http://www.pdb.org)^38^. The molecular docking procedure was performed by DS 2017 as described previously (Discovery Studio 2017, Accelrys, Inc., San Diego, CA)^39^^,^^40^.

**Figure 6.**
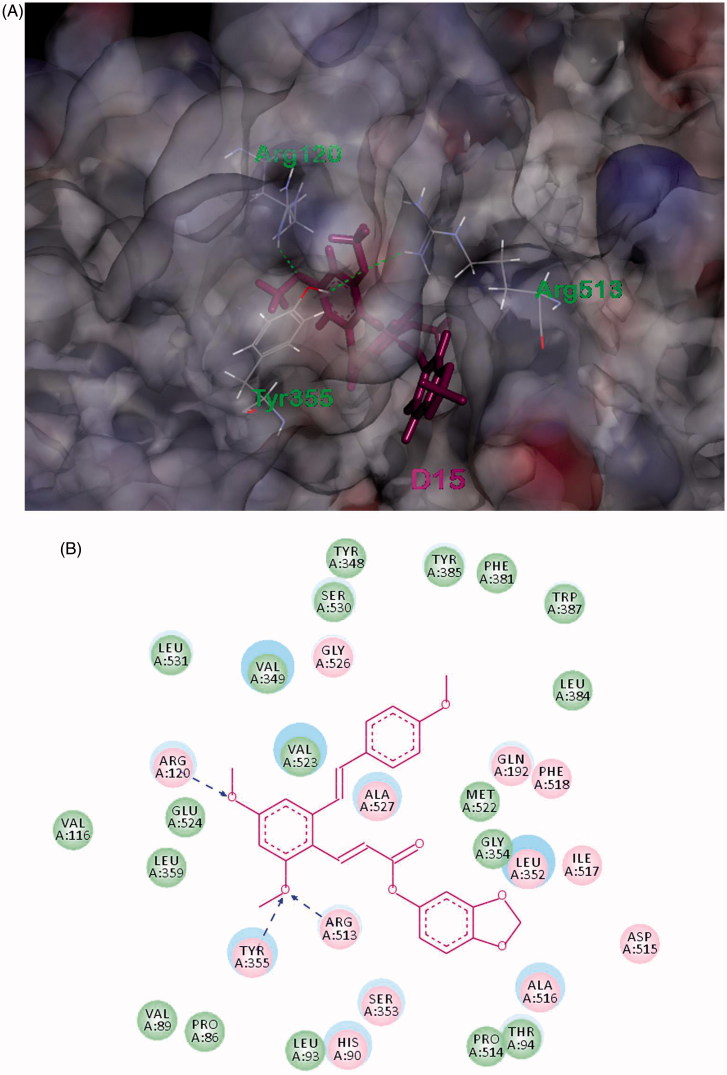
(A) Binding model of **D15** (purple) in the active site of COX-2. The H-bond is displayed as blue dashed line. (B) 2Dprojection drawing of **D15** docked into COX-2 active site.

The binding mode of compound **D15** within COX-2 was depicted in Figure 6. Visual inspection of the pose of **D15** into the COX-2-binding site revealed that it has suitable shape complementarity with the binding pocket (Figure 6(A)), thus showed favorable binding affinity (-CDOCKER_INTERACTION_ENERGY = -43.35 kcal/mol) to the receptor via variety of interactions. The model suggests that extensive hydrophobic interactions are formed between **D15** and the binding pocket of COX-2. Furthermore, its twomethoxyl groups in A-ring of resveratrol moiety formed three H-bond interactions with ARG120 (angle N-H···O = 144.0°, distance = 1.86 Å), TYR355 (angle N-H···O = 131.8°, distance = 2.05 Å), ARG513 (angle N-H···O = 143.8°, length = 2.15 Å), respectively, which were simultaneously contributed to the combination. Combining with the results of bioassays above, it was found that compound **D15** might be a potential inhibitor of the COX-2 through above synergic effect, which provided more insight into its anti-inflammatory mechanism and SAR clues for further optimization of resveratrol-based anti-inflammatory agents.

## Conclusions

In summary, a novel series of resveratrol-based cinnamic ester hybrids were designed, synthesized and characterized. Their anti-inflammatory activities were evaluated in a LPS-induced RAW264.7 cell model. Among the synthesized compounds, compound **D15** was found to be the most potent one to suppress NO production in LPS-induced RAW264.7 cells. Western blot experiments indicated that compound **D15** inhibited LPS-induced protein expression. Furthermore, immunofluorescence revealed that compound could D15 lightly reduce activation p65 in nuclei. All the results indicate that the anti-inflammatory role of compound D15 may partly due to its inhibitory effect on the NF-κB signaling pathway LPS-induced RAW 264.7 cells.

## Supplementary Material

IENZ_1381090_Supplementary_Material.pdf
