# Supplementary material for: Resveratrol-based cinnamic ester hybrids: synthesis, characterization, and anti-inflammatory activity
Source: J Enzyme Inhib Med Chem. 2017 Oct 26;32(1):1282–90. doi: 10.1080/14756366.2017.1381090 (PMC6009859; doi:10.1080/14756366.2017.1381090)
Supplement: IENZ_1381090_Supplementary_Material.pdf [file IENZ_A_1381090_SM1791.pdf]

Supporting Information

1. Chemistry

1.1. General

The ^{1}H NMR and ^{13}C NMR spectra were recorded on a VNMRS600 model spectrometer in DMSO solutions at room temperature with TMS as an internal standard. Chemical shifts (d) for ^{1}H NMR and ^{13}C NMR spectra were reported in parts per million to residual solvent protons. Melting points were measured on a Boetius micro melting point apparatus. EI-MS were obtained on a Mariner System 5304 mass spectrometer.

1.1.1. phenyl (E)-3-(2,4-dimethoxy-6-((E)-4-methoxystyryl) phenyl) acrylate ***(D1)***

Yellow solid, yield 82%, Mp: 95-97°C. ^{1}H NMR (600 MHz, $CDCl_3$): δ 8.21 (d, J = 16.0 Hz, 1H), 7.47 (d, J = 8.5 Hz, 2H), 7.39 (t, J = 7.9 Hz, 2H), 7.32 (d, J =16.0 Hz, 1H), 7.23 (t, J = 7.4 Hz, 1H), 7.18 (d, J = 8.4 Hz, 2H), 6.94 (d, J = 16.0 Hz, 1H), 6.90 (d, J = 8.6 Hz, 2H), 6.71 (d, J = 16.0 Hz, 2H), 6.45 (s, 1H), 3.91 (s, 3H), 3.90 (s, 3H), 3.83 (s, 3H). ^{13}C NMR (151 MHz, $CDCl_3$): δ 166.60, 161.70, 160.80, 159.67, 151.10, 141.79, 140.39, 132.26, 129.78, 129.29, 128.09, 125.47, 124.85, 121.76, 119.44, 114.98, 114.21, 103.66, 97.63, 55.65, 55.46, 55.33. MS (EI): 417.2 ($C_{26}H_{24}O_5$, $[M+H]^+$). Anal. Calcd for $C_{26}H_{24}O_5$: C, 74.98; H, 5.81; O, 19.21%. Found: C, 75.00; H, 5.82; O, 19.18%.

1.1.2. p-tolyl (E)-3-(2,4-dimethoxy-6-((E)-4-methoxystyryl) phenyl) acrylate ***(D2)***

Yellow solid, yield 80%, Mp: 78-81 °C. ^{1}H NMR (600 MHz, $CDCl_3$): δ 8.20 (d, J = 16.0 Hz, 1H), 7.47 (d, J = 8.6 Hz, 2H), 7.32 (d, J = 16.0 Hz, 1H), 7.19 (d, J = 8.1 Hz, 2H), 7.06 (d, J = 8.3 Hz, 2H), 6.94 (d, J = 16.0 Hz, 1H), 6.90 (d, J = 8.6 Hz, 2H), 6.73 (d, J = 2.0 Hz, 1H), 6.71 (d, J = 16.0 Hz, 1H), 6.45 (s, 1H), 3.91 (s, 3H), 3.90 (s, 3H), 3.83 (s, 3H), 2.36(s, 3H). ^{13}C NMR(151MHz, $CDCl_3$): δ 166.77, 161.63, 160.75, 159.64, 148.82, 141.70, 140.20, 135.03, 132.18, 129.79, 128.07, 124.86, 121.40, 119.57, 115.00, 114.19, 103.62, 97.60, 55.61, 55.2, 55.30, 20.85. MS(EI): 431.1 ($C_{27}H_{26}O_5$, $[M+H]^+$). Anal. Calcd for $C_{27}H_{26}O_5$: C, 75.33 ;H,6.09; O, 18.58%. Found: C, 75.41; H, 6.06; O, 18.53%.

*1.1.3. 2,3-dimethylphenyl (E)-3-(2,4-dimethoxy-6-((E)-4-methoxystyryl) phenyl) acrylate **(D3)***

Yellow solid, yield 78%, Mp: 98-100 °C. ^{1}H NMR (600 MHz, $CDCl_3$): δ 8.24 (d, J = 16.0 Hz, 1H), 7.47 (d, J = 8.6 Hz, 2H), 7.33 (d, J = 16.0 Hz, 1H), 7.12 (t, J = 7.8 Hz, 1H), 7.04 (d, J = 7.5 Hz, 1H), 6.95 (s, 2H), 6.90 (d, J = 8.6 Hz, 2H), 6.76 (d, J = 16.0 Hz, 1H), 6.73 (d, J = 1.9 Hz, 1H), 6.45 (d, J = 1.8 Hz, 1H), 3.92 (s, 3H), 3.90 (s, 3H), 3.83 (s, 3H), 2.31 (s, 3H), 2.13 (s, 3H). ^{13}C NMR (150 MHz, $CDCl_3$): δ 166.57, 161.67, 160.83, 159.65, 149.52, 141.77, 140.26, 138.32, 132.20, 129.78, 128.91, 128.04, 127.16, 125.92, 124.87, 119.63, 119.31, 114.98, 114.19,103.64, 97.62, 55.63, 55.44, 55.32, 20.06, 12.48. MS (EI): 445.2. ($C_{28}H_{28}O_5$, $[M+H]^+$). Anal. Calcd for $C_{28}H_{28}O_5$: C, 75.65; H,6.35; O, 18.00%. Found: C, 75.63; H, 6.32; O, 18.05%.

*1.1.4. 2,4-dimethylphenyl (E)-3-(2,4-dimethoxy-6-((E)-4-methoxystyryl) phenyl) acrylate (**D4**)*

Yellow solid, yield 79%, Mp: 97-99 °C. ^{1}H NMR (600 MHz, $CDCl_3$): δ 8.23 (d, J = 16.0 Hz, 1H), 7.47 (d, J = 8.6 Hz, 2H), 7.33 (d, J = 16.0 Hz, 1H), 7.05 (s, 1H), 7.02 (d, J = 8.2 Hz, 1H), 6.98 (d, J = 8.1 Hz, 1H), 6.93 (d, J = 16.0 Hz, 1H), 6.90 (d, J = 8.7 Hz, 2H), 6.74 (d, J = 16.1 Hz,2H), 6.45 (s, 1H), 3.91 (s, 3H), 3.90 (s, 3H), 3.83 (s, 3H), 2.32 (s, 3H), 2.19(s, 3H).^{13}C NMR(151MHz,$CDCl_3$): δ 166.51, 161.64, 160.80, 159.64, 147.38, 141.73,140.18, 135.18, 132.17, 131.60, 129.84, 129.78, 128.03, 127.30, 124.88, 121.71, 119.35, 114.98, 114.18, 103.63, 97.60, 55.61, 55.43, 55.30, 20.80, 16.17. MS (EI): 445.2. ($C_{28}H_{28}O_5$, $[M+H]^+$). Anal. Calcd for $C_{28}H_{28}O_5$: C, 75.65; H,6.35; O, 18.00%. Found: C, 75.61; H, 6.34; O, 18.05%.

*1.1.5. 2,5-dimethylphenyl (E)-3-(2,4-dimethoxy-6-((E)-4-methoxystyryl) phenyl) acrylate (**D5**)*

Yellow solid, yield 75%, Mp: 116-119 °C. ^{1}H NMR (600 MHz, $CDCl_3$): δ 8.24 (d, J = 16.0 Hz, 1H), 7.47 (d, J = 8.1 Hz, 2H), 7.33 (d, J = 16.0 Hz, 1H), 7.13 (d, J = 7.6 Hz, 1H), 6.99-6.92 (m, 3H), 6.90 (d, J = 8.0 Hz, 2H), 6.79-6.70 (m, 2H), 6.45 (s, 1H), 3.92 (s, 3H), 3.90 (s, 3H), 3.83 (s, 3H), 2.33 (s, 3H), 2.19 (s, 3H). ^{13}C NMR (151 MHz, $CDCl_3$): δ 166.42, 161.66, 160.81, 159.64, 149.44, 141.75, 140.24, 136.66, 132.19, 130.66, 129.76, 128.03, 127.00, 126.46, 124.85, 122.57, 119.28, 114.95,

114.18, 103.63, 97.60, 55.61, 55.42, 55.29, 20.87, 15.81. MS (EI): 445.2. ($C_{28}H_{28}O_5$, $[M+H]^+$).Anal. Calcd for $C_{28}H_{28}O_5$: C,75.65 ;H,6.35; O, 18.00%. Found: C, 75.59; H, 6.37; O, 18.05%.

*1.1.6 3,4-dimethylphenyl (E)-3-(2,4-dimethoxy-6-((E)-4-methoxystyryl) phenyl) acrylate (**D6**)*

White solid, yield 74%, Mp: 102-105 °C. ^{1}H NMR (600 MHz, $CDCl_3$): δ 8.20 (d, J = 16.0 Hz, 1H), 7.47 (d, J = 8.6 Hz, 2H), 7.32 (d, J = 16.0 Hz, 1H), 7.14 (d, J = 8.1 Hz, 1H), 6.98-6.92 (m, 2H), 6.90 (d, J = 8.6 Hz, 3H), 6.72 (s, 1H), 6.70 (d, J = 16.0 Hz, 1H), 6.44 (s, 1H), 3.91 (s, 3H), 3.90 (s, 3H), 3.83 (s, 3H), 2.27 (s, 3H), 2.25 (s, 3H).^{13}C NMR (151 MHz, $CDCl_3$): δ 166.89, 161.61, 160.74, 159.63, 148.98, 141.67, 140.11, 137.69, 133.73, 132.16, 130.21, 129.80, 128.08, 124.88, 122.66, 119.68, 118.76, 115.04, 114.19, 103.60, 97.60, 55.62, 55.43, 55.31, 19.85, 19.17. MS (EI): 445.2. ($C_{28}H_{28}O_5$, $[M+H]^+$). Anal. Calcd for $C_{28}H_{28}O_5$: C, 75.65; H, 6.35; O, 18.00%. Found: C, 75.61; H, 6.37; O, 18.02%.

*1.1.7. 3,5-dimethylphenyl (E)-3-(2,4-dimethoxy-6-((E)-4-methoxystyryl) phenyl) acrylate (**D7**)*

Yellow oil, yield 74%. ^{1}H NMR (600 MHz, $CDCl_3$): δ 8.19 (d, J = 16.0 Hz, 1H), 7.47 (d, J = 8.6 Hz, 2H), 7.31 (d, J = 16.0 Hz, 1H), 6.94 (d, J = 16.0 Hz, 1H), 6.90 (d, J = 8.6 Hz, 2H), 6.86 (s, 1H), 6.80 (s, 2H), 6.72 (d, J = 1.9 Hz, 1H), 6.69 (d, J = 16.0 Hz, 1H), 6.44 (d, J = 1.7 Hz, 1H), 3.91 (s, 3H), 3.90 (s, 3H), 3.83 (s, 3H), 2.33 (s, 6H).^{13}C NMR (151 MHz, $CDCl_3$): δ 166.80, 161.63, 160.76, 159.65, 150.98, 141.70, 140.17, 139.08, 132.19, 129.80, 128.09, 127.24, 124.87, 119.65, 119.33, 115.04, 114.19, 103.61, 97.61, 55.62, 55.44, 55.32, 21.23. MS (EI): 445.2. ($C_{28}H_{28}O_5$, $[M+H]^+$). Anal. Calcd for $C_{28}H_{28}O_5$: C, 75.65; H,6.35; O, 18.00%. Found: C, 75.73; H, 6.33; O, 17.96%.

*1.1.8. 2,3,5-trimethylphenyl (E)-3-(2,4-dimethoxy-6-((E)-4-methoxystyryl) phenyl) acrylate (**D8**)*

White solid, yield 77%, Mp: 127-129 °C. ^{1}H NMR (600 MHz, $CDCl_3$): δ 8.24 (d, J = 16.0 Hz, 1H), 7.47 (d, J = 8.5 Hz, 2H), 7.34 (d, J = 16.0 Hz, 1H), 6.94 (d, J = 16.0 Hz, 1H), 6.90 (d, J = 8.6 Hz, 2H), 6.88 (s, 1H), 6.78 (s, 1H), 6.76 (d, J = 16.0 Hz, 1H),

6.73 (s, 1H), 6.45 (s, 1H), 3.92 (s, 3H), 3.90 (s, 3H), 3.83 (s, 3H), 2.30 (s, 3H), 2.27 (s, 3H), 2.09 (s, 3H).^{13}C NMR (151 MHz, $CDCl_3$): *δ* 166.66, 161.63, 160.80, 159.63, 149.35, 141.72, 140.16, 137.91, 135.68, 132.15, 129.77, 128.14, 128.03, 125.57, 124.86, 120.07, 119.39, 114.98, 114.17, 103.60, 97.59, 55.60, 55.41, 55.29, 20.81, 19.98, 12.13. MS (EI): 459.2. ($C_{29}H_{30}O_5$, $[M+H]^+$). Anal. Calcd for $C_{29}H_{30}O_5$: C, 75.96; H, 6.59; O, 17.45%. Found: C, 76.03; H, 6.57; O, 17.40%.

*1.1.9. 4-chlorophenyl (E)-3-(2,4-dimethoxy-6-((E)-4-methoxystyryl) phenyl) acrylate **(D9)***

Yellow solid, yield 81%, Mp: 105-107 °C. ^{1}H NMR (600 MHz, $CDCl_3$): *δ* 8.20 (d, *J* = 16.0 Hz, 1H), 7.69 (d, *J* = 8.6 Hz, 2H), 7.46 (d, *J* = 8.4 Hz, 2H), 7.29 (d, *J* = 16.0 Hz, 1H), 7.00-6.86 (m, 5H), 6.72 (s, 1H), 6.68 (d, *J* = 16.0 Hz, 1H), 6.44 (s, 1H), 3.91 (s, 3H), 3.90 (s, 3H), 3.83 (s, 3H).^{13}C NMR (151 MHz, $CDCl_3$): *δ* 168.88, 164.50, 163.54, 162.37, 153.63, 144.61, 143.53, 140.98, 135.08, 132.38, 130.75, 127.42, 126.65, 121.49, 117.49, 116.88, 106.43, 100.29, 92.05, 58.31, 58.13, 58.00. MS (EI): 451.1 ($C_{26}H_{23}ClO_5$, $[M+H]^+$). Anal. Calcd for $C_{26}H_{23}ClO_5$: C, 69.25; H, 5.14; O, 17.74%. Found: C, 69.31; H, 5.12; O, 17.70%.

*1.1.10. 4-iodophenyl (E)-3-(2,4-dimethoxy-6-((E)-4-methoxystyryl) phenyl) acrylate **(D10)***

Yellow solid, yield 80%, Mp: 94-97 °C. ^{1}H NMR (600 MHz, $CDCl_3$): *δ* 8.21 (d, *J* = 16.0 Hz, 1H), 7.47 (d, *J* = 8.5 Hz, 2H), 7.35 (d, *J* = 8.7 Hz, 2H), 7.30 (d, *J* = 16.0 Hz, 1H), 7.12 (d, *J* = 8.7 Hz, 2H), 6.93 (d, *J* = 16.2 Hz, 1H), 6.90 (d, *J* = 8.5 Hz, 2H), 6.72 (s, 1H), 6.69 (d, *J* = 16.0 Hz, 1H), 6.44 (s, 1H), 3.91 (s, 3H), 3.90 (s, 3H), 3.83 (s, 3H).^{13}C NMR (151 MHz, $CDCl_3$): *δ* 166.36, 161.83, 160.87, 159.71, 149.58, 141.94, 140.83, 132.40, 130.77, 129.73, 129.32, 128.09, 124.77, 123.13, 118.84, 114.83, 114.22, 103.76, 97.63, 55.65, 55.46, 55.33. MS (EI): 543.1 ($C_{26}H_{23}IO_5$, $[M+H]^+$).Anal. Calcd for $C_{26}H_{23}IO_5$: C, 57.58; H, 4.27; O, 14.75 %. Found: C, 57.58; H, 4.26; O, 17.73%.

*1.1.11.4-chloro-3,5-dimethylphenyl(E)-3-(2,4-dimethoxy-6-((E)-4-methoxystyryl)phenyl)acrylate (**D11**)*

Yellow solid, yield 81%, Mp: 93-95 °C. ^{1}H NMR (600 MHz, $CDCl_3$): *δ* 8.19 (d,

J = 16.0 Hz, 1H), 7.47 (d, *J* = 8.6 Hz, 2H), 7.30 (d, *J* = 16.0Hz, 1H), 6.93 (d, *J* = 16.5 Hz, 3H), 6.90 (d, *J* = 8.6 Hz, 2H), 6.72 (d, *J* = 1.9 Hz, 1H), 6.68 (d, *J* = 16.0 Hz, 1H), 6.44 (d, *J* = 1.8 Hz, 1H), 3.91 (s, 3H), 3.90 (s, 3H), 3.83 (s, 3H), 2.38 (s, 6H).^{13}C NMR (151 MHz, $CDCl_3$): δ 166.65, 161.75, 160.83, 159.68, 148.68, 141.84, 140.57, 137.26, 132.32, 131.33, 129.75, 128.09, 124.80, 121.55, 119.12, 114.90, 114.20, 103.70, 97.61, 55.63, 55.44, 55.32, 20.80. MS (EI): 479.1 ($C_{28}H_{27}ClO_5$, $[M+H]^+$). Anal. Calcd for $C_{28}H_{27}ClO_5$: C, 70.21; H, 5.68; O, 16.70 %. Found: C, 70.23; H, 5.69; O, 16.68%.

*1.1.12 3-(dimethylamino) phenyl (E)-3-(2,4-dimethoxy-6-((E)-4-methoxystyryl) phenyl) acrylate (**D12**)*

Purple oil, yield 72%. ^{1}H NMR (600 MHz, $CDCl_3$): δ 8.20 (d, *J* = 16.0 Hz, 1H), 7.47 (d, *J* = 8.5 Hz, 2H), 7.32 (d, *J* = 16.0 Hz, 1H), 7.23 (t, *J* = 8.1 Hz, 1H), 6.94 (d, *J* = 16.0 Hz, 1H), 6.90 (d, *J* = 8.5 Hz, 2H), 6.71 (d, *J* = 16.3 Hz,2H), 6.59 (d, *J* = 7.1 Hz, 1H), 6.54 (d, *J* = 7.7 Hz, 1H), 6.51 (s, 1H), 6.44 (s, 1H), 3.91 (s, 3H), 3.90 (s, 3H), 3.83 (s, 3H), 2.95 (s, 6H).^{13}C NMR (151 MHz, $CDCl_3$): δ 166.72, 161.59, 160.74, 159.63, 152.15, 141.67, 140.03, 132.16, 129.80, 129.52, 128.09, 124.90, 119.84, 115.07, 114.19, 109.75, 109.56, 105.80, 103.59, 97.61, 55.62, 55.43, 55.31, 40.50. MS (EI): 460.2 ($C_{28}H_{29}NO_5$, $[M+H]^+$). Anal. Calcd for $C_{28}H_{29}NO_5$: C, 73.18; H, 6.36; O, 17.41%. Found: C, 73.20; H, 6.38; O, 17.38%.

*1.1.13. 3,5-dimethoxyphenyl (E)-3-(2,4-dimethoxy-6-((E)-4-methoxystyryl) phenyl) acrylate (**D13**)*

Yellow oil, yield 78%. ^{1}H NMR (600 MHz, $CDCl_3$): δ 8.19 (d, *J* = 16.0 Hz, 1H), 7.47 (d, *J* = 8.5 Hz, 2H), 7.31 (d, *J* = 15.9 Hz, 1H), 6.93 (d, *J* = 16.1 Hz, 1H), 6.90 (d, *J* = 8.5 Hz, 2H), 6.72 (s, 1H), 6.69 (d, *J* = 16.0 Hz, 1H), 6.44 (s, 1H), 6.35 (s, 3H), 3.91 (s, 3H), 3.90 (s, 3H), 3.83 (s, 3H), 3.78 (s, 6H),.^{13}C NMR (151 MHz, $CDCl_3$): δ 166.47, 161.70, 161.02, 160.80, 152.63, 140.49, 132.29, 128.10, 124.81, 119.26, 114.18, 103.61, 100.30, 99.90, 98.18, 97.60, 55.64, 55.46, 55.34, 55.26. MS (EI): 477.2 ($C_{28}H_{28}O_7$, $[M+H]^+$). Anal. Calcd for $C_{28}H_{28}O_7$: C, 70.57; H, 5.92; O, 23.50%. Found: C, 70.61; H, 5.93; O, 23.46%.

1.1.14 4-allyl-2-methoxyphenyl (E)-3-(2,4-dimethoxy-6-((E)-4-methoxystyryl) phenyl)

*acrylate (**D14**)*

Yellow oil, yield 81%. ^{1}H NMR (600 MHz, $CDCl_3$): δ 8.22 (d, J = 16.0 Hz, 1H), 7.46 (d, J = 8.5 Hz, 2H), 7.34 (d, J = 16.0 Hz, 1H), 7.03 (d, J = 7.9 Hz, 1H), 6.92 (d, J = 16.1 Hz, 1H), 6.89 (s, 1H), 6.88 (s, 1H), 6.80 (dd, J = 15.9 ,5.7Hz, 3H), 6.72 (s, 1H), 6.44 (s, 1H), 5.98 (d, J = 6.9 Hz,1H), 5.11 (t, J = 13.7 Hz, 2H), 3.90 (s, 3H), 3.89 (s, 3H), 3.82 (s, 6H), 3.39 (d, J = 6.6 Hz, 1H).^{13}C NMR (151 MHz, $CDCl_3$): δ 166.50, 161.58, 160.91, 159.57, 151.11, 141.85, 140.23, 138.61, 138.26, 137.16, 132.18, 129.79, 128.08, 124.85, 122.78, 120.62, 118.93, 116.02, 114.93, 114.12, 112.70, 103.51, 97.56, 55.88, 55.57, 55.43, 55.30, 40.09. MS (EI): 487.2 ($C_{30}H_{30}O_6$, $[M+H]^+$). Anal. Calcd for $C_{30}H_{30}O_6$: C, 74.06; H, 6.21; O, 19.73%. Found: C, 73.99; H, 6.23; O, 19.78%.

*1.1.15. benzo[d] [1,3] dioxol-5-yl (E)-3-(2,4-dimethoxy-6-((E)-4-methoxystyryl) phenyl) acrylate (**D15**)*

Yellow oil, yield 75%. ^{1}H NMR (600 MHz, $CDCl_3$): δ 8.19 (d, J = 16.0 Hz, 1H), 7.47 (d, J = 8.5 Hz, 2H), 7.30 (d, J = 16.0 Hz, 1H), 6.92 (dd, J = 17.0,12.4 Hz,3H), 6.79 (d, J = 8.3 Hz, 1H), 6.72 (s, 1H), 6.70 (s, 1H), 6.68 (d, J = 16.0 Hz, 1H), 6.61 (dd, J = 8.3,1.9 Hz, 1H), 6.44 (s, 1H), 5.98 (s, 2H), 3.91 (s, 3H), 3.90 (s, 3H), 3.83 (s, 3H).^{13}C NMR (151 MHz, $CDCl_3$): δ 166.91, 161.67, 160.76, 159.62, 147.86, 145.36, 145.07, 141.77, 140.44, 132.24, 129.71, 128.07, 124.78, 119.16, 114.86, 114.17, 114.31, 107.88, 103.97, 103.59, 101.58, 97.57, 55.62, 55.44, 55.31. MS (EI): 461.2($C_{27}H_{24}O_7$, $[M+H]^+$). Anal. Calcd for $C_{27}H_{24}O_7$: C, 70.43; H, 5.25; O, 24.32%. Found: C, 70.38; H, 5.24; O, 24.38%.

*1.1.16. 4-cyclohexylphenyl (E)-3-(2,4-dimethoxy-6-((E)-4-methoxystyryl) phenyl) acrylate (**D16**)*

Yellow solid, yield 75%, Mp: 109-111 °C. ^{1}H NMR (600 MHz, $CDCl_3$): δ 8.20 (d, J = 16.0 Hz, 1H), 7.47 (d, J = 8.2 Hz, 2H), 7.32 (d, J = 16.0 Hz, 1H), 7.22 (d, J = 7.9 Hz, 2H), 7.08 (d, J = 8.1 Hz, 2H), 6.93 (d, J = 16.0 Hz, 1H), 6.90 (d, J = 8.0 Hz, 2H), 6.72 (m, 2H), 6.44 (s, 1H), 3.91 (s, 3H), 3.90 (s, 3H), 3.83 (s, 3H), 2.51 (m, 1H), 1.92-1.80 (m, 4H), 1.40 (s, 4H), 1.26 (s, 2H).^{13}C NMR(151 MHz,$CDCl_3$): δ 166.82, 161.60, 160.74, 159.60, 148.93, 145.22, 141.69, 140.18, 132.16, 129.74, 128.07,

127.57, 124.81, 121.33, 119.56, 114.96, 114.16, 103.53, 97.57, 77.21, 77.00, 76.79, 55.62, 55.44, 55.31, 44.00, 34.50, 26.86, 26.11. MS (EI): 499.3 ($C_{32}H_{34}O_5$, H]$^+$). Anal. Calcd for $C_{32}H_{34}O_5$: C, 77.08; H, 6.87; O, 16.04%. Found: C, 70.07; H, 6.85; O, 16.08%.

*1.1.17. naphthalen-1-yl (E)-3-(2,4-dimethoxy-6-((E)-4-methoxystyryl) phenyl) acrylate (**D17**)*

Yellow olid, yield 72%. ^{1}H NMR (600 MHz, $CDCl_3$): δ 8.36 (d, J = 16.0 Hz, 1H), 7.99 (d, J = 7.8 Hz, 1H), 7.88 (d, J = 7.9 Hz, 1H), 7.75 (d, J = 8.2 Hz, 1H), 7.50 (s, 5H), 7.38 (d, J = 12.8 Hz, 1H), 7.36 (d, J = 4.2 Hz, 1H), 6.97 (d, J = 16.0 Hz, 1H), 6.92 (d, J = 18.6 Hz, 3H), 6.76 (s, 1H), 6.48 (s, 1H), 3.95 (s, 3H), 3.91 (s, 3H), 3.81 (s, 3H).^{13}C NMR (151 MHz, $CDCl_3$): δ 166.66, 161.80, 160.94, 159.66, 146.98, 141.95, 140.79, 134.62, 132.36, 129.74, 128.06, 127.90, 127.10, 126.27, 126.25, 125.62, 125.43, 124.80, 121.48, 118.94, 118.15, 114.89, 114.19, 103.72, 97.62, 55.64, 55.44, 55.28. MS (EI): 467.2 ($C_{30}H_{26}O_5$, [M+H]$^+$). Anal. Calcd for $C_{30}H_{26}O_5$: C, 77.24; H, 5.62; O, 17.15%. Found: C, 77.23; H, 5.60; O, 17.17%.

*1.1.18. naphthalen-2-yl (E)-3-(2,4-dimethoxy-6-((E)-4-methoxystyryl) phenyl) acrylate (**D18**)*

Yellow solid, yield 70%, Mp: 120-122 °C. ^{1}H NMR (600 MHz, $CDCl_3$): δ 8.27 (d, J = 16.0 Hz, 1H), 7.86 (t, J = 8.9 Hz, 2H), 7.82 (d, J = 7.9 Hz, 1H), 7.66 (s, 1H), 7.48 (m, 4H), 7.35 (d, J = 5.9 Hz, 1H), 7.33 (s, 1H), 6.95 (d, J = 16.0 Hz, 1H), 6.91 (d, J = 8.3 Hz, 2H), 6.78 (d, J = 16.0 Hz, 1H), 6.74 (s, 1H), 6.46 (s, 1H), 3.93 (s, 3H), 3.91 (s, 3H), 3.82 (s, 3H).^{13}C NMR (151 MHz, $CDCl_3$): δ 166.77, 161.73, 160.84, 159.67, 148.77, 141.84, 140.54, 133.82, 132.30, 131.34, 129.76, 129.18, 128.09, 127.71, 127.63, 126.37, 125.46, 124.83, 121.48, 119.35, 118.58, 114.97, 114.21, 103.70, 97.62, 55.64, 55.44, 55.30. MS (EI): 467.2 ($C_{30}H_{26}O_5$, [M+H]$^+$). Anal. Calcd for $C_{30}H_{26}O_5$: C, 77.24; H, 5.62; O, 17.15%. Found: C, 77.21; H, 5.61; O, 17.18%.

*1.1.19. [1,1'-biphenyl]-2-yl (E)-3-(2,4-dimethoxy-6-((E)-4-methoxystyryl) phenyl) acrylate (**D19**)*

Yellow solid, yield 68%, Mp: 50-52 °C. ^{1}H NMR (600 MHz, $CDCl_3$): δ 8.07 (d, J = 16.0 Hz, 1H), 7.47 (d, J = 7.4 Hz, 2H), 7.44 (d, J = 7.5 Hz, 2H), 7.40 (d, J = 8.8

Hz,3H), 7.35 (t, *J* = 7.6 Hz, 2H), 7.32 (d, *J* = 7.4 Hz, 1H), 7.29 (s, 1H), 7.24 (d, *J* = 7.9 Hz, 1H), 7.19 (d, *J* = 16.0 Hz, 1H), 6.93-6.86 (m, 3H), 6.69 (d, *J* = 1.8 Hz, 1H), 6.53 (d, *J* = 16.0 Hz, 1H), 6.41 (d, *J* = 1.8 Hz, 1H), 3.89 (s, 3H), 3.87 (s, 3H), 3.83 (s, 3H).^{13}C NMR (151 MHz, $CDCl_3$): δ 169.14, 162.29, 144.30, 142.96, 140.34, 137.47, 134.72, 133.47, 131.64, 131.01, 130.82, 130.70, 129.92, 128.67, 127.50, 125.87, 121.98, 117.68, 116.85, 106.26, 100.25, 58.27, 58.10, 57.99. MS (EI): 493.2 ($C_{32}H_{28}O_5$, $[M+H]^+$). Anal. Calcd for $C_{32}H_{28}O_5$: C, 78.03; H, 5.73; O, 16.24%. Found: C, 77.99; H, 5.75; O, 16.26%.

*1.1.20. [1,1'-biphenyl]-3-yl (E)-3-(2,4-dimethoxy-6-((E)-4-methoxystyryl) phenyl) acrylate (**D20**)*

Yellow solid, yield 66%, Mp: 50-52 °C. ^{1}H NMR (600 MHz, $CDCl_3$): δ 8.25 (d, *J* = 16.0Hz, 1H), 7.60 (d, *J* = 7.7 Hz, 2H), 7.47 (s, 7H), 7.36 (d, *J* = 7.1 Hz, 2H), 7.33 (d, *J* = 15.9 Hz, 1H), 7.18 (d, *J* = 3.5 Hz, 1H), 6.95 (d, *J* = 16.0 Hz, 1H), 6.91 (d, *J* = 8.5 Hz, 2H), 6.75 (d, *J* = 16.0 Hz,2H), 6.45 (s, 1H), 3.92 (s, 3H), 3.90 (s, 3H), 3.82 (s, 3H).^{13}C NMR (151 MHz, $CDCl_3$): δ 166.61, 161.72, 160.82, 159.67, 151.48, 142.66, 141.82, 140.51, 140.31, 132.31, 129.76, 129.55, 128.72, 128.09, 127.52, 127.17, 124.83, 124.24, 120.55, 120.53, 19.34, 114.95, 114.21, 103.70, 97.62, 55.64, 55.44, 55.31. MS (EI): 493.2 ($C_{32}H_{28}O_5$, $[M+H]^+$). Anal. Calcd for $C_{32}H_{28}O_5$: C, 78.03; H, 5.73; O, 16.24%. Found: C, 78.00; H, 5.75; O, 16.25%.

*1.1.21. [1,1'-biphenyl]-4-yl (E)-3-(2,4-dimethoxy-6-((E)-4-methoxystyryl) phenyl) acrylate (**D21**)*

White solid, yield 68%, Mp: 99-102 °C. ^{1}H NMR (600 MHz, $CDCl_3$): δ 8.25(d, *J* = 16.0 Hz, 1H), 7.61 (d, *J* = 8.5 Hz, 2H), 7.59 (d, *J* = 8.2 Hz, 2H), 7.48 (d, *J* = 8.5 Hz, 2H), 7.45 (t, *J* = 7.3 Hz, 2H), 7.34 (dd, *J* = 15.0, 10.4 Hz, 2H), 7.26 (d, *J* = 7.3 Hz, 2H), 6.95 (d, *J* = 16.0 Hz, 1H), 6.91 (d, *J* = 8.5 Hz, 2H), 6.75 (d, *J* = 14.7Hz, 2H), 6.46 (s, 1H), 3.92 (s, 3H), 3.91 (s, 3H), 3.83 (s, 3H).^{13}C NMR (151 MHz, $CDCl_3$): δ 166.65, 161.73, 160.83, 159.67, 150.53, 141.82, 140.53, 138.59, 132.29, 129.76, 128.73, 128.05, 127.20, 127.08, 124.82, 122.01, 119.29, 114.94, 114.21, 103.69, 97.61, 77.21, 77.00, 76.79, 55.64, 55.44, 55.31. MS (EI): 493.2 ($C_{32}H_{28}O_5$, $[M+H]^+$). Anal. Calcd for $C_{32}H_{28}O_5$: C, 78.03; H, 5.73; O, 16.24%. Found: C, 78.06; H, 5.73; O,

16.21%.

*1.1.22. 2-benzylphenyl (E)-3-(2,4-dimethoxy-6-((E)-4-methoxystyryl) phenyl) acrylate (**D22**)*

Yellow oil, yield 73%. ^{1}H NMR (600 MHz, $CDCl_3$): δ 8.20 (d, J = 16.0 Hz, 1H), 7.45 (d, J = 8.5 Hz, 2H), 7.30 (d, J = 16.0 Hz, 1H), 7.24 (d, J = 7.4 Hz,3H), 7.19 (d, J = 7.2 Hz,6H), 6.94 (d, J = 16.0 Hz, 1H), 6.87 (d, J = 8.5 Hz, 2H), 6.73 (s, 2H), 6.45 (s, 1H), 3.95 (s, 2H), 3.91 (d, J = 3.8 Hz,6H), 3.82 (s, 3H).^{13}C NMR (151 MHz, $CDCl_3$): δ166.36, 161.68, 160.79, 159.60, 149.17, 141.76, 140.45, 139.91, 133.33, 132.22, 130.59, 129.69, 129.04, 128.35, 128.04, 127.27, 126.05, 125.81, 124.79, 122.55, 119.15, 114.88, 114.16, 103.58, 97.57, 55.61, 55.44, 55.30, 36.17. MS (EI): 507.2 ($C_{33}H_{30}O_5$, $[M+H]^+$). Anal. Calcd for $C_{33}H_{30}O_5$: C, 78.24; H, 5.97; O, 15.79%. Found: C, 78.23; H, 5.96; O, 15.81%.

*1.1.23． 4-(2-phenylpropan-2-yl) phenyl (E)-3-(2,4-dimethoxy-6-((E)-4-methoxystyryl) phenyl) acrylate (**D23**)*

Yellow oil, yield 64%. ^{1}H NMR (600 MHz, $CDCl_3$): δ 8.20 (d, J = 16.0 Hz, 1H), 7.47 (d, J = 8.4 Hz, 2H), 7.32 (d, J = 16.0 Hz, 1H), 7.29 (d, J = 7.9 Hz, 1H), 7.26 (dd, J = 9.3,8.0 Hz,5H), 7.18 (dd, J = 9.7,4.2 Hz, 1H), 7.08 (d, J = 8.5 Hz, 2H), 6.94 (d, J = 16.0 Hz, 1H), 6.91 (d, J = 8.4 Hz, 2H), 6.73 (s, 1H), 6.71 (d, J = 16.0 Hz, 1H), 6.45 (s, 1H), 3.91 (s, 3H), 3.90 (s, 3H), 3.83 (s, 3H), 1.70 (s, 6H).^{13}C NMR (151 MHz, $CDCl_3$): δ166.68, 161.66, 160.77, 159.64, 150.43, 148.86, 147.79, 141.73, 140.26, 132.20, 129.77, 128.06, 127.99, 127.69, 126.77, 125.64, 124.82, 121.02, 119.50, 14.97(s), 114.19, 103.62, 97.60, 55.62, 55.42, 55.31, 42.68, 30.83. MS (EI): 535.3 ($C_{35}H_{34}O_5$, $[M+H]^+$). Anal. Calcd for $C_{35}H_{34}O_5$: C, 78.63; H, 6.41; O, 14.96%.Found: C, 78.70; H, 6.39; O, 14.91%.

2. The spectra of compounds D1-23

D1:

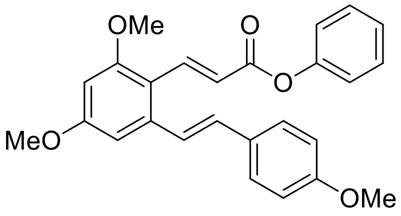

H[1] NMR

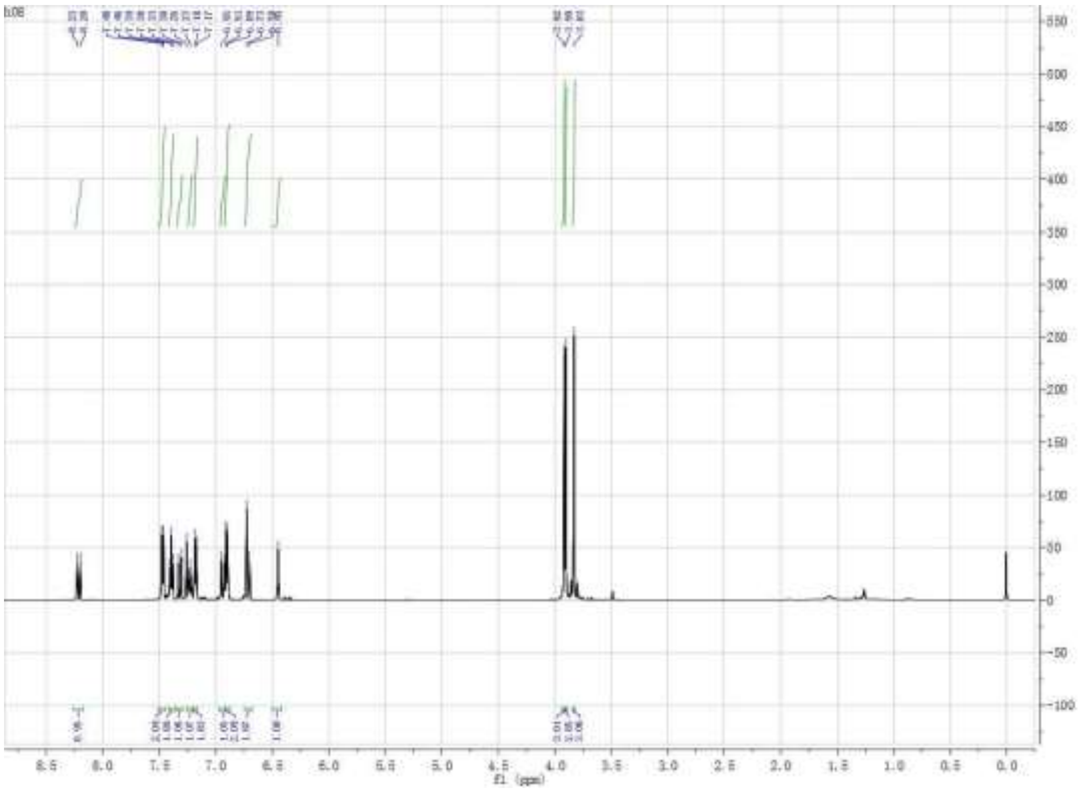

C[13] NMR

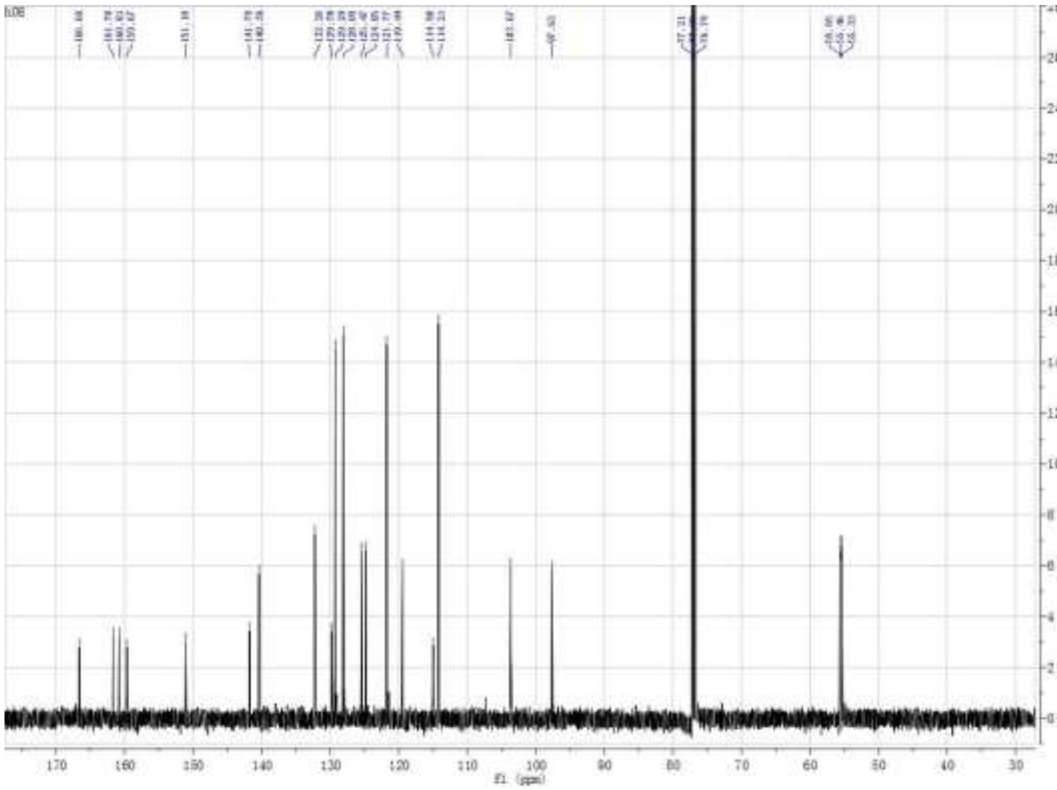

MS(EI):431.1($C_{27}H_{26}O_5$, $[M+H]^+$).

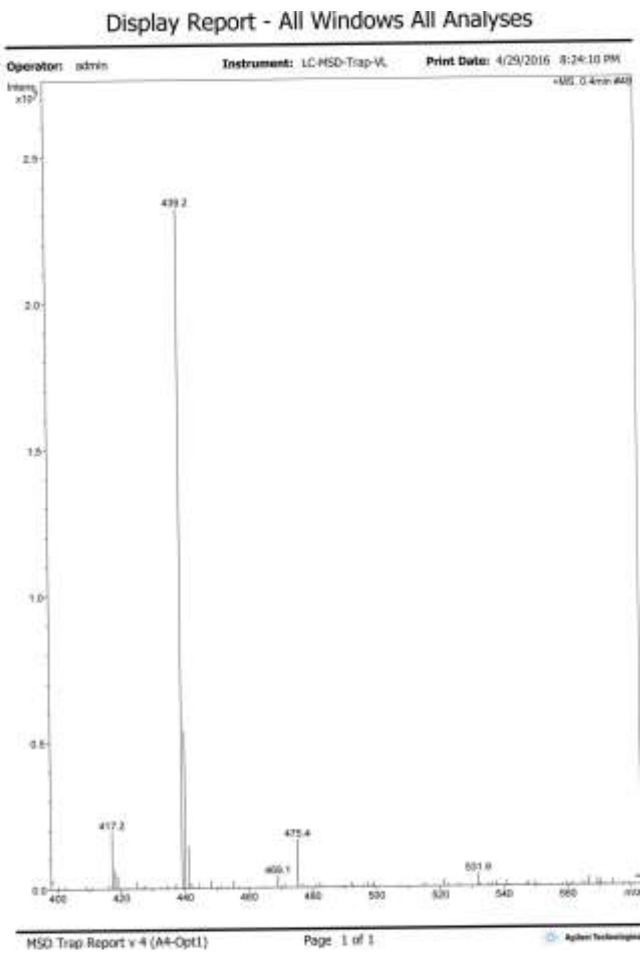
Display Report - All Windows All Analyses
Operator: admin
Instrument: LC-MSD-Trap-VL
Print Date: 4/29/2016 8:24:10 PM
439.2
417.2
475.4
531.8
MSD Trap Report v 4 (A4-Opt1)
Page 1 of 1

D2:

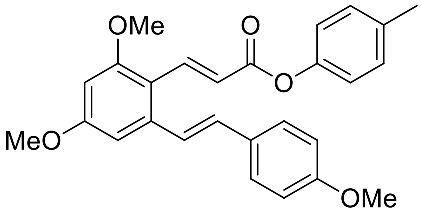
OMe
O
O
MeO
OMe

H1 NMR

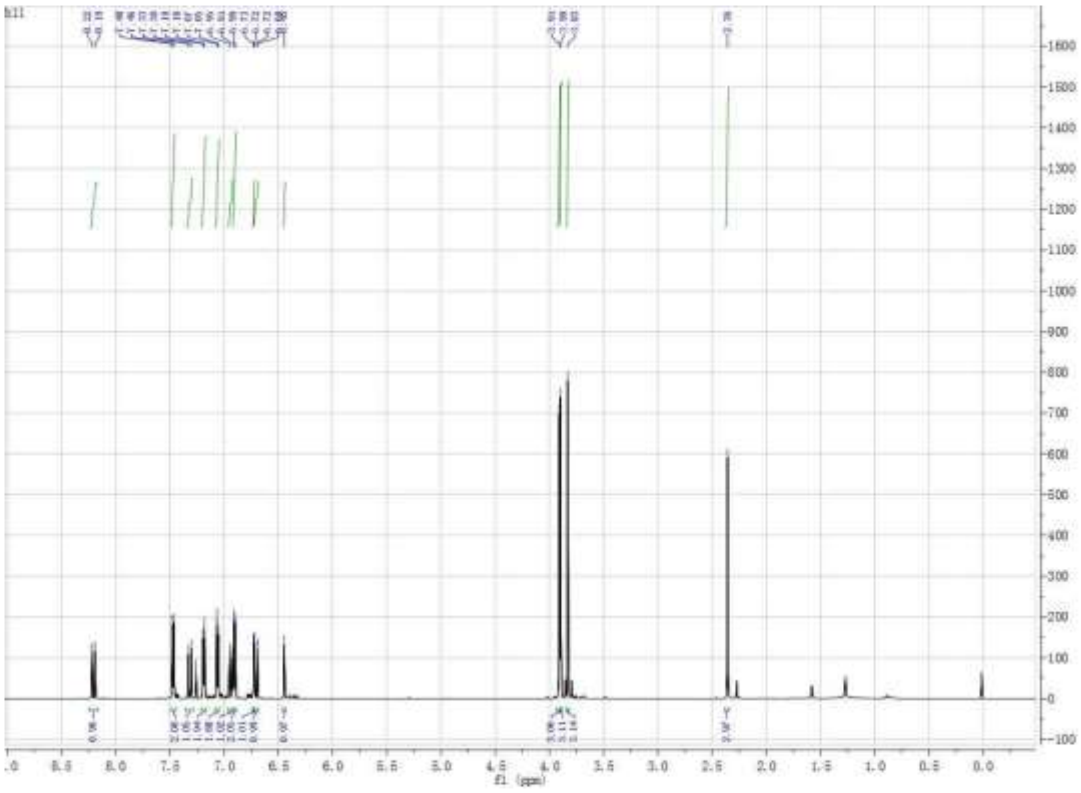

C13 NMR

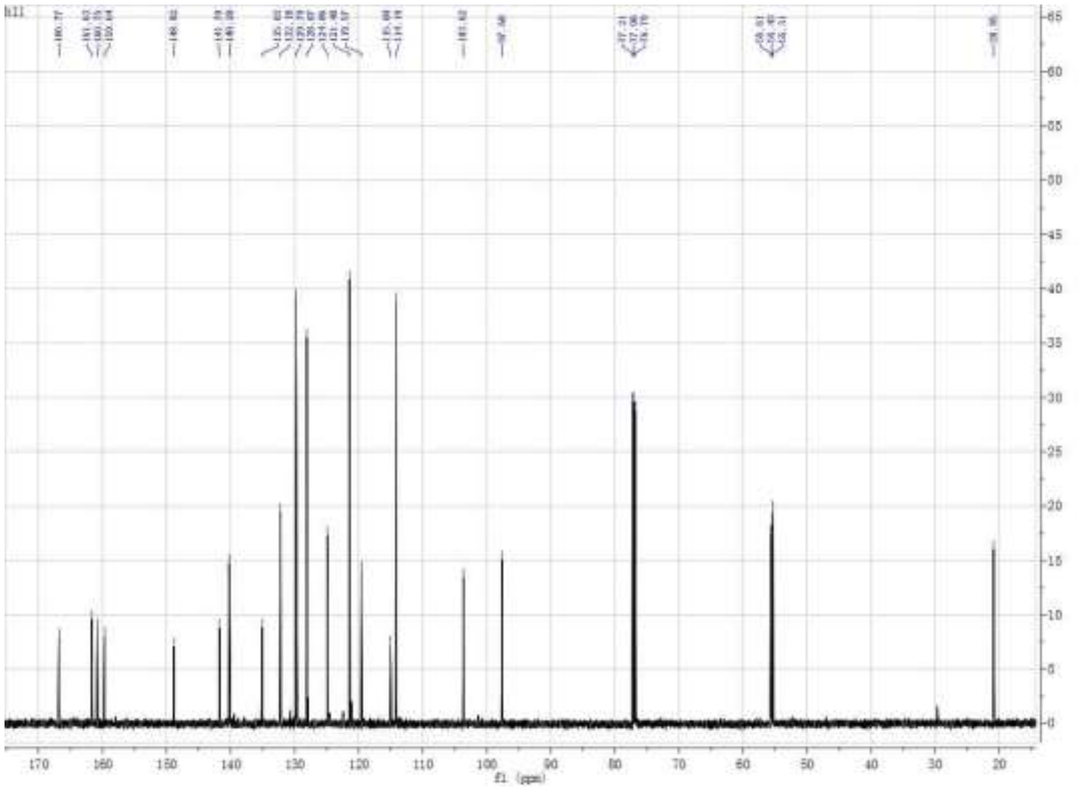

MS(EI):431.1($C_{27}H_{26}O_5$, $[M+H]^+$).

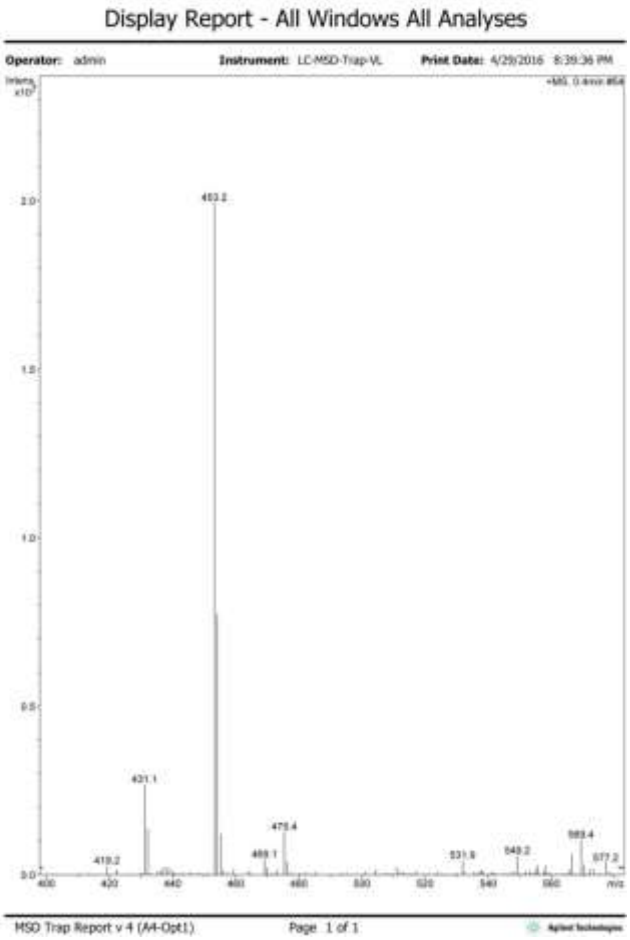
Display Report - All Windows All Analyses
Operator: admin
431.1
475.4
MSD Trap Report v 4 (A4-Opt1)
Page 1 of 1

D3:

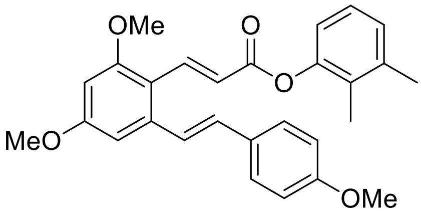
OMe
O
O
MeO
OMe

H^1 NMR

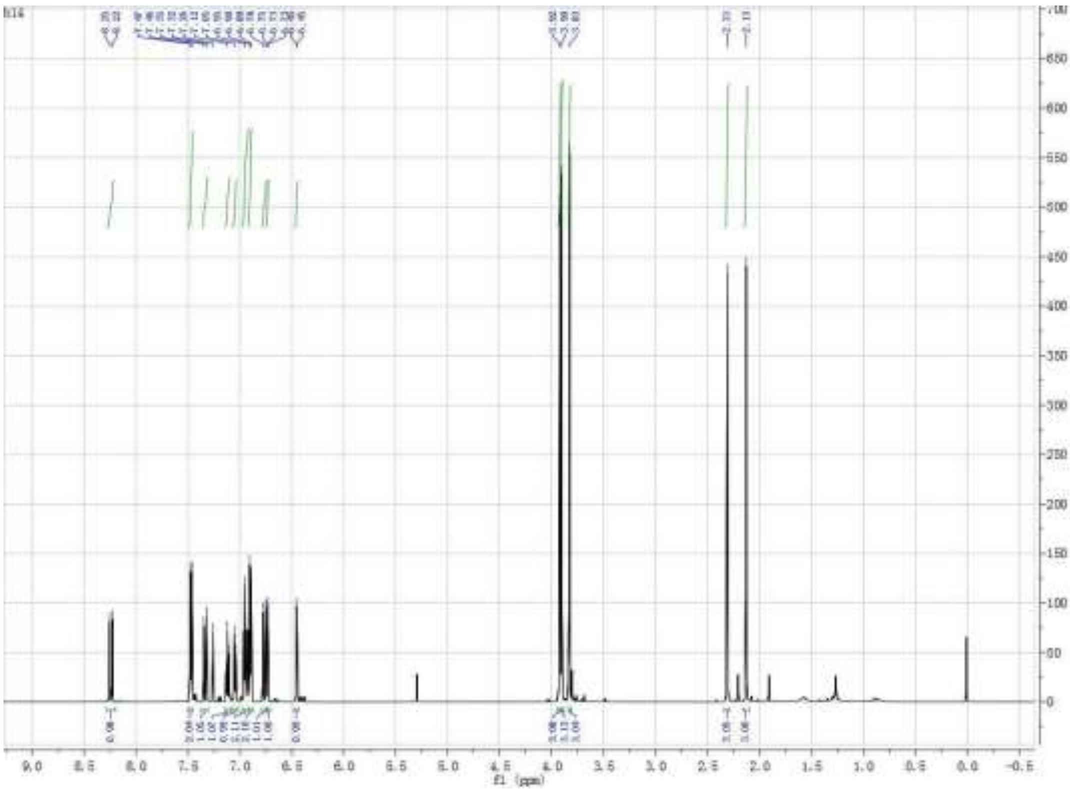

C13 NMR

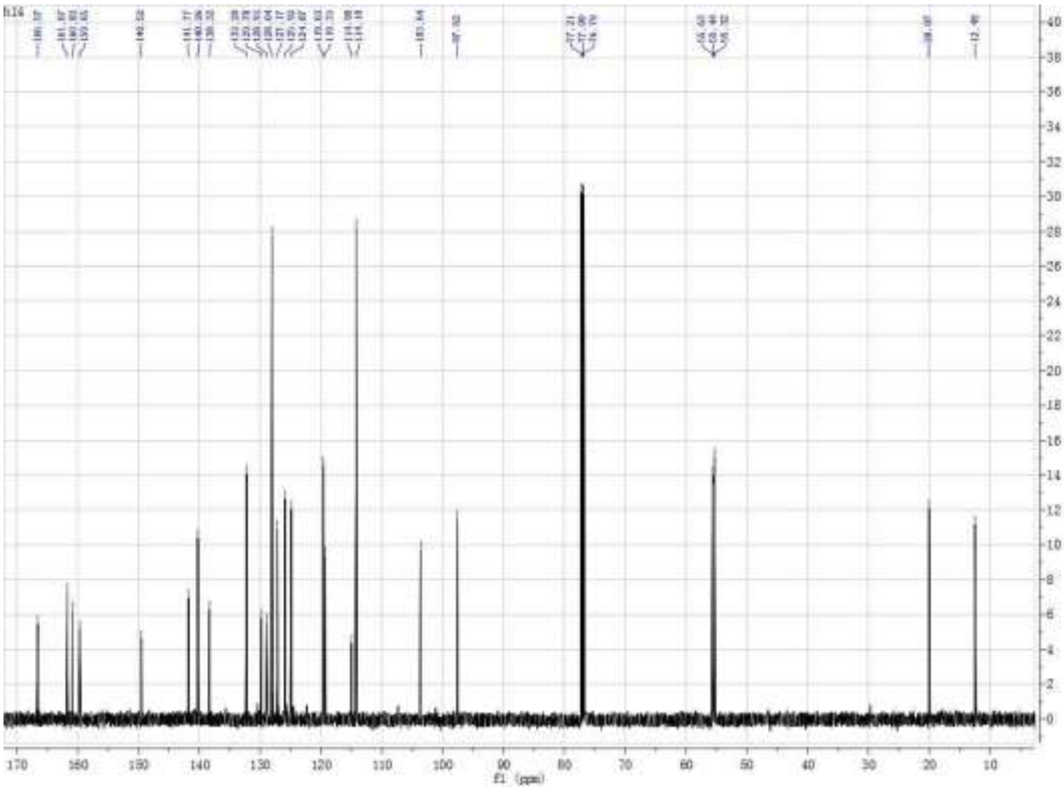

MS (EI): 445.2. ($C_{28}H_{28}O_5$, $[M+H]^+$).

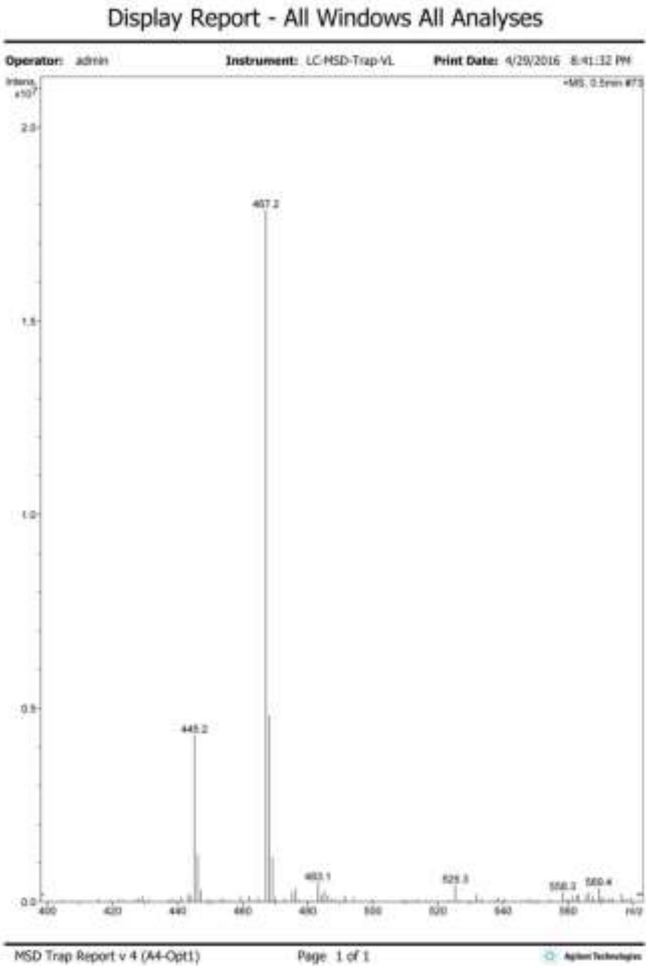
Display Report - All Windows All Analyses
Operator: admin
Instrument: LC-MSD-Trap-VL
Print Date: 4/29/2016 8:41:32 PM
467.2
445.2
483.1
525.3
MSD Trap Report v 4 (A4-Opt1)
Page 1 of 1

D4:

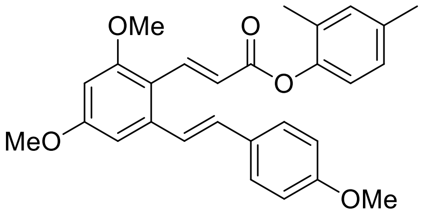
OMe
O
O
MeO
OMe

H[1]

NMR

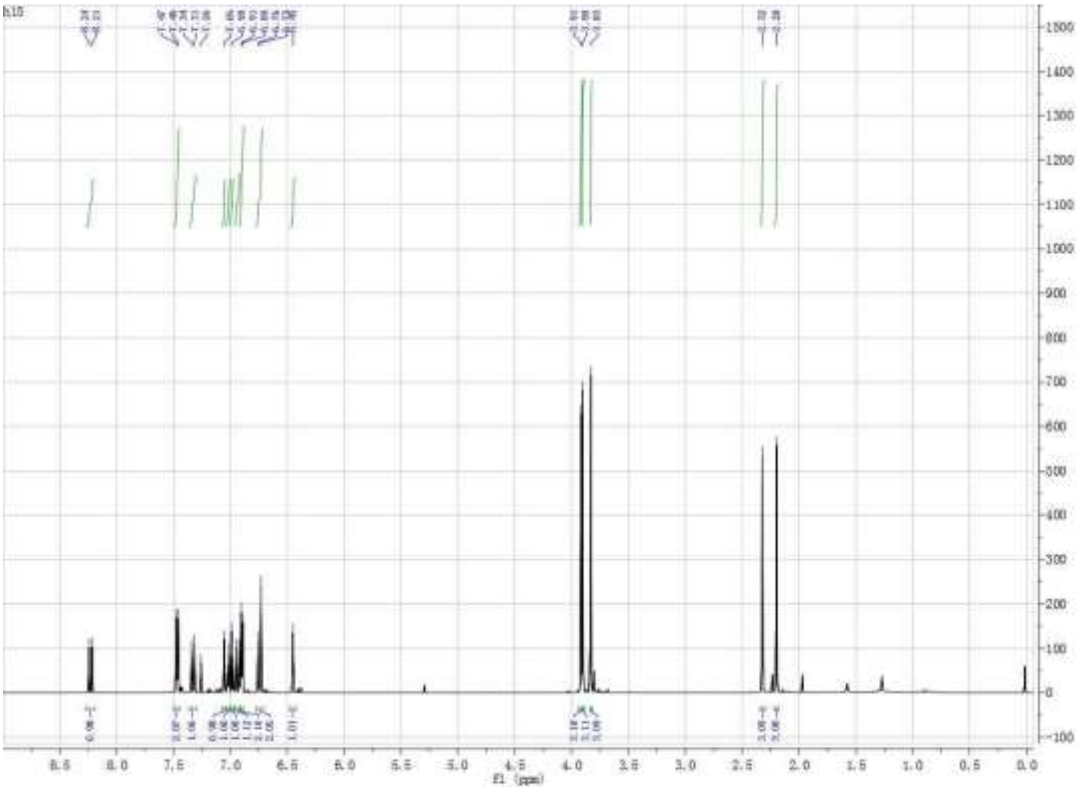

C^{13} NMR

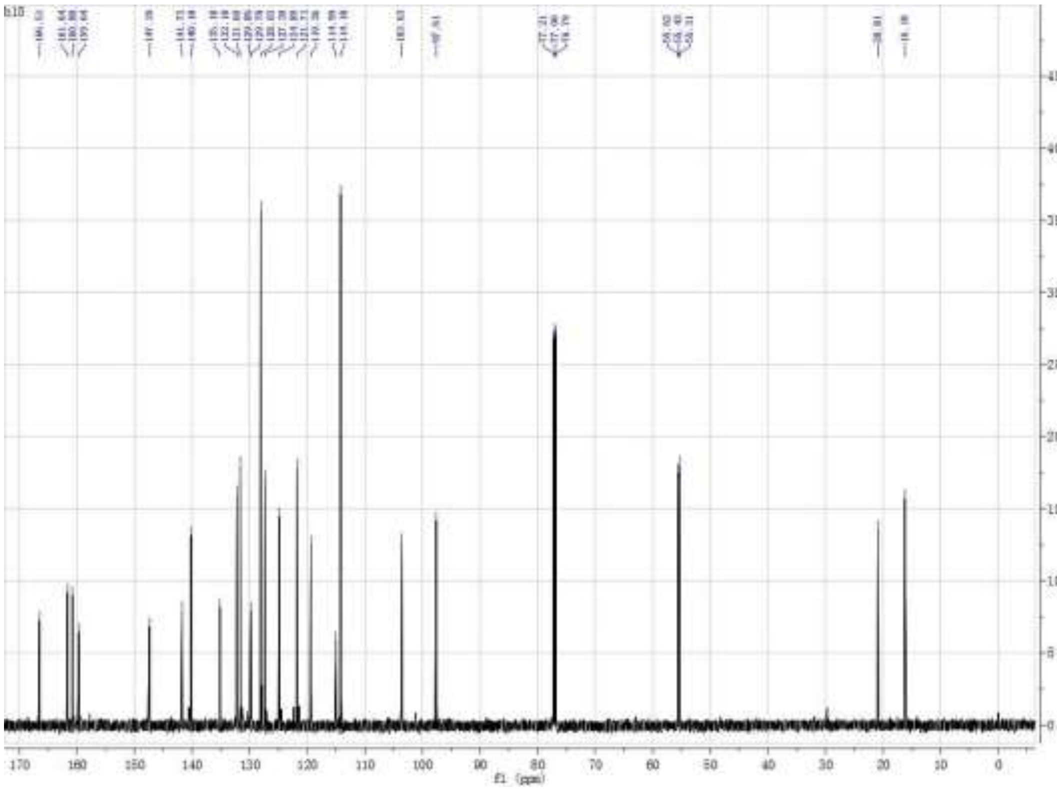

MS (EI):445.2.($C_{28}H_{28}O_5$, $[M+H]^+$).

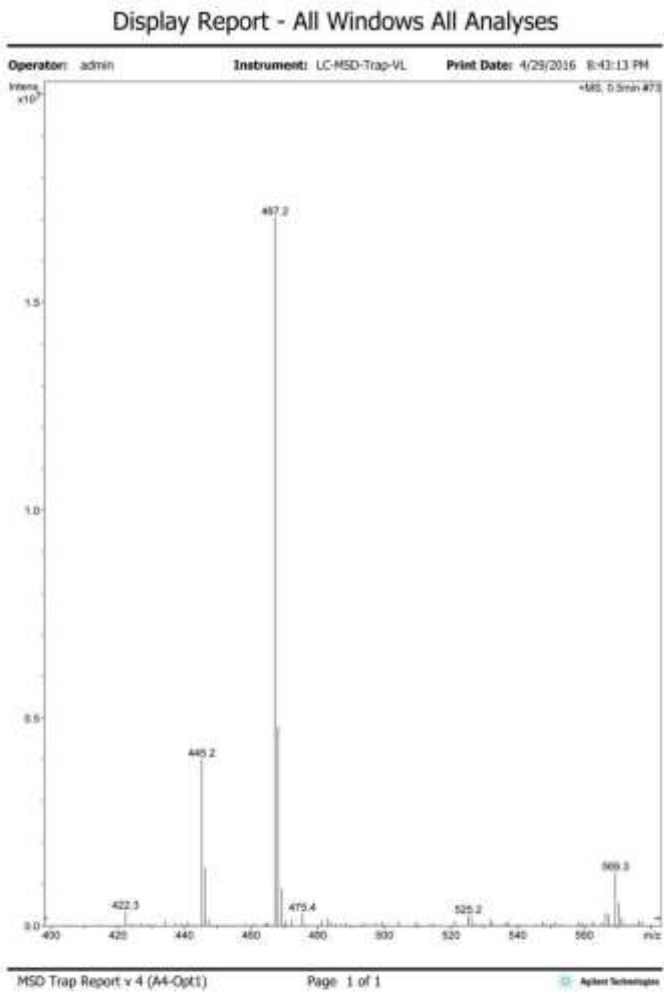
Display Report - All Windows All Analyses
Operator: admin
Instrument: LC-MSD-Trap-VL
Print Date: 4/29/2016 8:43:13 PM
Intens. x10^7
467.2
445.2
422.3
475.4
525.2
569.3
m/z
MSD Trap Report v 4 (A4-Opt1)
Page 1 of 1

D5:

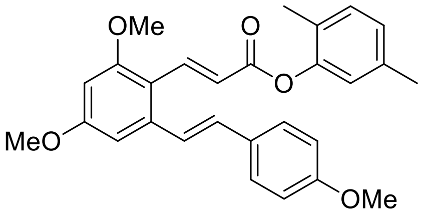
OMe
O
O
MeO
OMe

H1 NMR

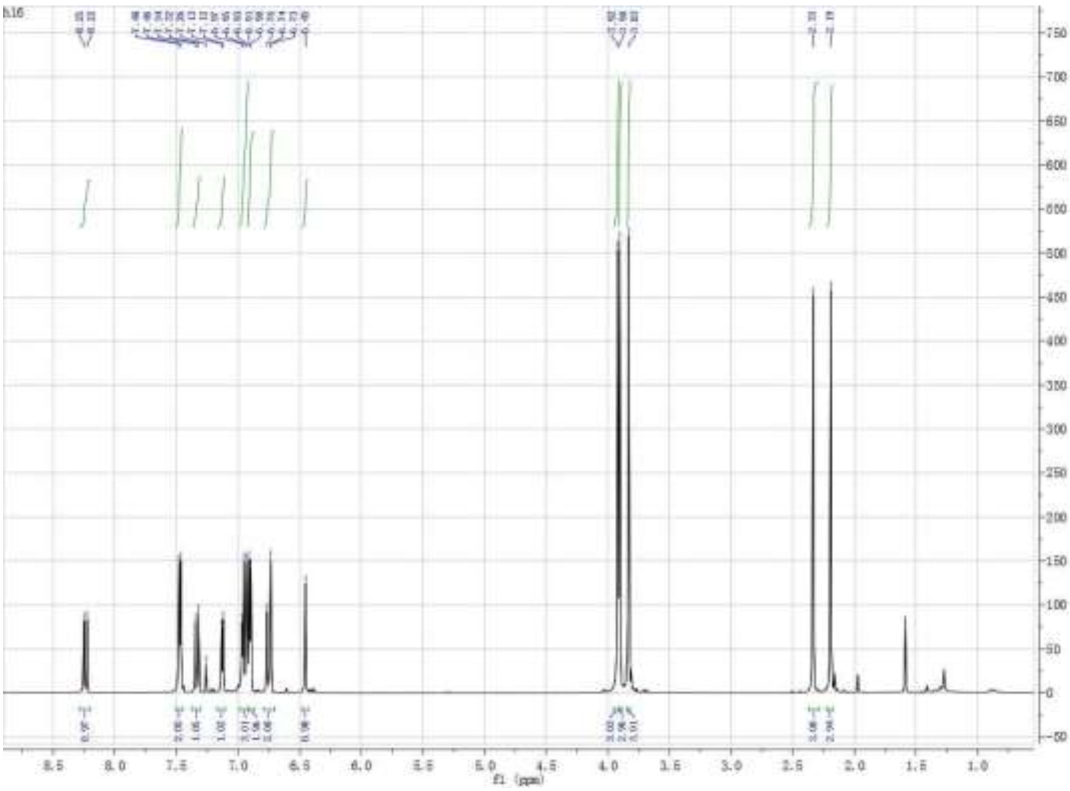

C^{13} NMR

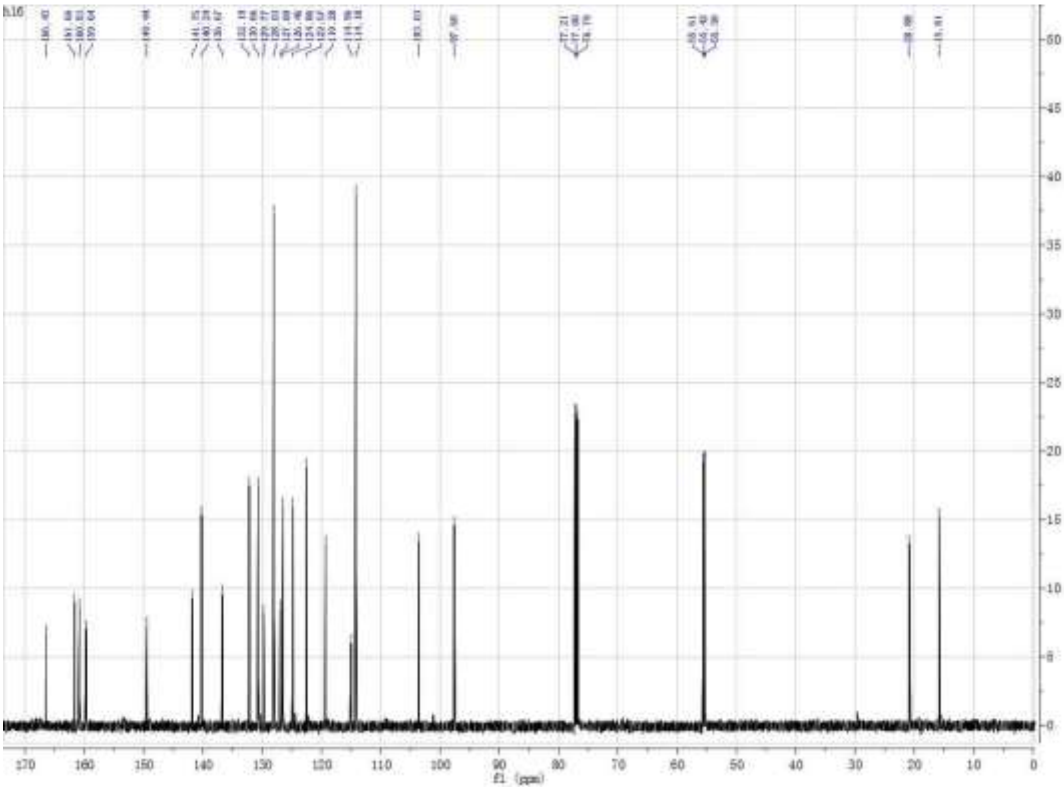

MS (EI): 445.2. ($C_{28}H_{28}O_5$, $[M+H]^+$).

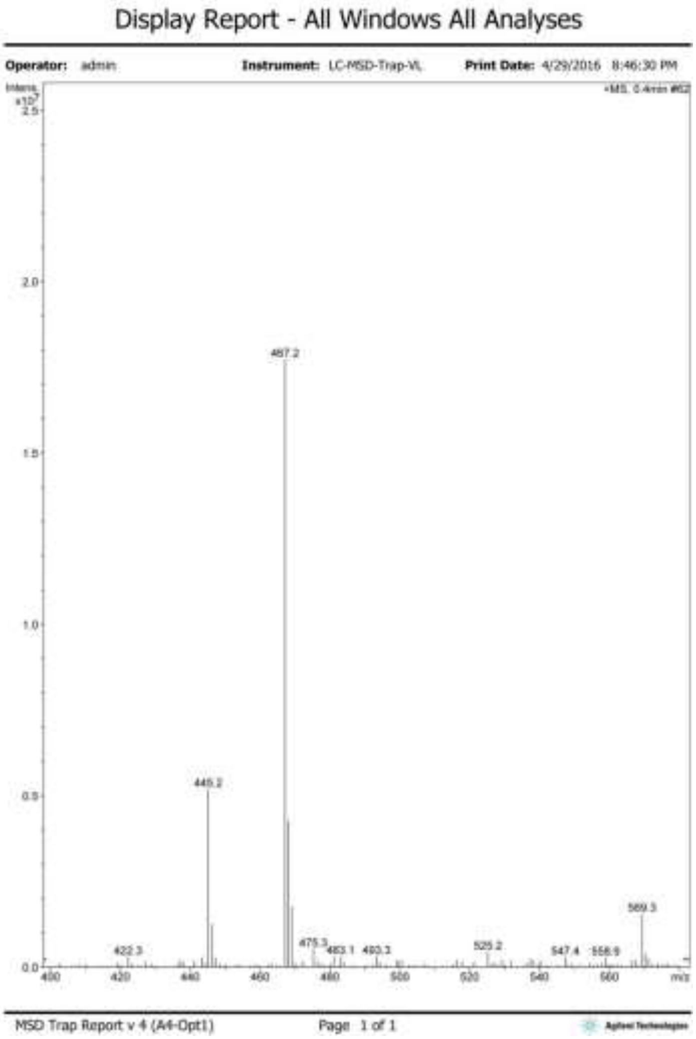
Display Report - All Windows All Analyses
Operator: admin
Instrument: LC-MSD-Trap-VL
Print Date: 4/29/2016 8:46:30 PM
487.2
445.2
569.3
422.3
475.3
483.1
493.1
525.2
547.4
558.9
MSD Trap Report v 4 (A4-Opt1)
Page 1 of 1

D6:

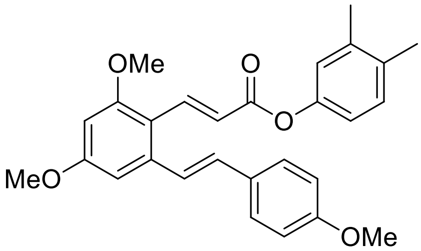
OMe
O
O
MeO
OMe

H[1] NMR

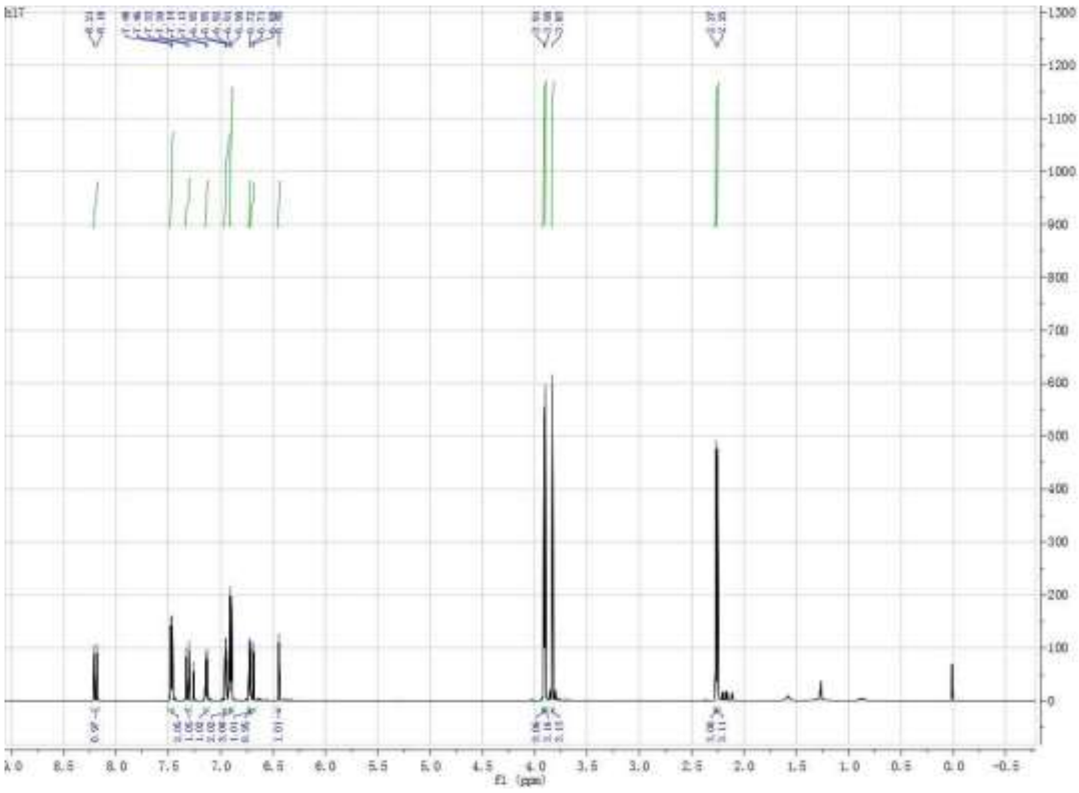

C^{13} NMR

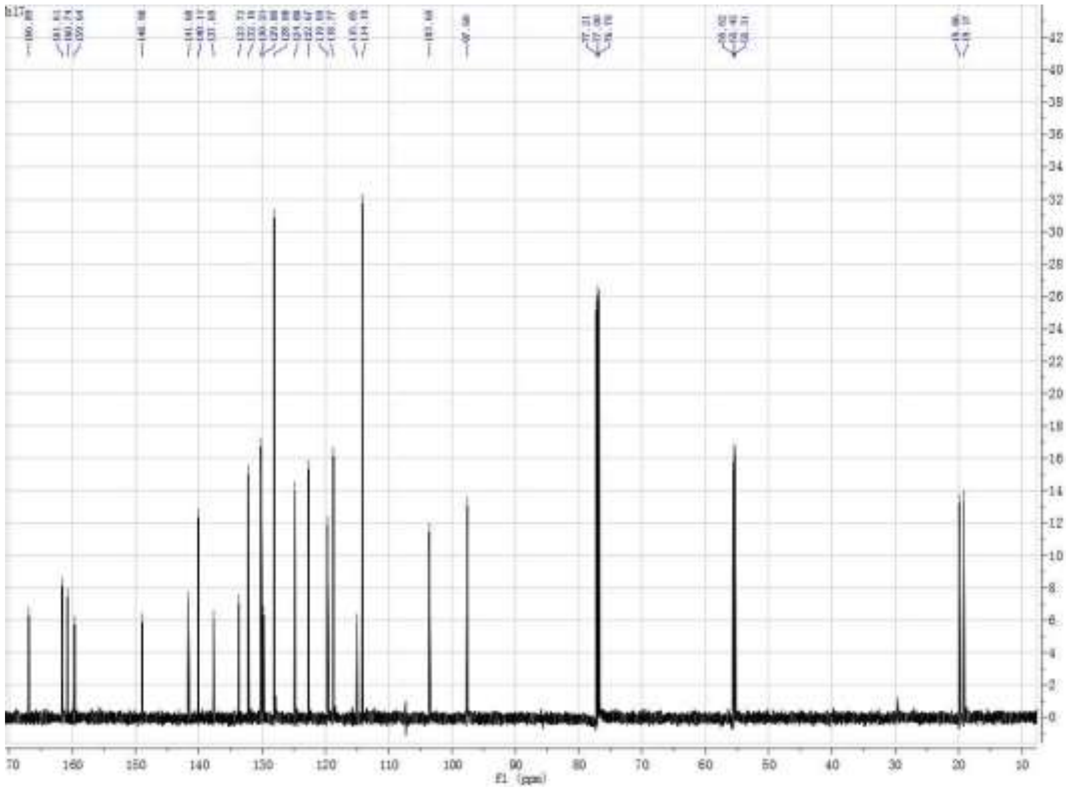

MS (EI): 445.2. ($C_{28}H_{28}O_5$, $[M+H]^+$).

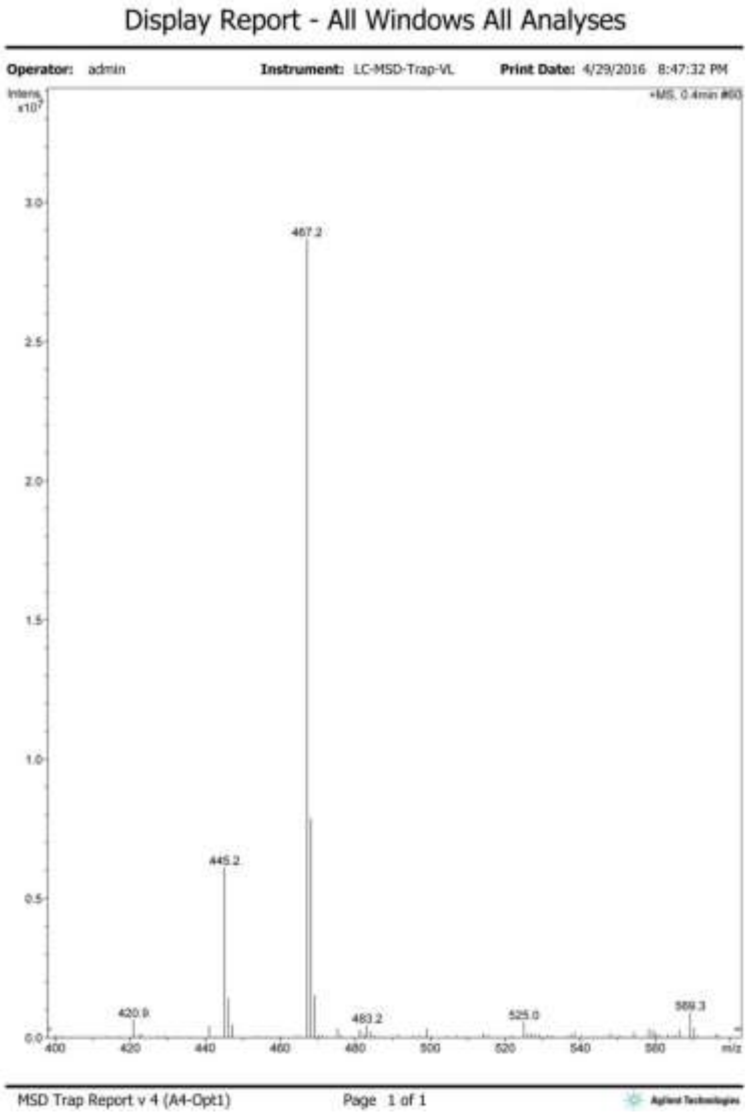
Display Report - All Windows All Analyses
Operator: admin
Instrument: LC-MSD-Trap-VL
Print Date: 4/29/2016 8:47:32 PM
Intens. x10^7
+MS, 0.4min #60
467.2
445.2
420.9
483.2
525.0
569.3
m/z
MSD Trap Report v 4 (A4-Opt1)
Page 1 of 1
Agilent Technologies

D7:

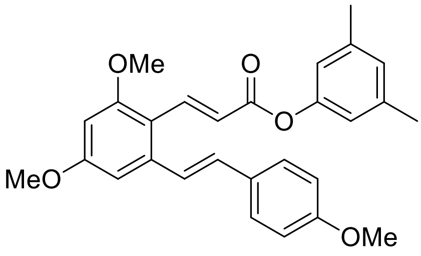
OMe
O
O
MeO
OMe

H[1] NMR

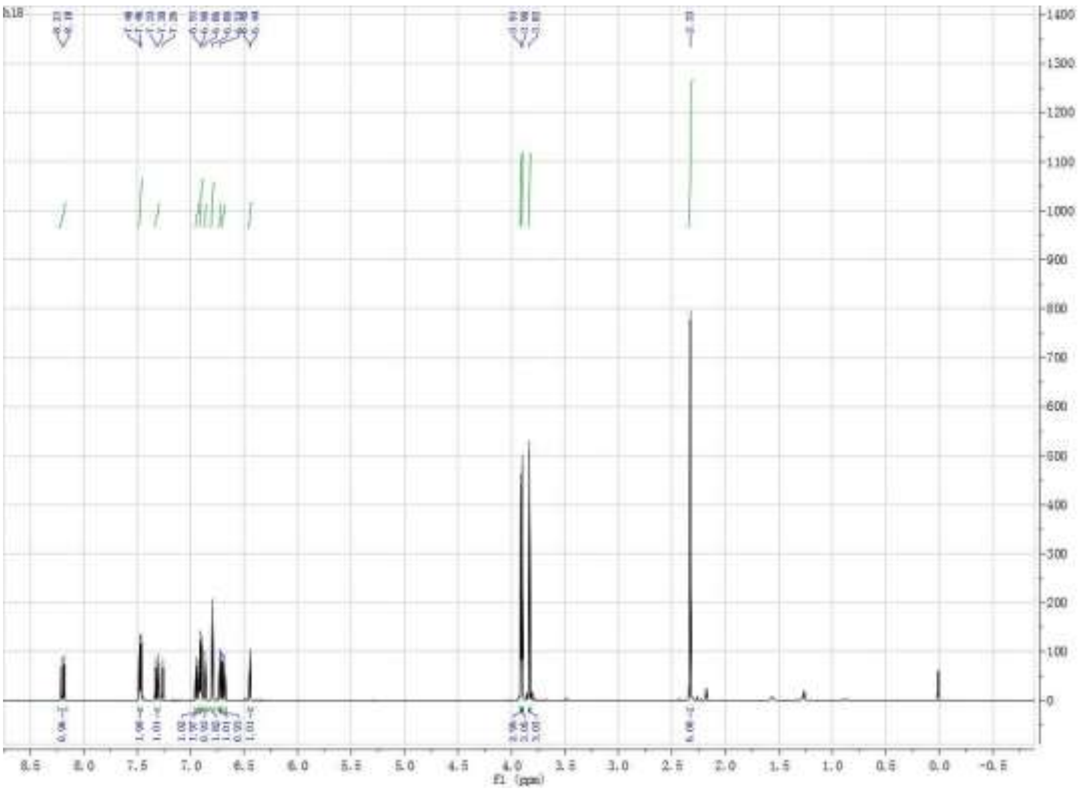

C[13] NMR

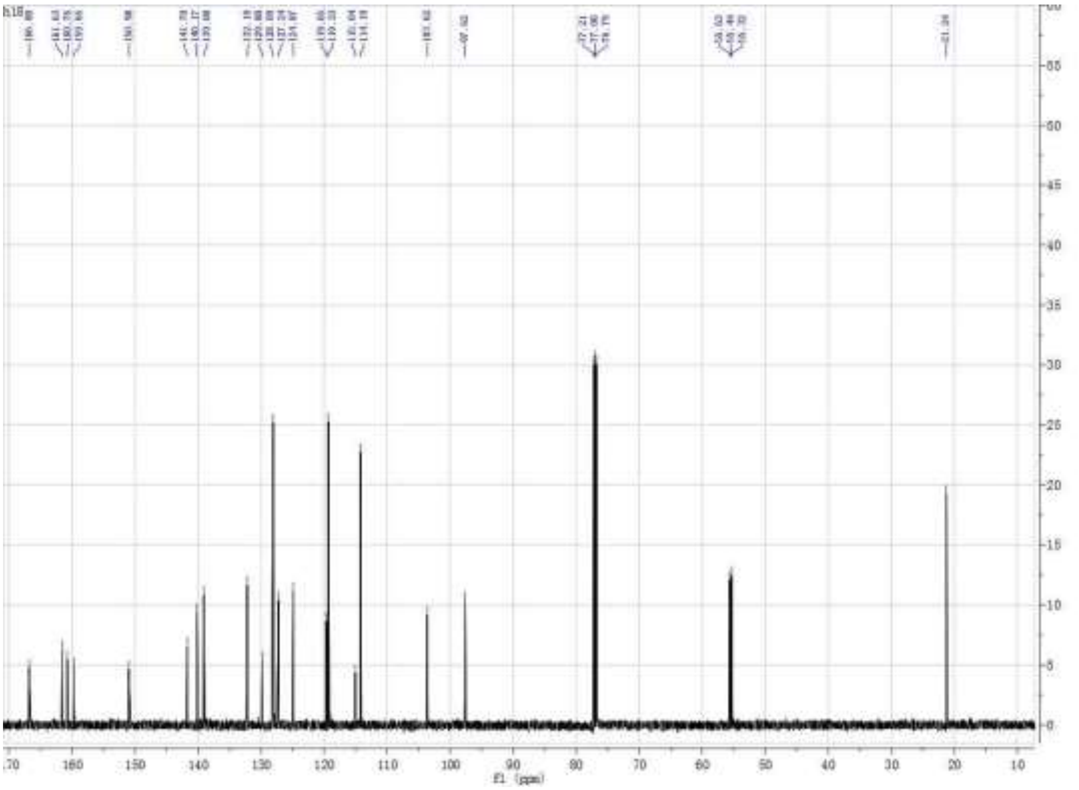

MS (EI): 445.2. ($C_{28}H_{28}O_5$, $[M+H]^+$).

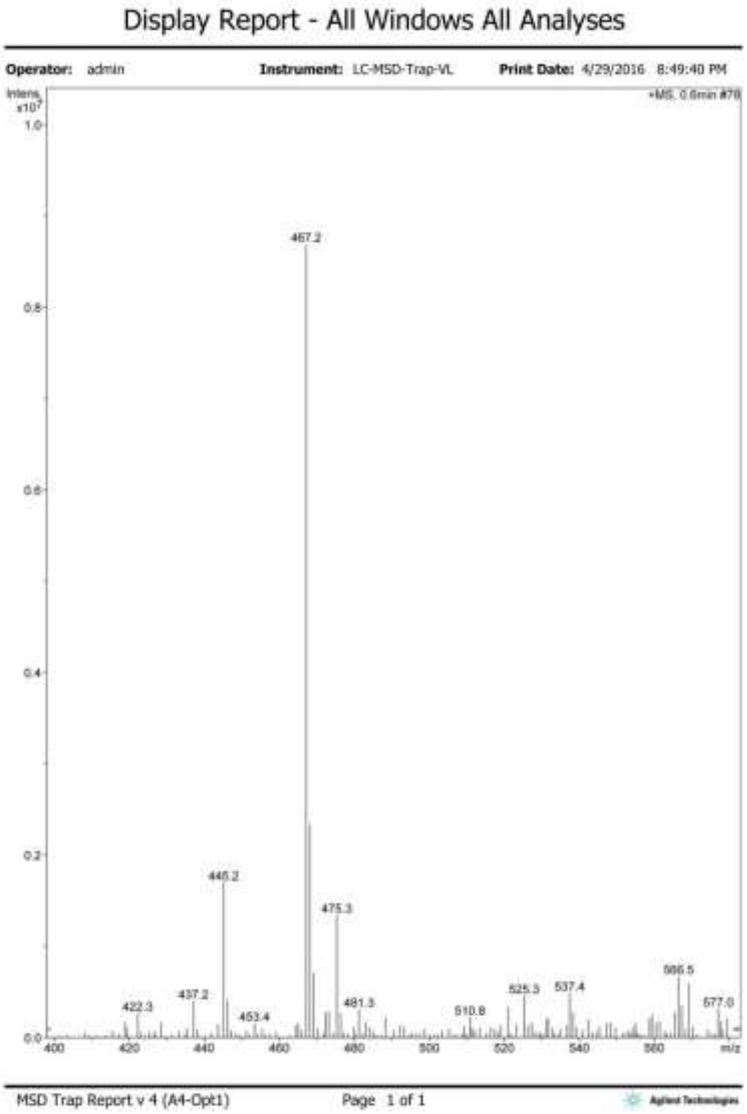
Display Report - All Windows All Analyses
Operator: admin
Instrument: LC-MSD-Trap-VL
Print Date: 4/29/2016 8:49:40 PM
Intens. x10^7
467.2
445.2
475.3
422.3
437.2
453.4
481.3
510.8
525.3
537.4
566.5
577.0
m/z
MSD Trap Report v 4 (A4-Opt1)
Page 1 of 1

D8:

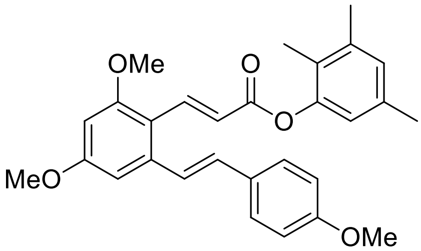
OMe
O
O
MeO
OMe

H1 NMR

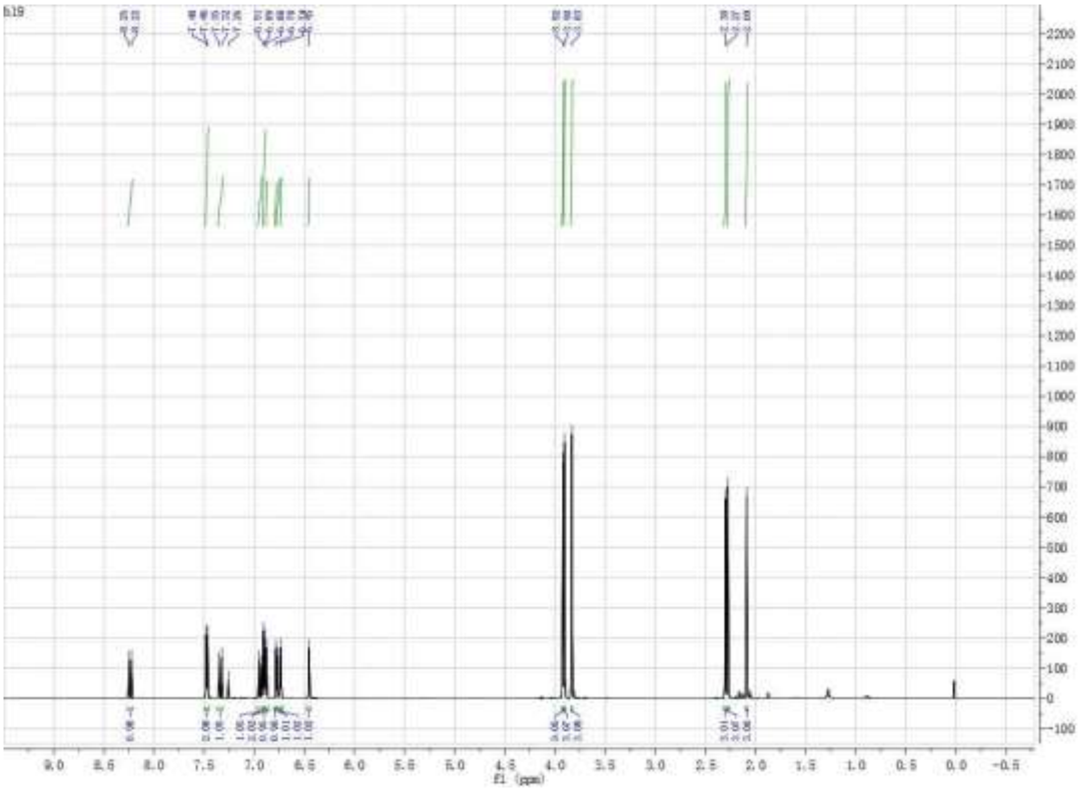

C13 NMR

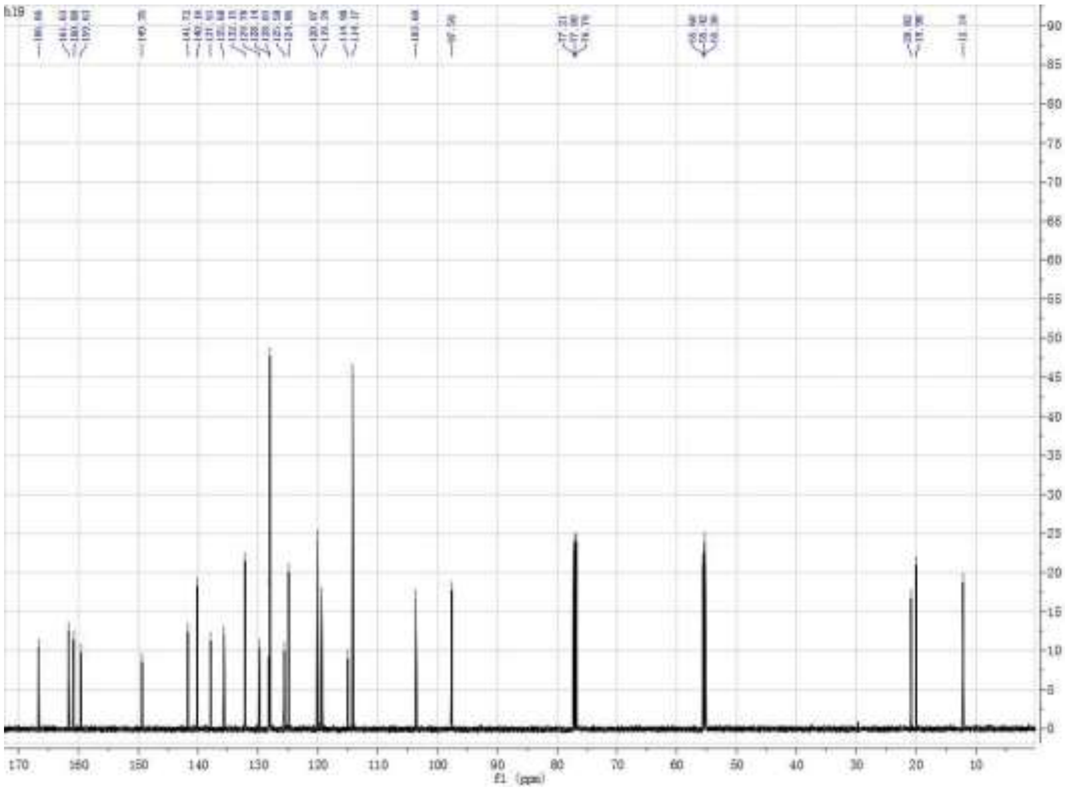

MS (EI): 459.2. ($C_{29}H_{30}O_5$, $[M+H]^+$).

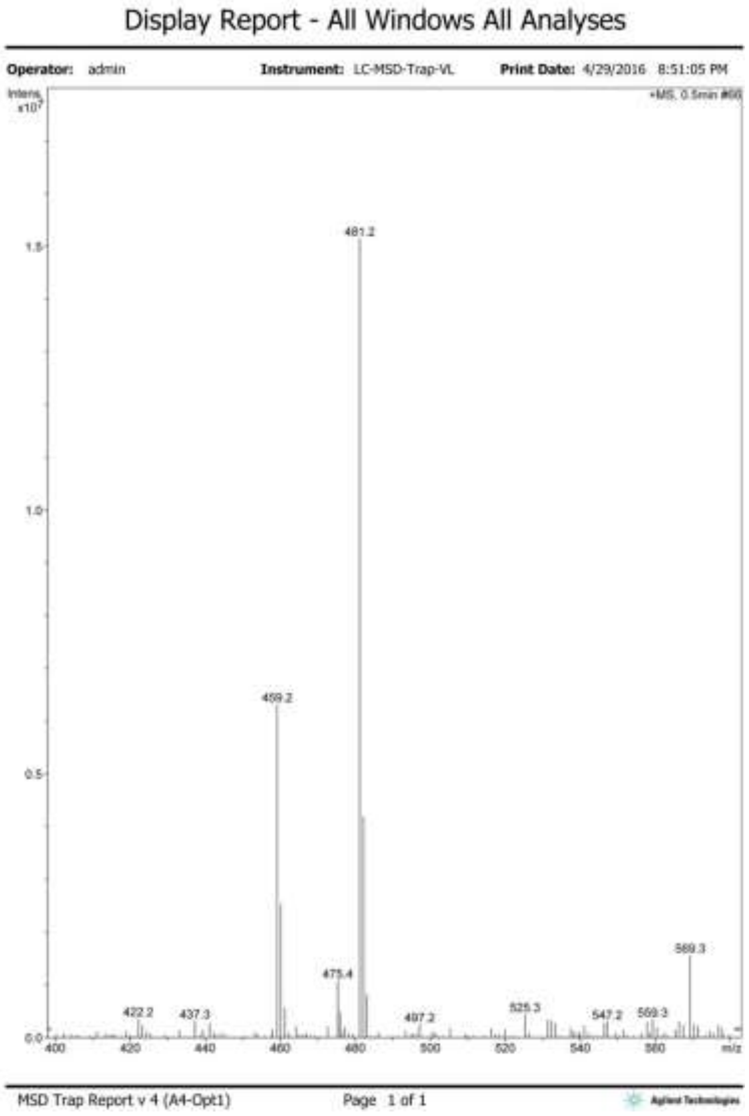

Display Report - All Windows All Analyses
Operator: admin
Instrument: LC-MSD-Trap-VL
Print Date: 4/29/2016 8:51:05 PM
481.2
459.2
475.4
569.3
422.2
437.3
497.2
525.3
547.2
MSD Trap Report v 4 (A4-Opt1)
Page 1 of 1

D9:

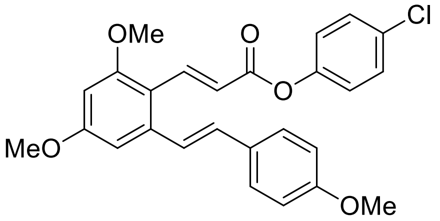

OMe
O
Cl
O
MeO
OMe

H^1 NMR

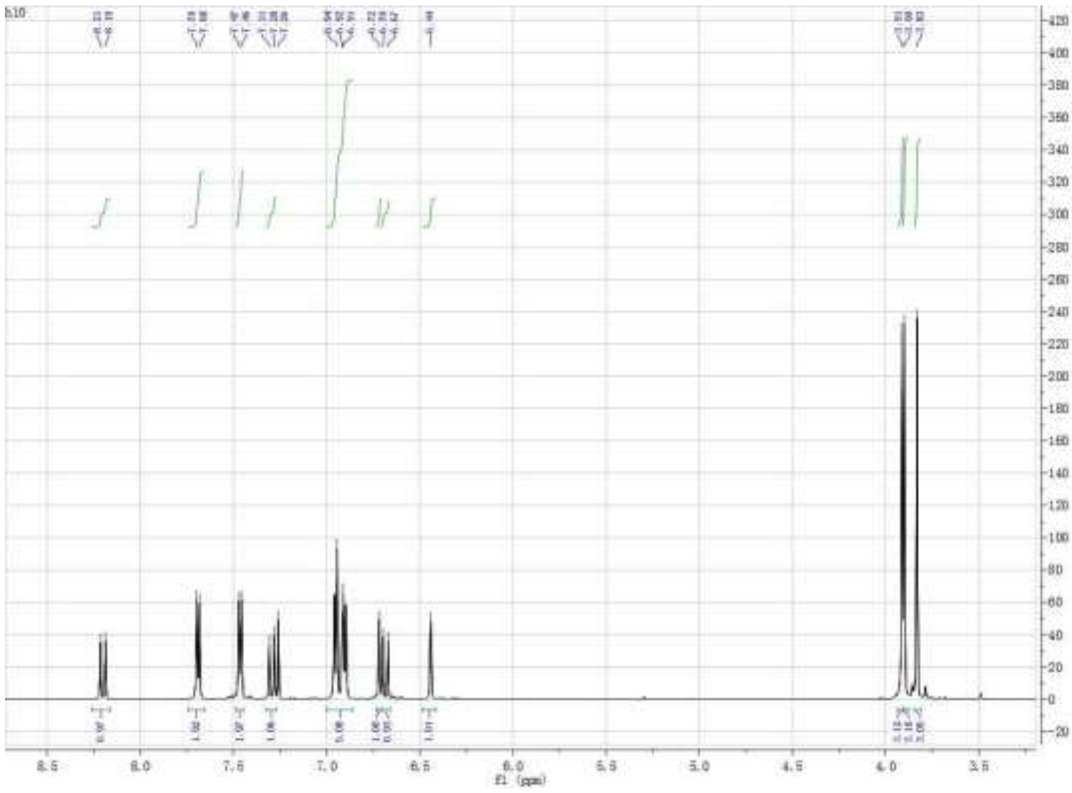

C^{13} NMR

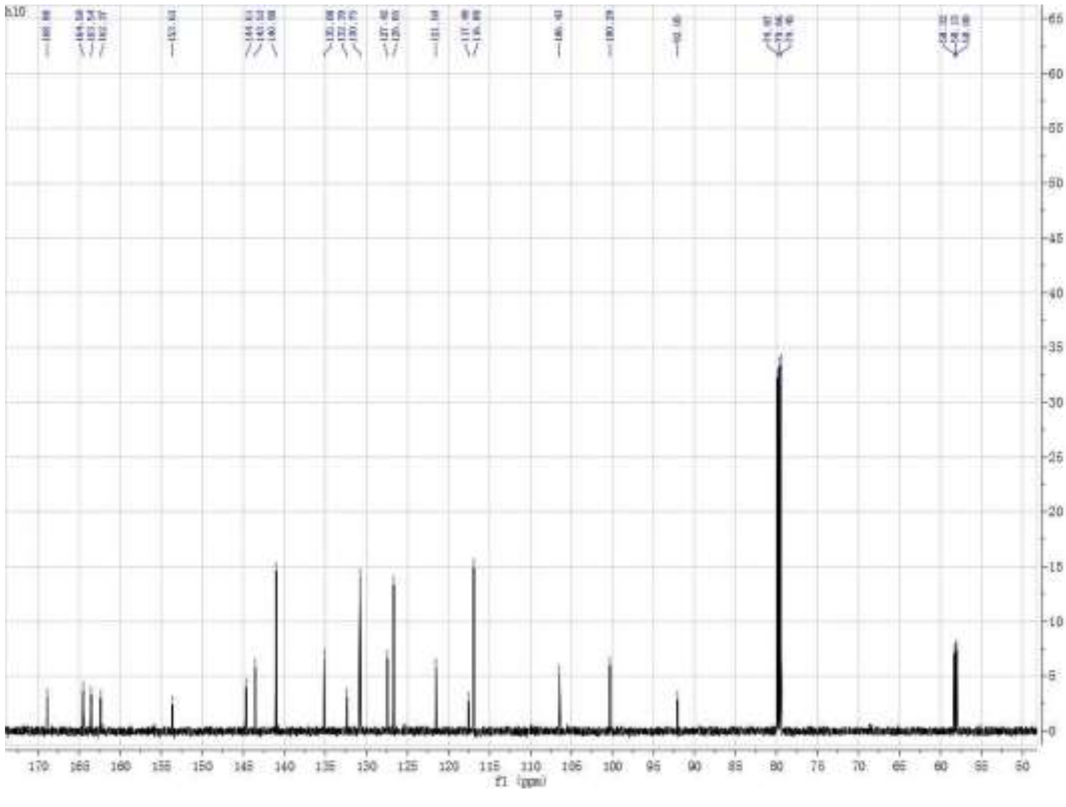

MS (EI): 451.1 ($C_{26}H_{23}ClO_5$, $[M+H]^+$).

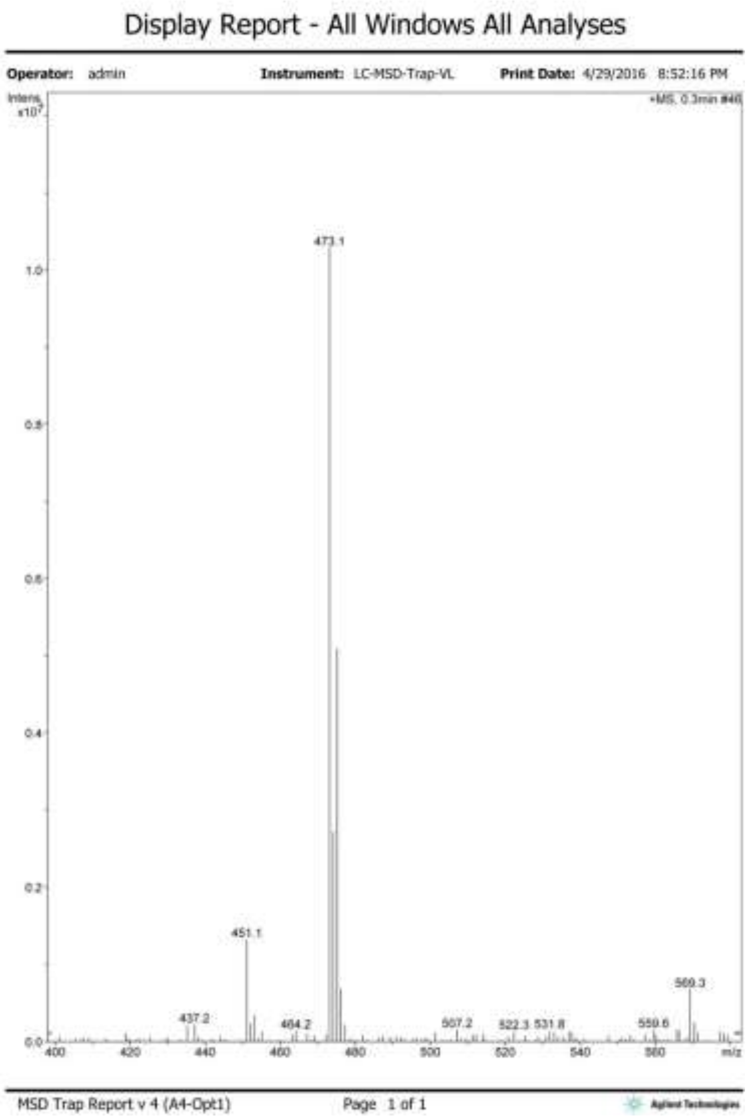
Display Report - All Windows All Analyses
Operator: admin
Instrument: LC-MSD-Trap-VL
Print Date: 4/29/2016 8:52:16 PM
+MS, 0.3min #46
473.1
451.1
437.2
464.2
507.2
522.3
531.8
559.6
569.3
m/z
MSD Trap Report v 4 (A4-Opt1)
Page 1 of 1

D10:

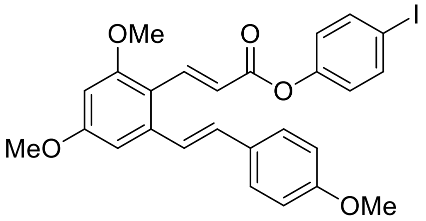
OMe
O
I
O
MeO
OMe

H^1 NMR

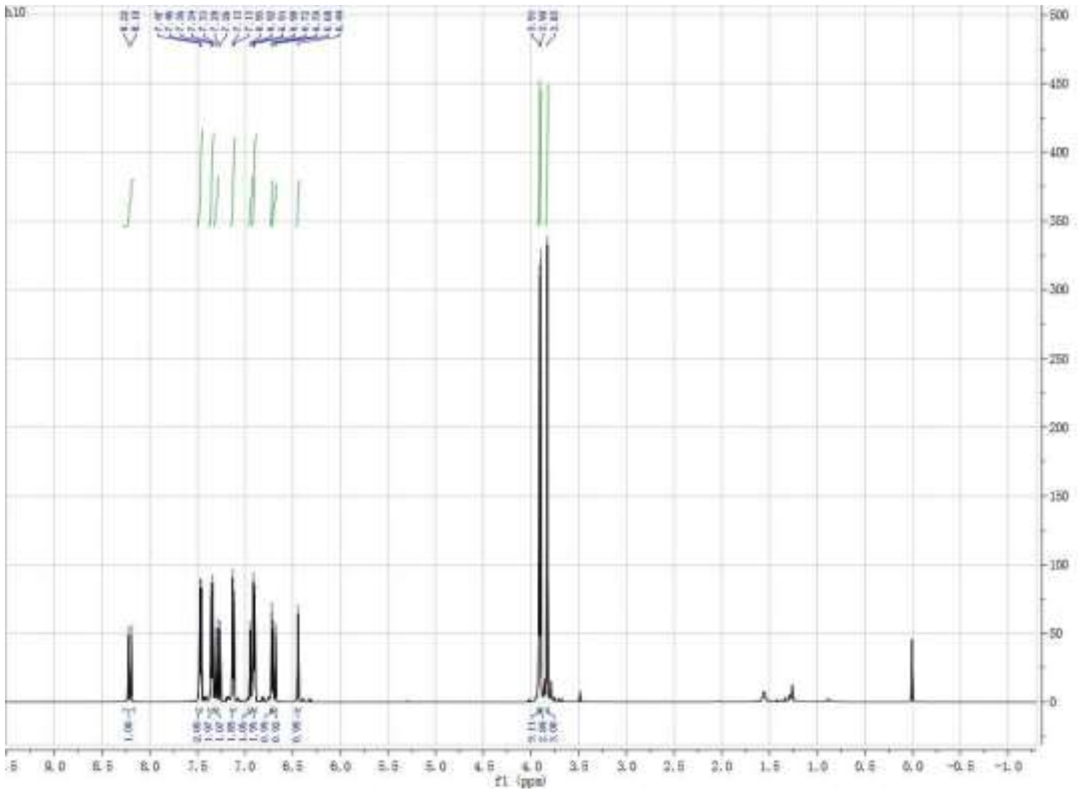

C^{13} NMR

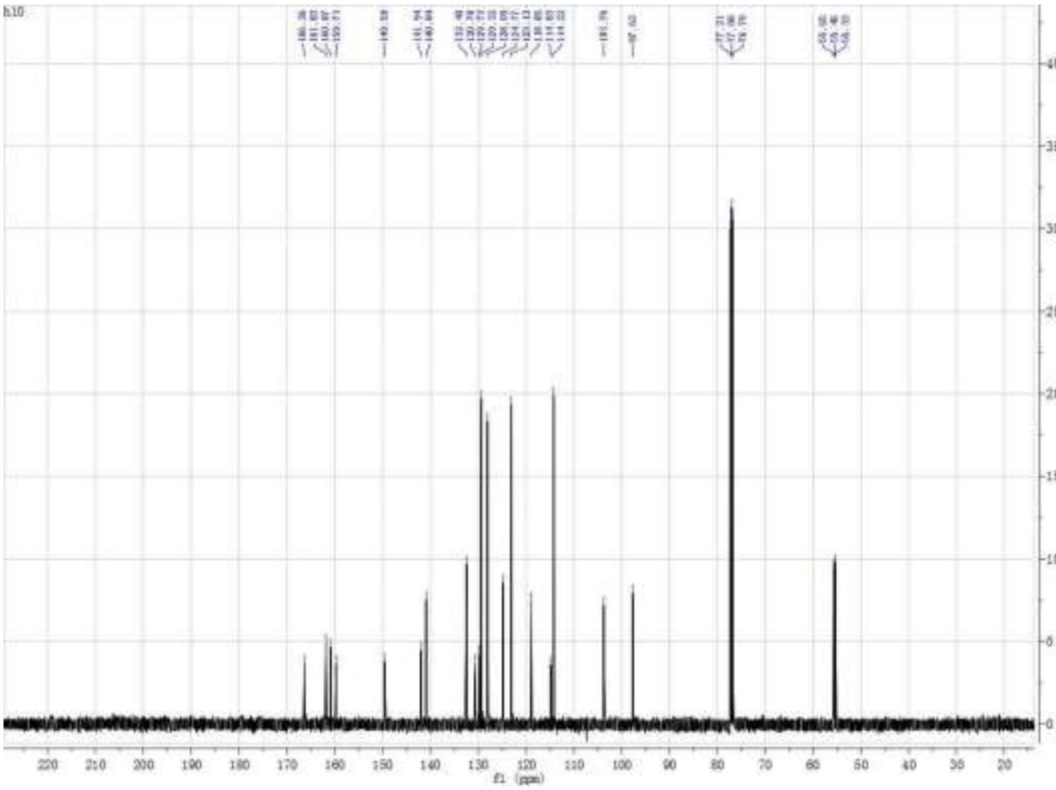

MS (EI): 543.1 ($C_{26}H_{23}IO_5$, $[M+H]^+$).

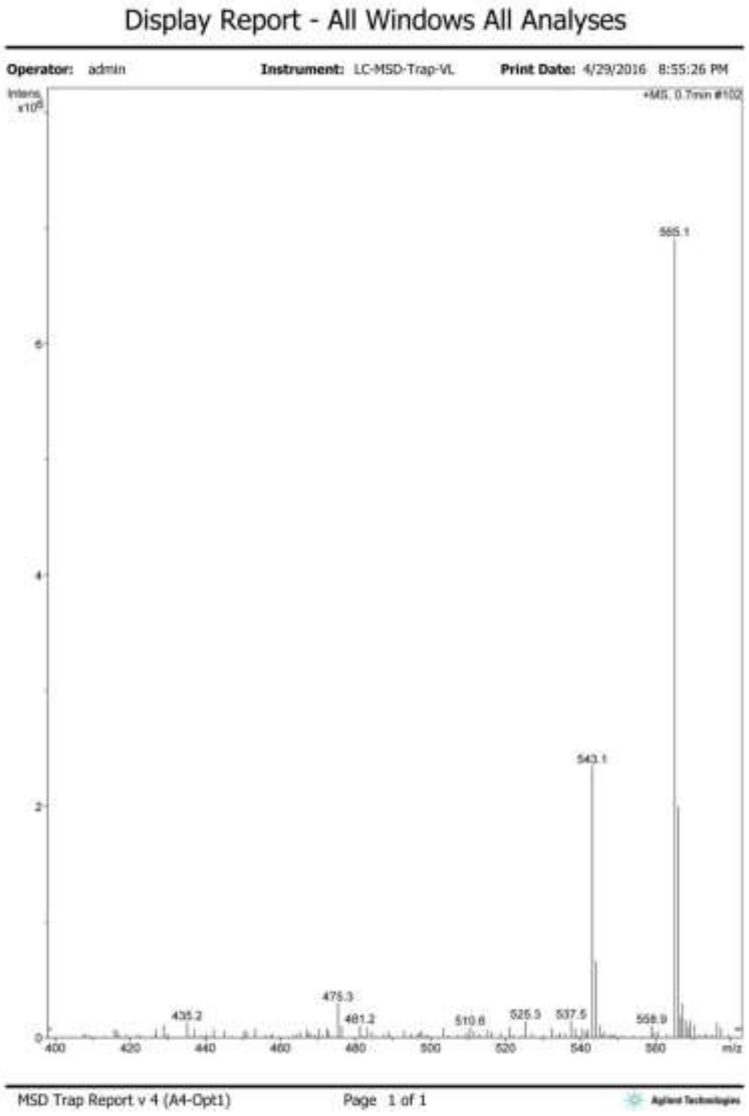
Display Report - All Windows All Analyses
Operator: admin
Instrument: LC-MSD-Trap-VL
Print Date: 4/29/2016 8:55:26 PM
565.1
543.1
475.3
435.2
481.2
510.6
525.5
537.5
558.9
m/z
MSD Trap Report v 4 (A4-Opt1)
Page 1 of 1

D11:

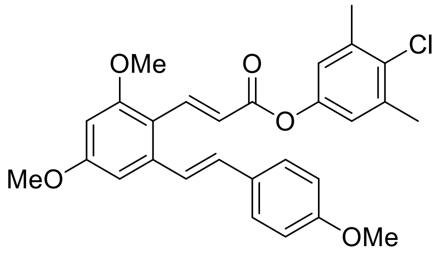
OMe
O
Cl
O
MeO
OMe

H^1 NMR

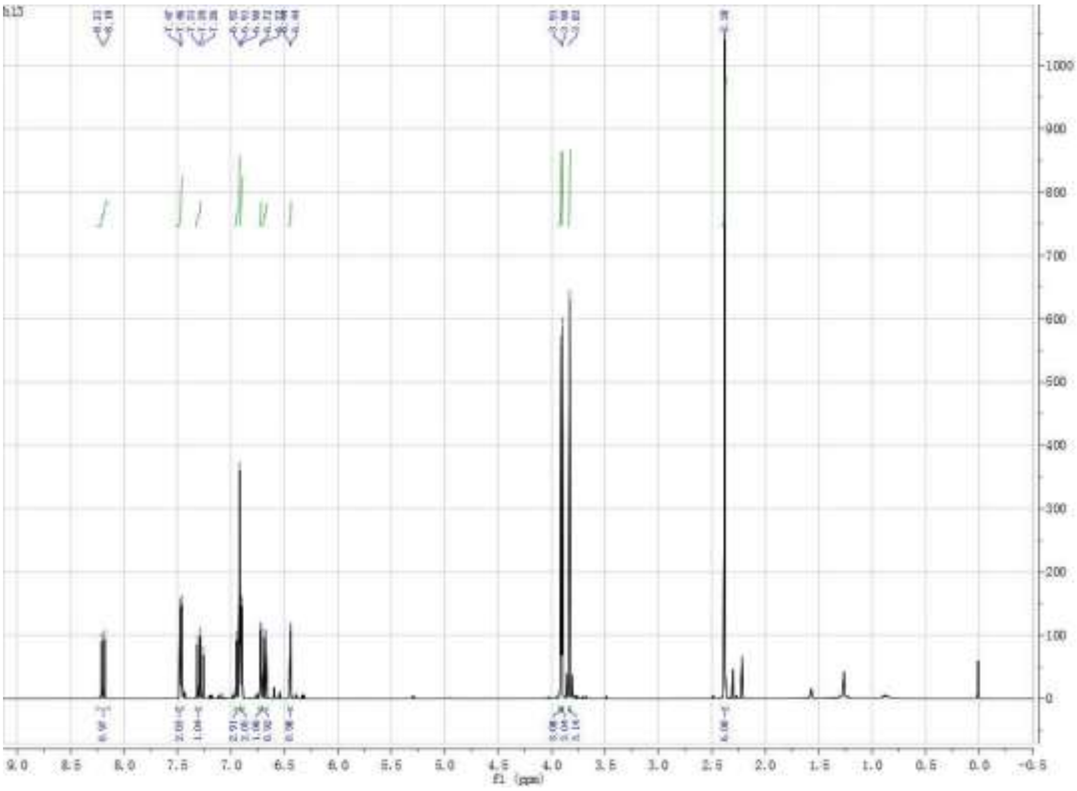

C[13] NMR

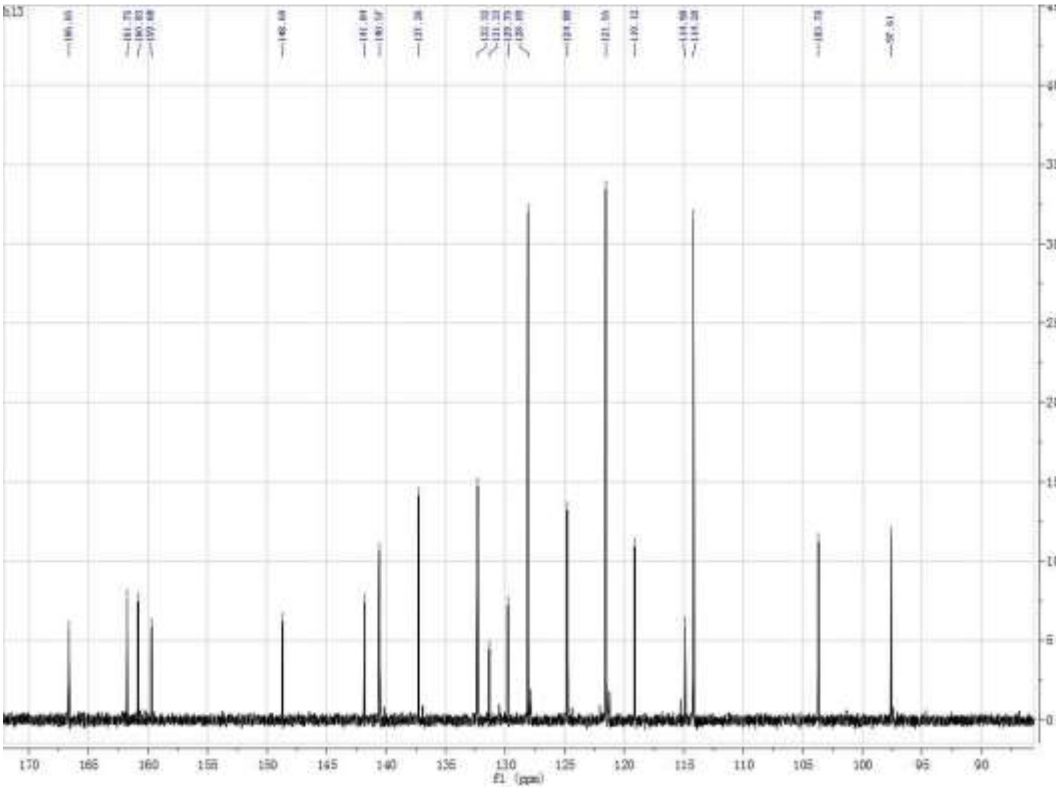

MS (EI): 479.1 ($C_{28}H_{27}ClO_5$, $[M+H]^+$).

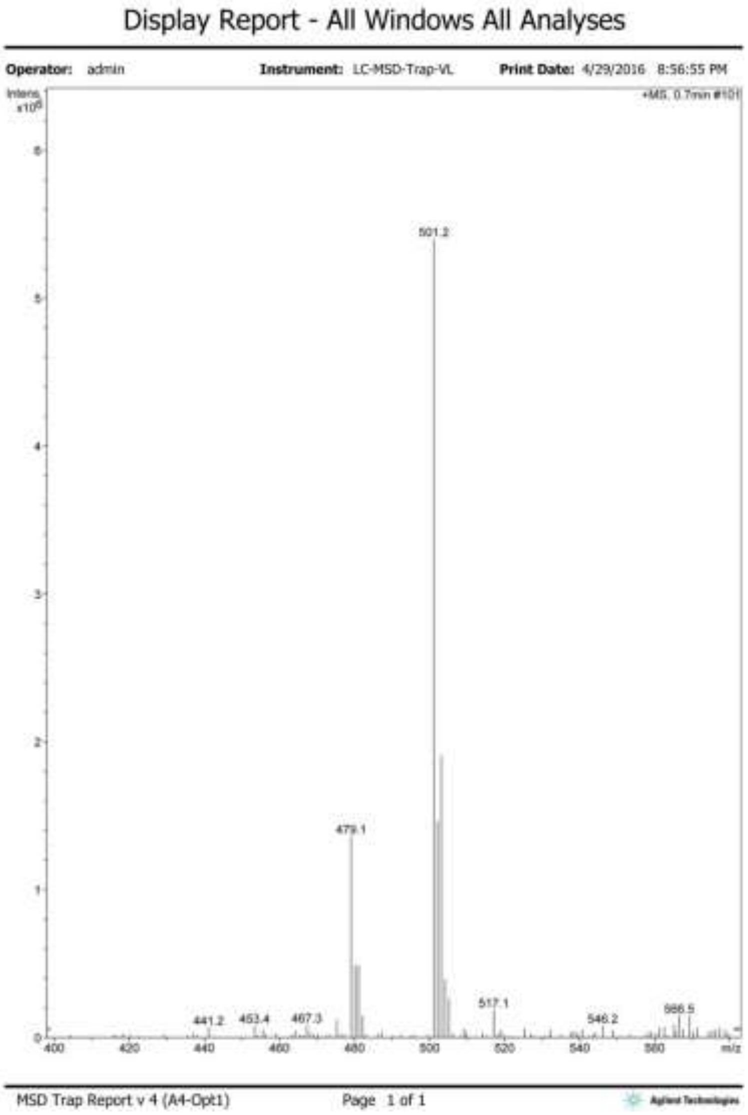
Display Report - All Windows All Analyses
Operator: admin
Instrument: LC-MSD-Trap-VL
Print Date: 4/29/2016 8:56:55 PM
+MS, 0.7min #101
501.2
479.1
441.2
453.4
467.3
517.1
546.2
566.5
m/z
MSD Trap Report v 4 (A4-Opt1)
Page 1 of 1

D12:

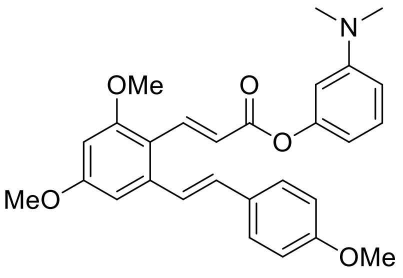
N
OMe
O
O
MeO
OMe

H[1] NMR

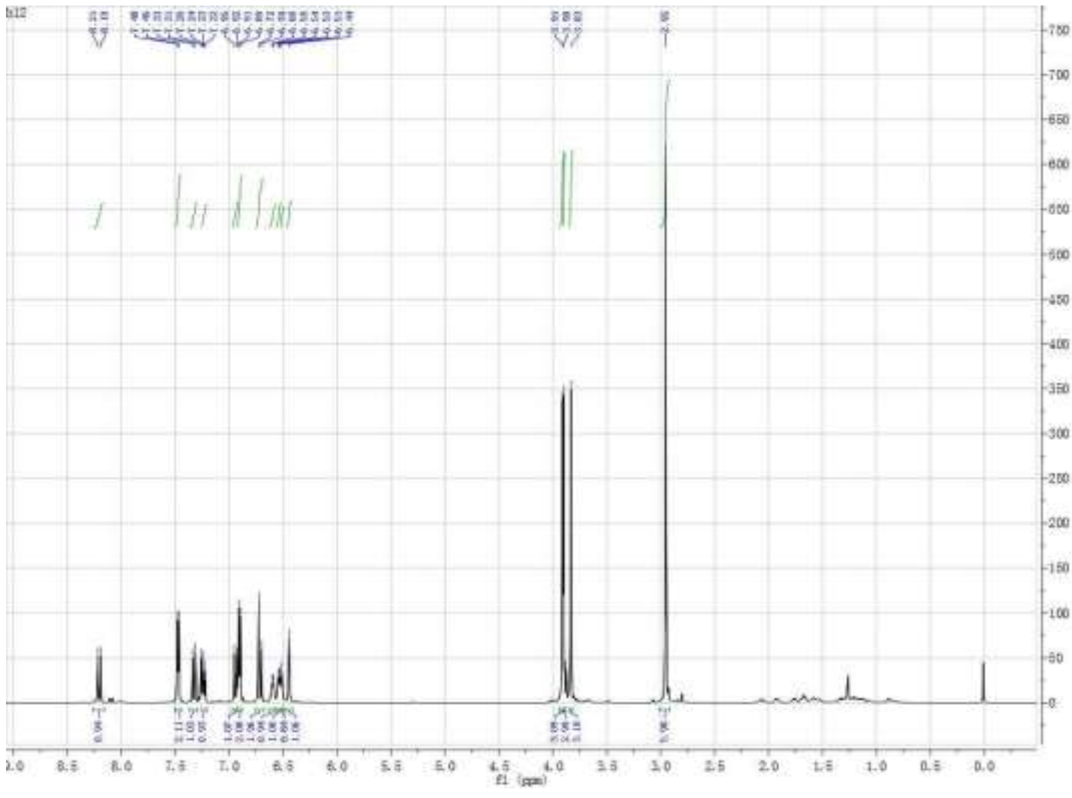

C^{13} NMR

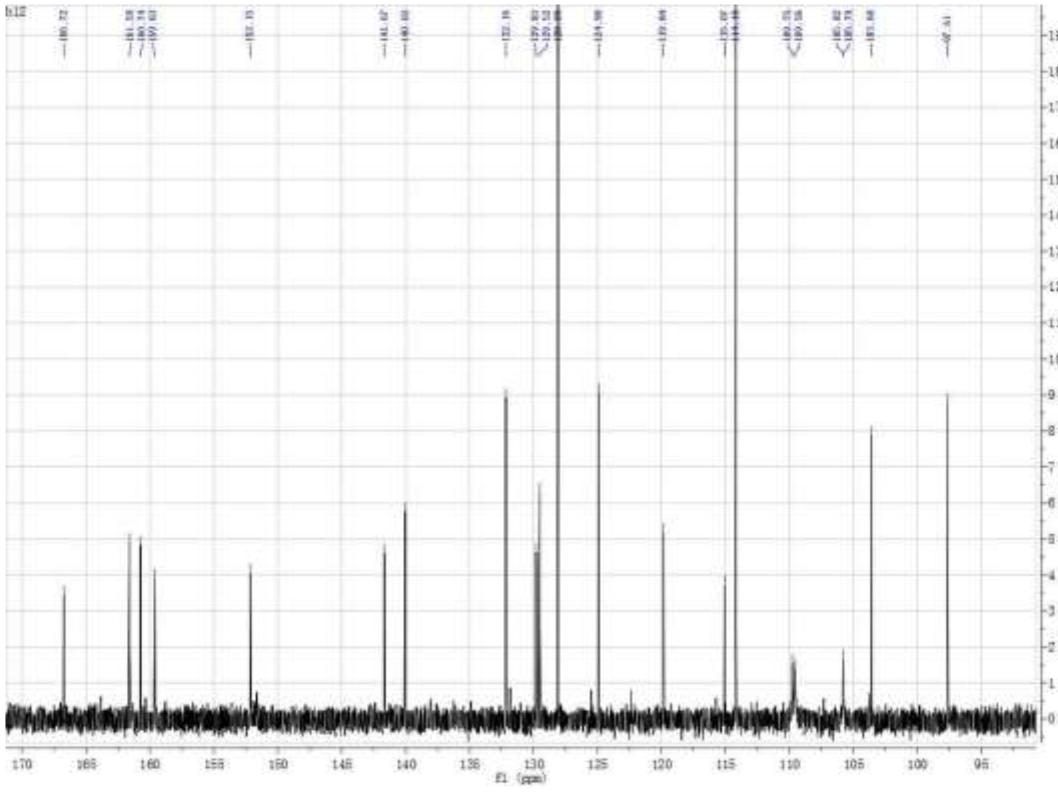

MS (EI): 460.2 ($C_{28}H_{29}NO_5$, $[M+H]^+$).

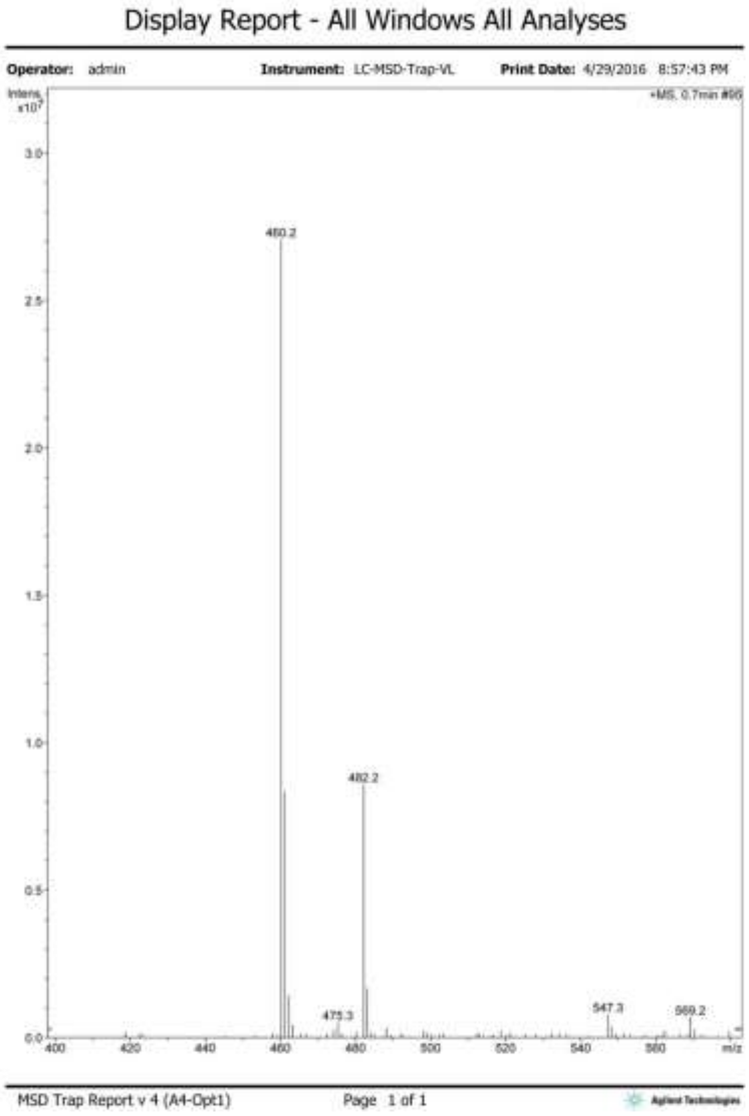
Display Report - All Windows All Analyses
Operator: admin
Instrument: LC-MSD-Trap-VL
Print Date: 4/29/2016 8:57:43 PM
Intens. x10^7
+MS, 0.7min #96
460.2
482.2
475.3
547.3
569.2
m/z
MSD Trap Report v 4 (A4-Opt1)
Page 1 of 1
Agilent Technologies

D13:

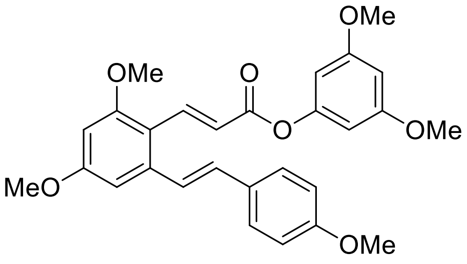
OMe
OMe
O
O
OMe
MeO
OMe

H[1] NMR

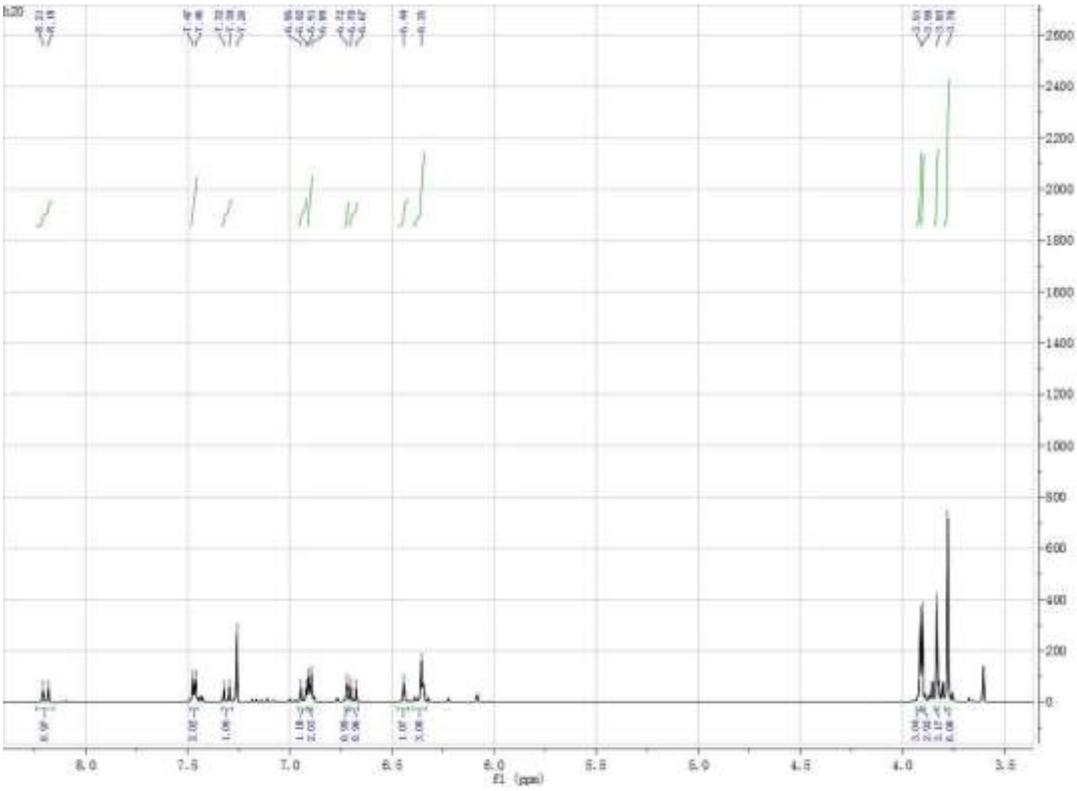

C^{13} NMR

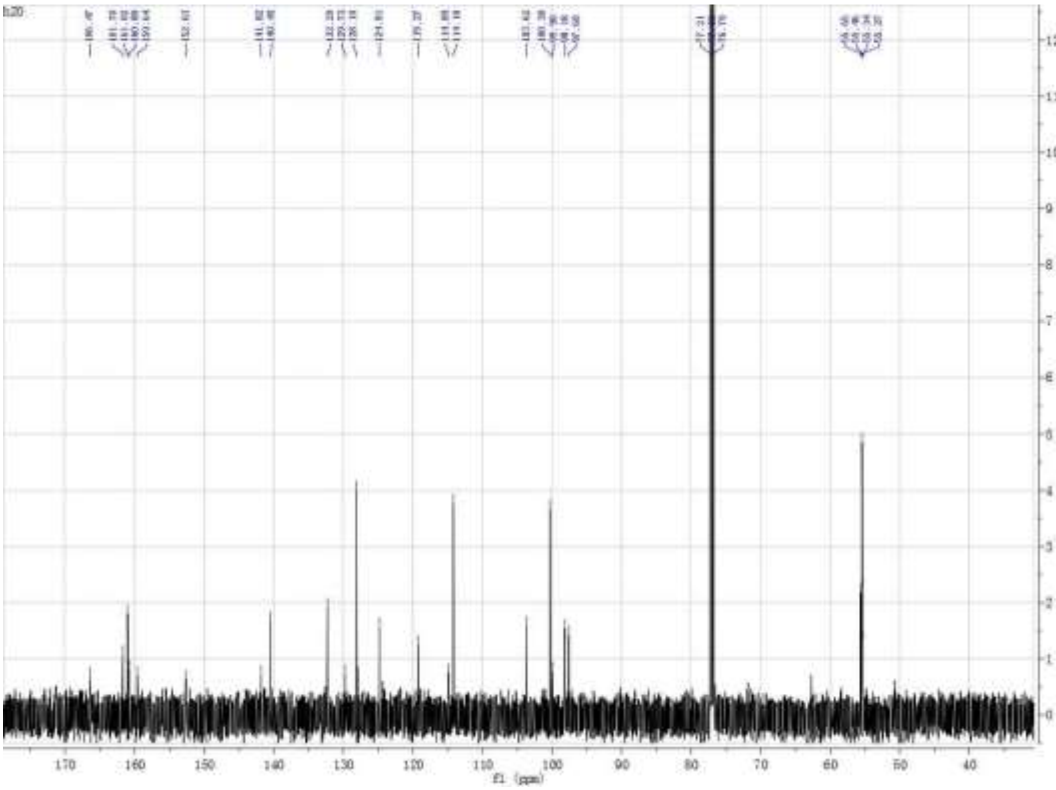

MS (EI): 477.2 ($C_{28}H_{28}O_7$, $[M+H]^+$).

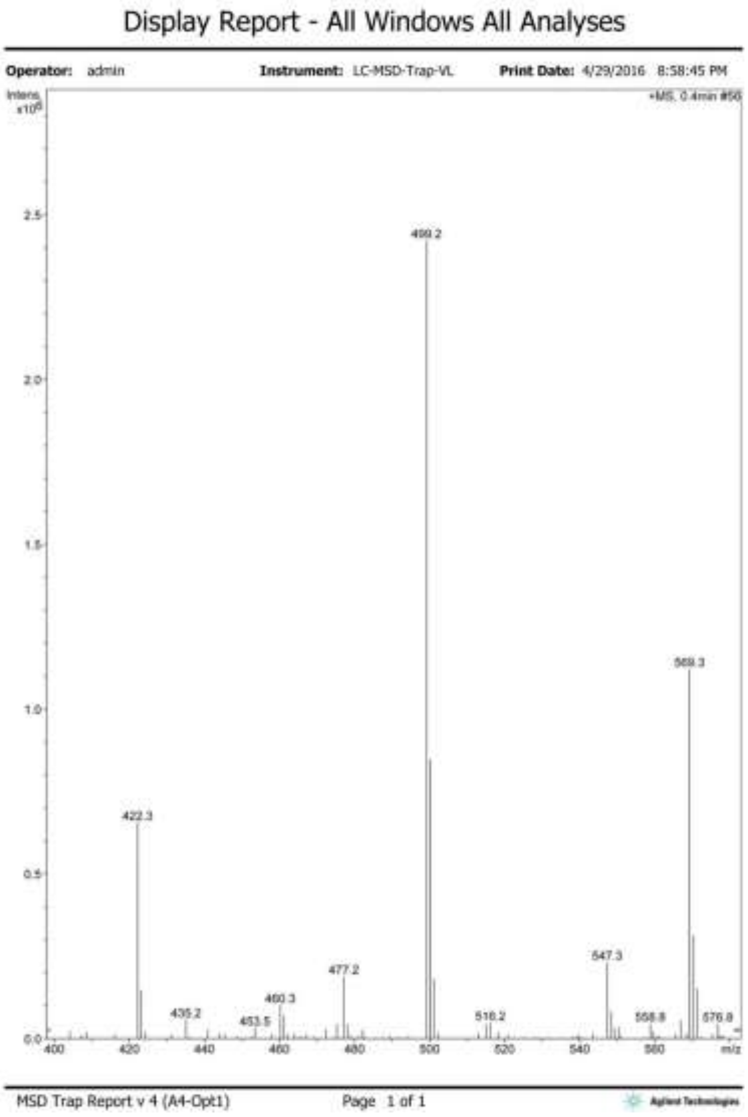
Display Report - All Windows All Analyses
Operator: admin
Instrument: LC-MSD-Trap-VL
Print Date: 4/29/2016 8:58:45 PM
Intens. x10^6
+MS, 0.4min #56
499.2
569.3
422.3
477.2
547.3
460.3
435.2
453.5
516.2
558.8
576.8
m/z
MSD Trap Report v 4 (A4-Opt1)
Page 1 of 1

D14:

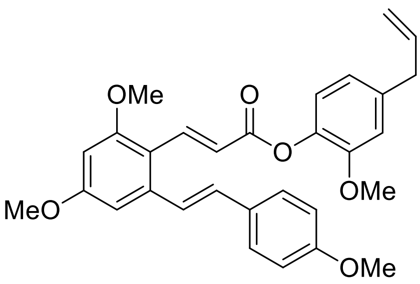
OMe
O
O
OMe
MeO
OMe

H^1 NMR

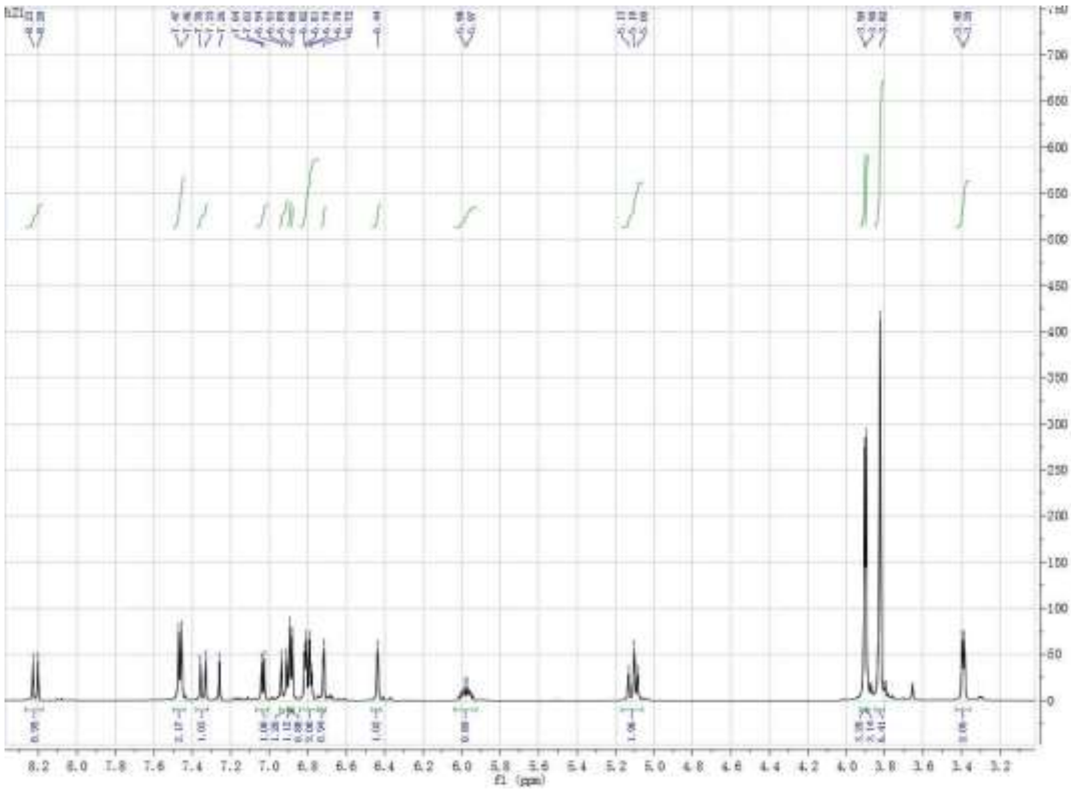

C^{13} NMR

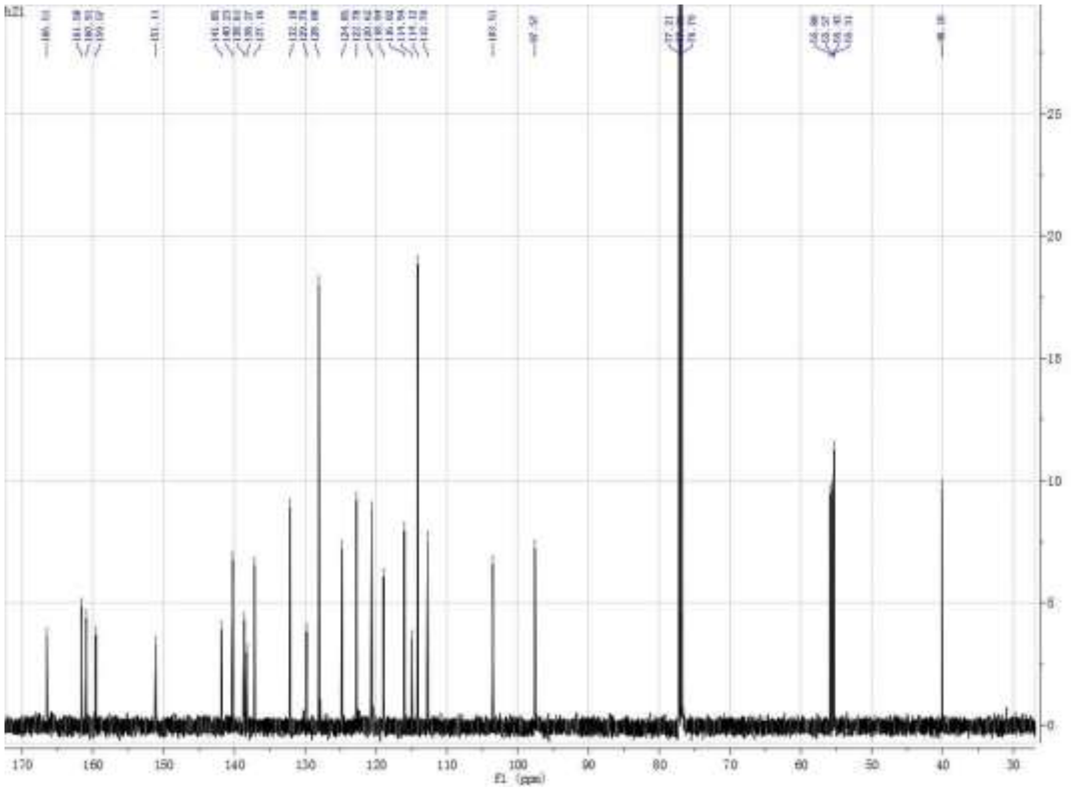

MS (EI): 487.2 ($C_{30}H_{30}O_6$, $[M+H]^+$).

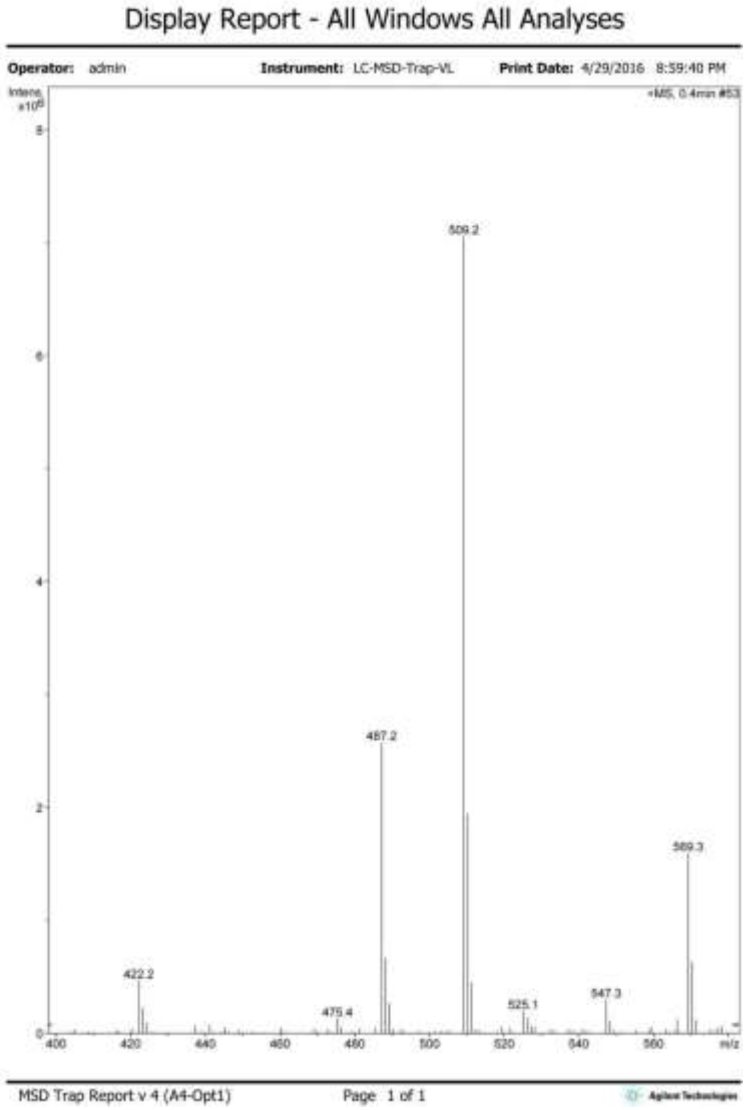
Display Report - All Windows All Analyses
Operator: admin
Instrument: LC-MSD-Trap-VL
Print Date: 4/29/2016 8:59:40 PM
+MS, 0.4min #53
Intens. x10^6
509.2
487.2
569.3
422.2
547.3
525.1
475.4
m/z
MSD Trap Report v 4 (A4-Opt1)
Page 1 of 1
Agilent Technologies

D15:

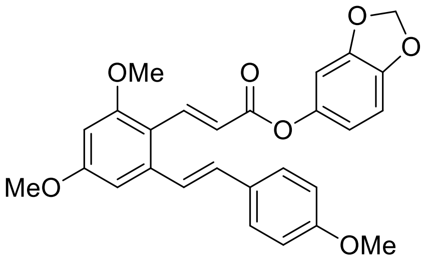
OMe
O
O
O
O
MeO
OMe

H^1 NMR

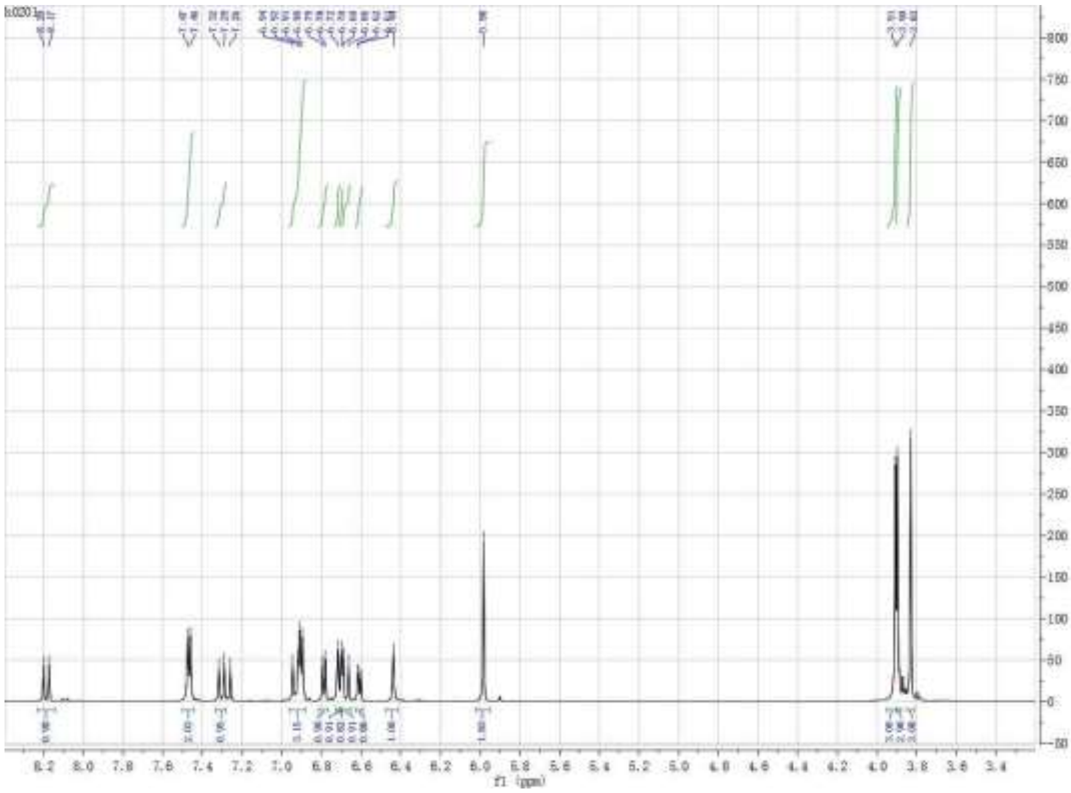

C^{13} NMR

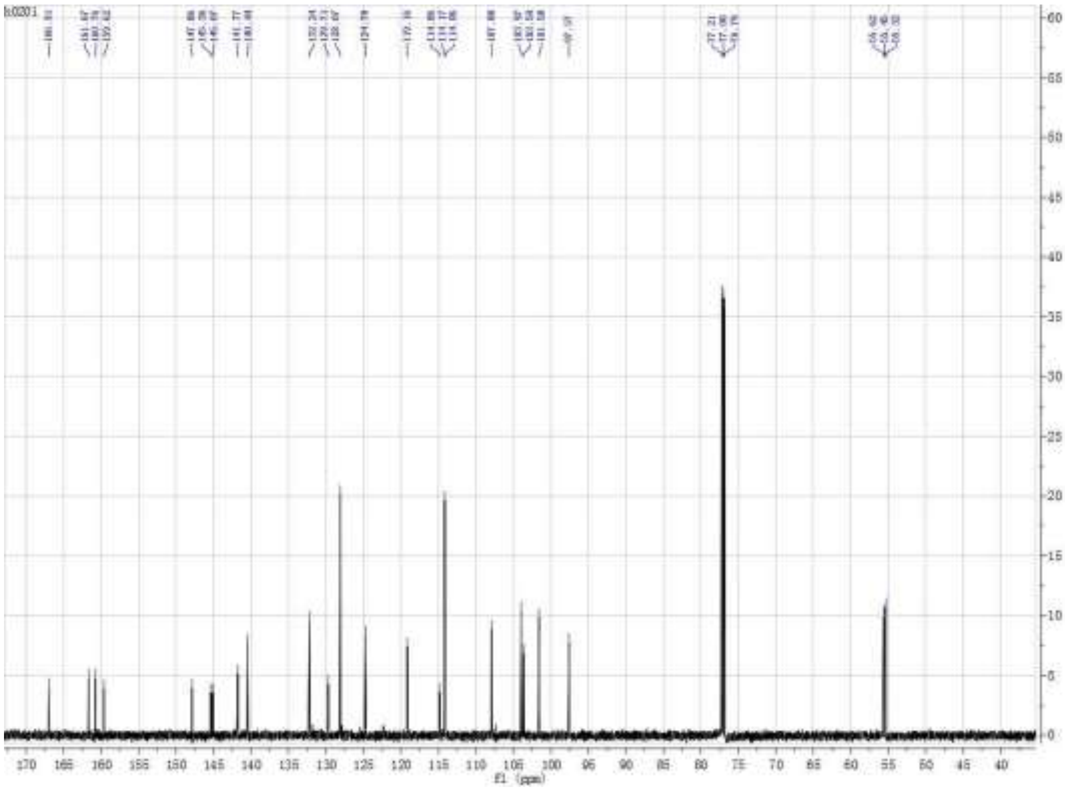

MS(EI):461.2($C_{27}H_{24}O_7$,$[M+H]^+$).

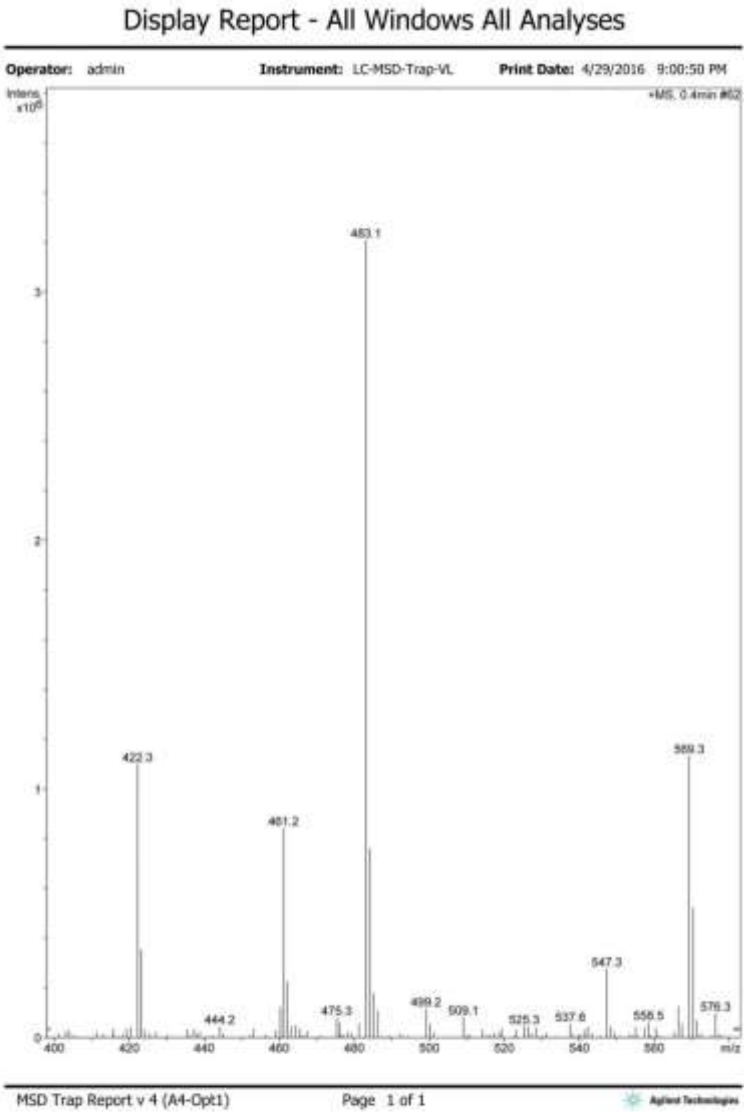

Display Report - All Windows All Analyses
Operator: admin
Instrument: LC-MSD-Trap-VL
Print Date: 4/29/2016 9:00:50 PM
+MS, 0.4min #62
483.1
422.3
461.2
569.3
547.3
444.2
475.3
499.2
509.1
525.3
537.6
556.5
576.3
m/z
MSD Trap Report v 4 (A4-Opt1)
Page 1 of 1

D16:

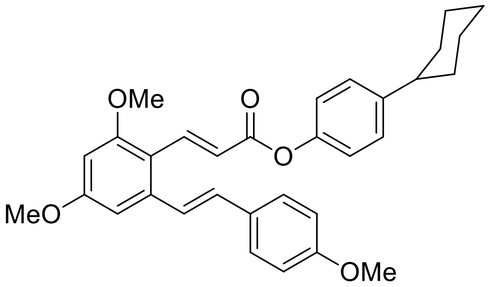

OMe
O
O
MeO
OMe

H[1] NMR

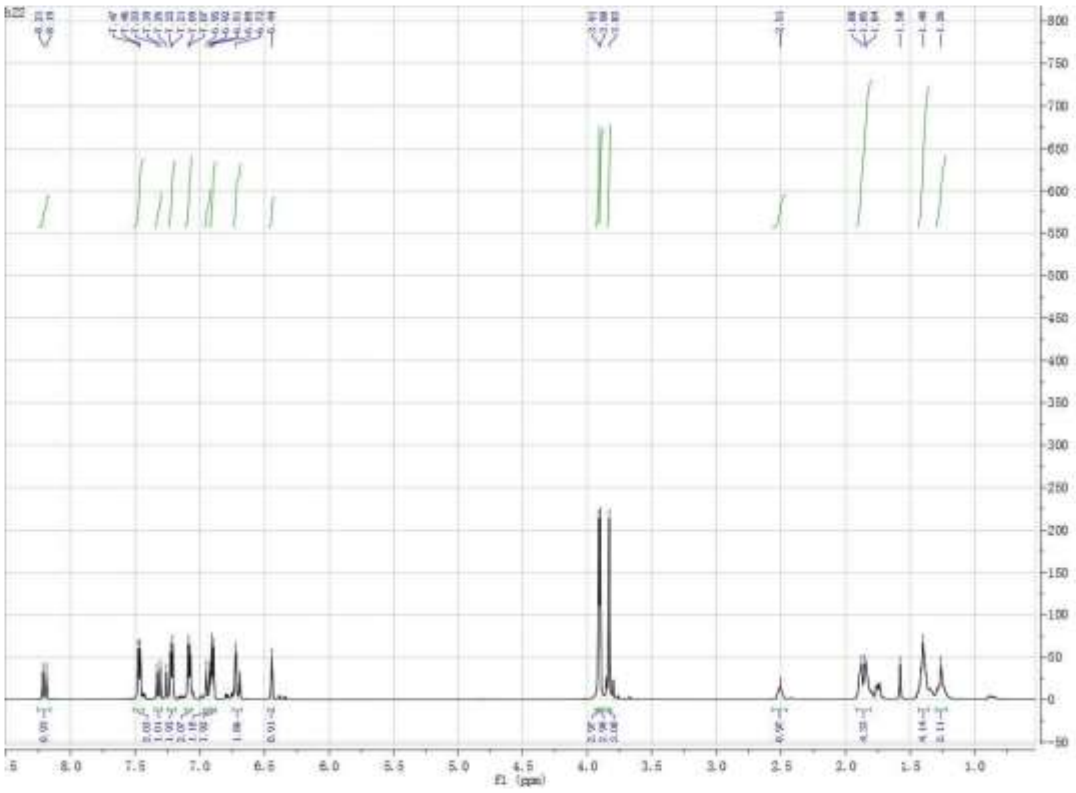

C13 NMR

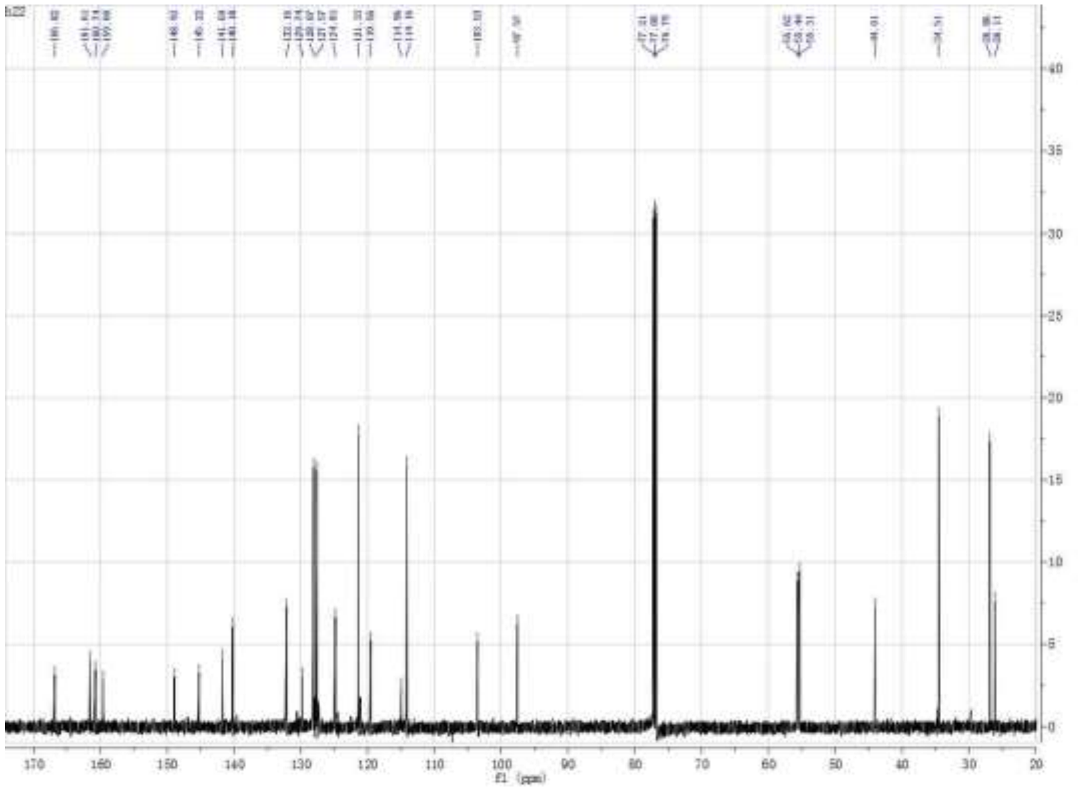

MS(EI):499.3($C_{32}H_{34}O_5$,$[M+H]^+$).

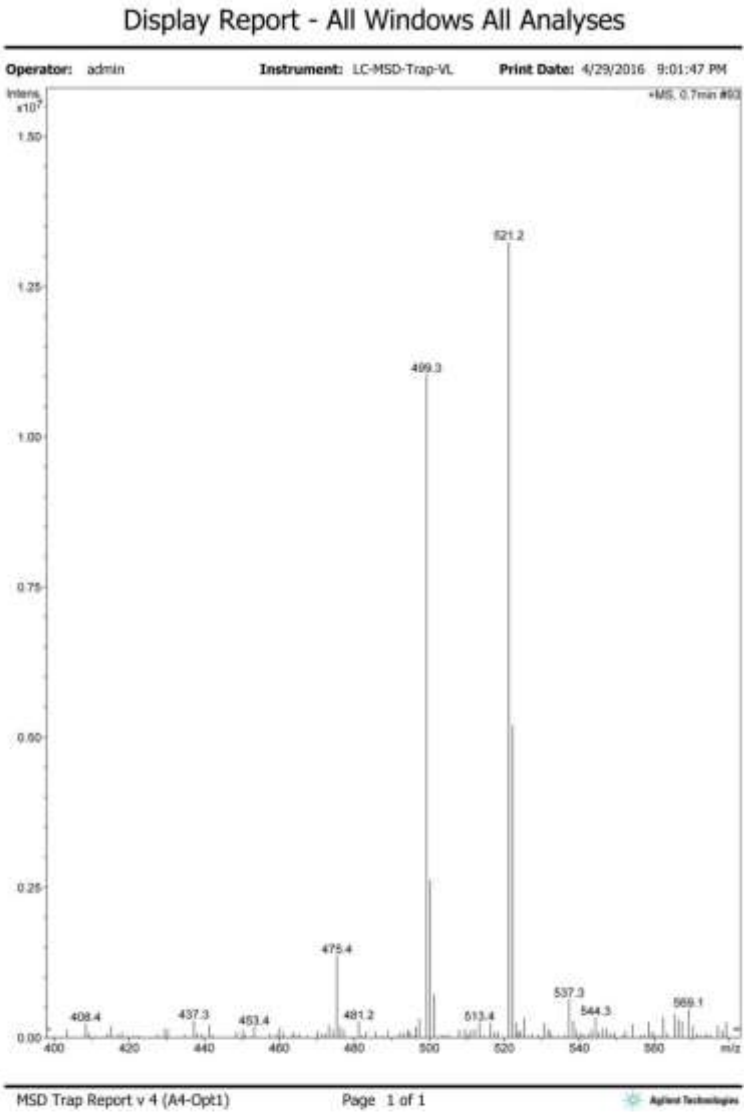
Display Report - All Windows All Analyses
Operator: admin
Instrument: LC-MSD-Trap-VL
Print Date: 4/29/2016 9:01:47 PM
Intens. x10^7
+MS, 0.7min #93
521.2
499.3
475.4
408.4
437.3
453.4
481.2
513.4
537.3
544.3
569.1
m/z
MSD Trap Report v 4 (A4-Opt1)
Page 1 of 1
Agilent Technologies

D17:

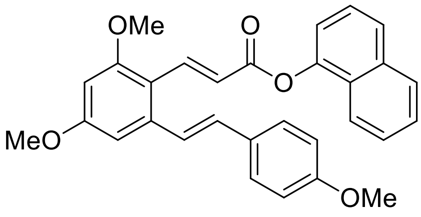
OMe
O
O
MeO
OMe

H1 NMR

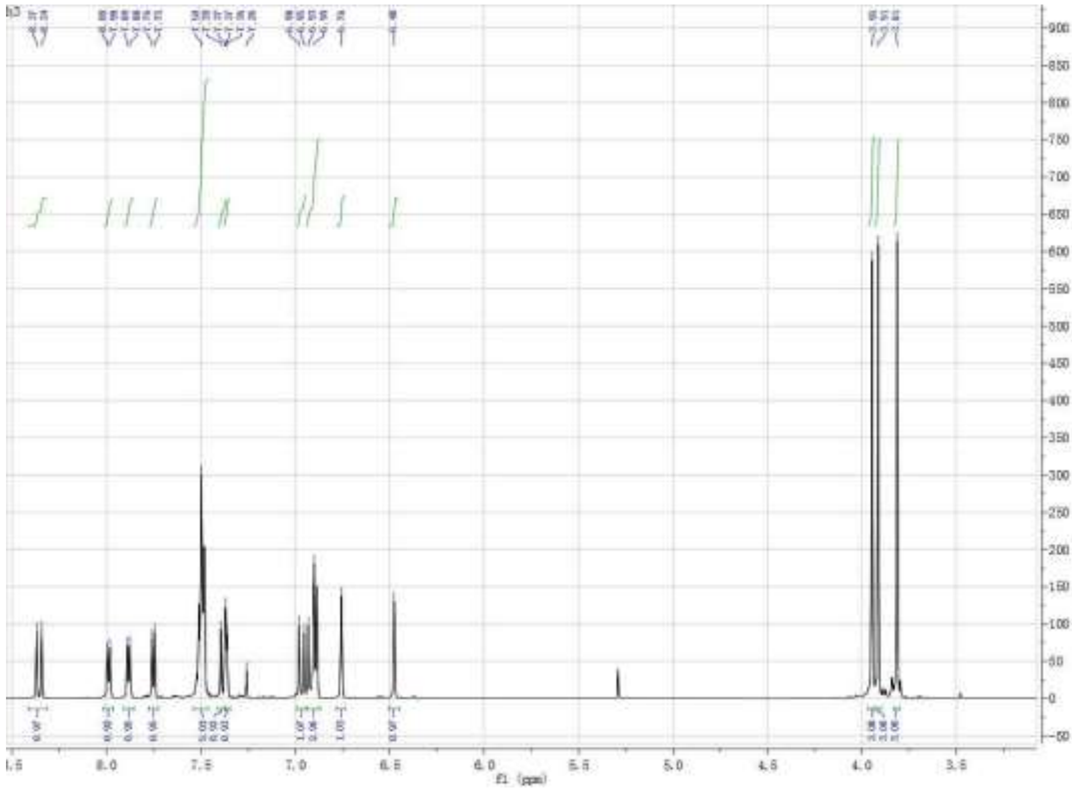

C13 NMR

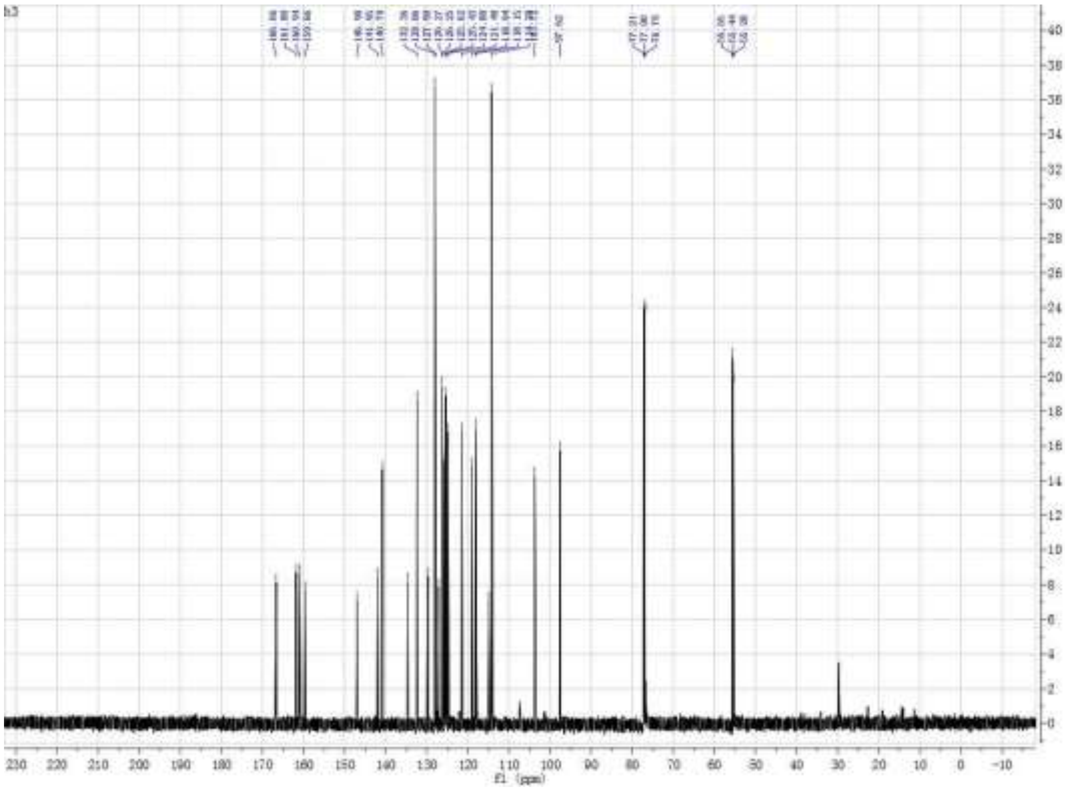

MS (EI): 467.2 ($C_{30}H_{26}O_5$, $[M+H]^+$).

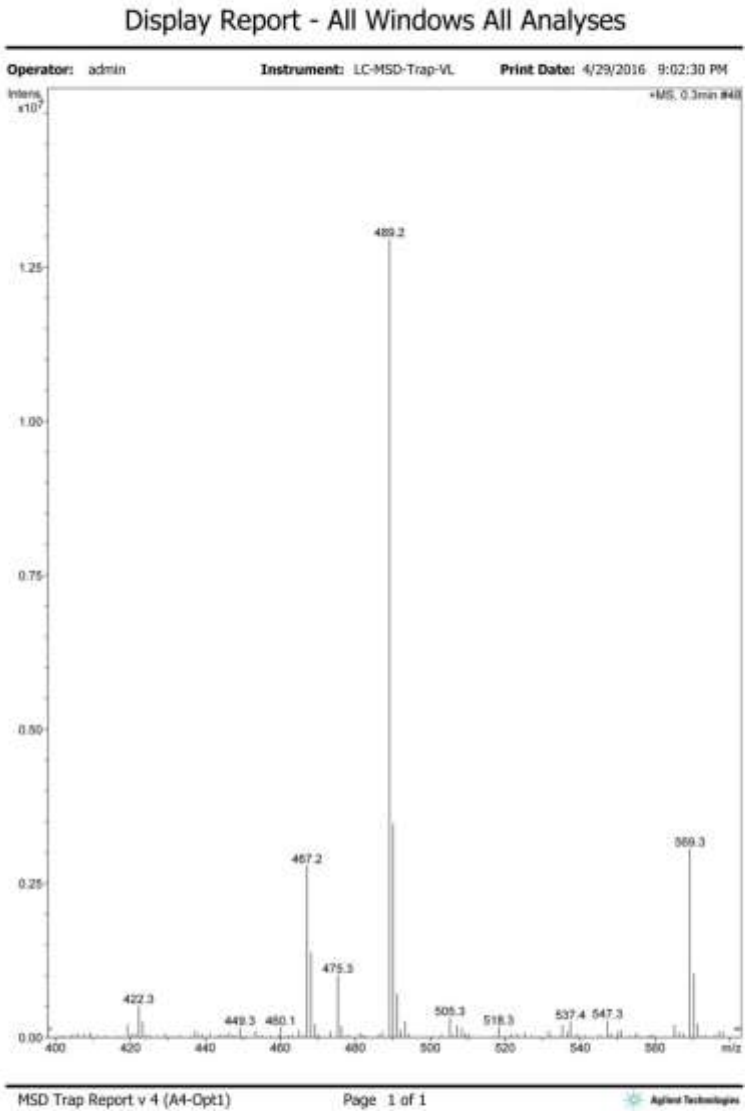
Display Report - All Windows All Analyses
Operator: admin
Instrument: LC-MSD-Trap-VL
Print Date: 4/29/2016 9:02:30 PM
Intens. x10^7
+MS, 0.3min #48
489.2
467.2
569.3
475.3
422.3
449.3
460.1
505.3
518.3
537.4
547.3
m/z
MSD Trap Report v 4 (A4-Opt1)
Page 1 of 1
Agilent Technologies

D18:

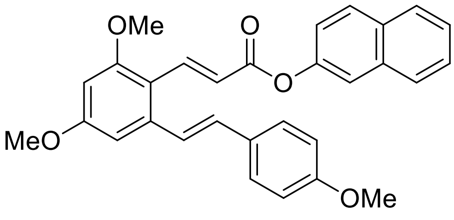
OMe
O
O
MeO
OMe

H^1 NMR

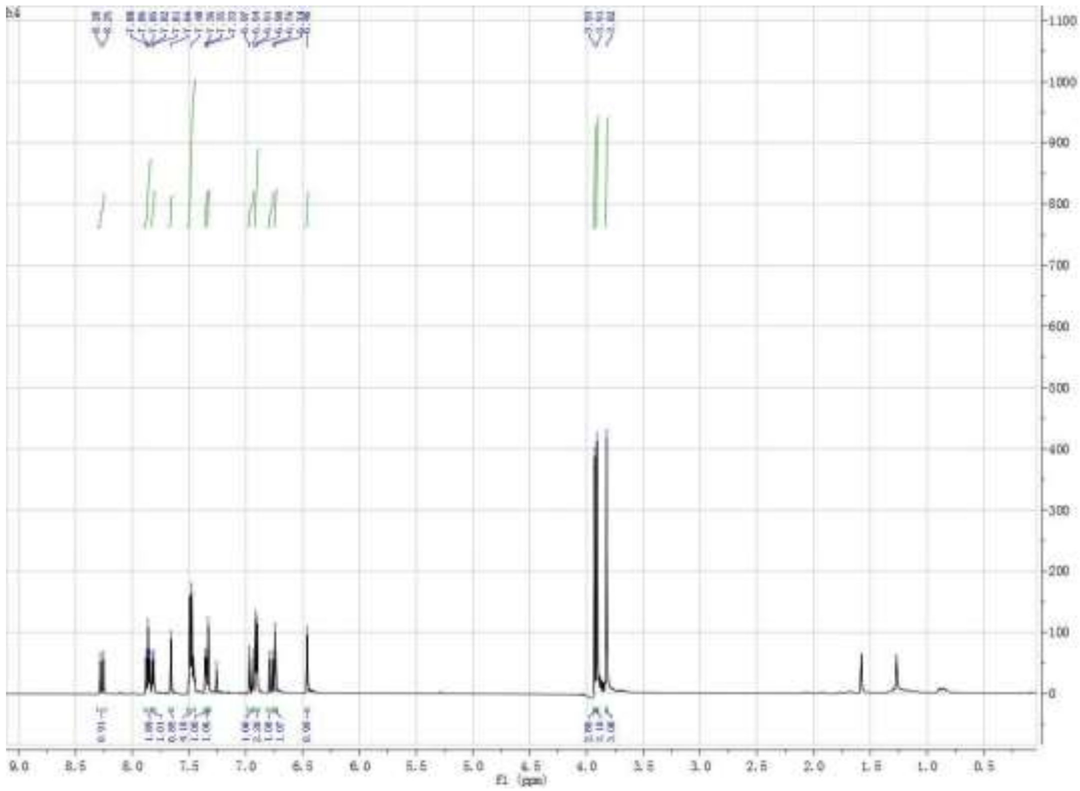

C^{13} NMR

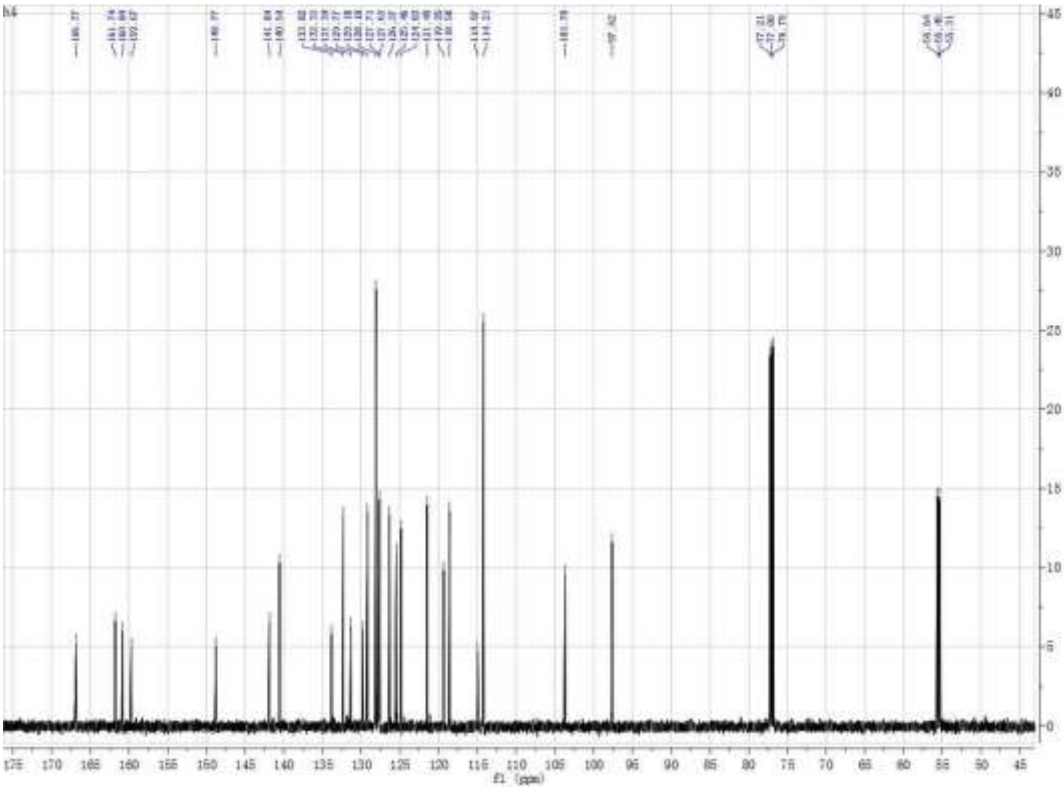

MS (EI): 467.2 ($C_{30}H_{26}O_5$, $[M+H]^+$).

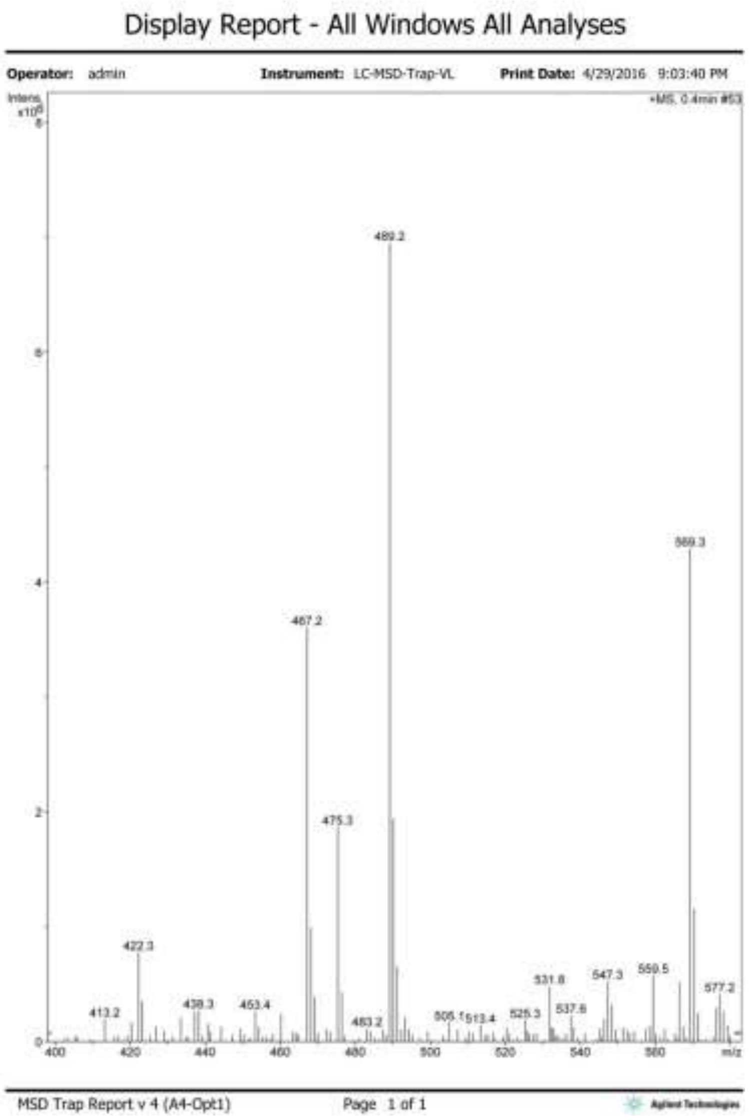
Display Report - All Windows All Analyses
Operator: admin
Instrument: LC-MSD-Trap-VL
Print Date: 4/29/2016 9:03:40 PM
+MS, 0.4min #53
489.2
569.3
467.2
475.3
422.3
413.2
438.3
453.4
483.2
505.1
513.4
525.3
531.8
537.6
547.3
559.5
577.2
m/z
MSD Trap Report v 4 (A4-Opt1)
Page 1 of 1

D19:

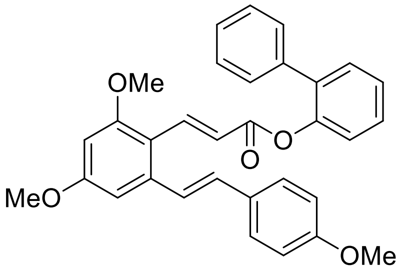
OMe
MeO
O
O
OMe

H1 NMR

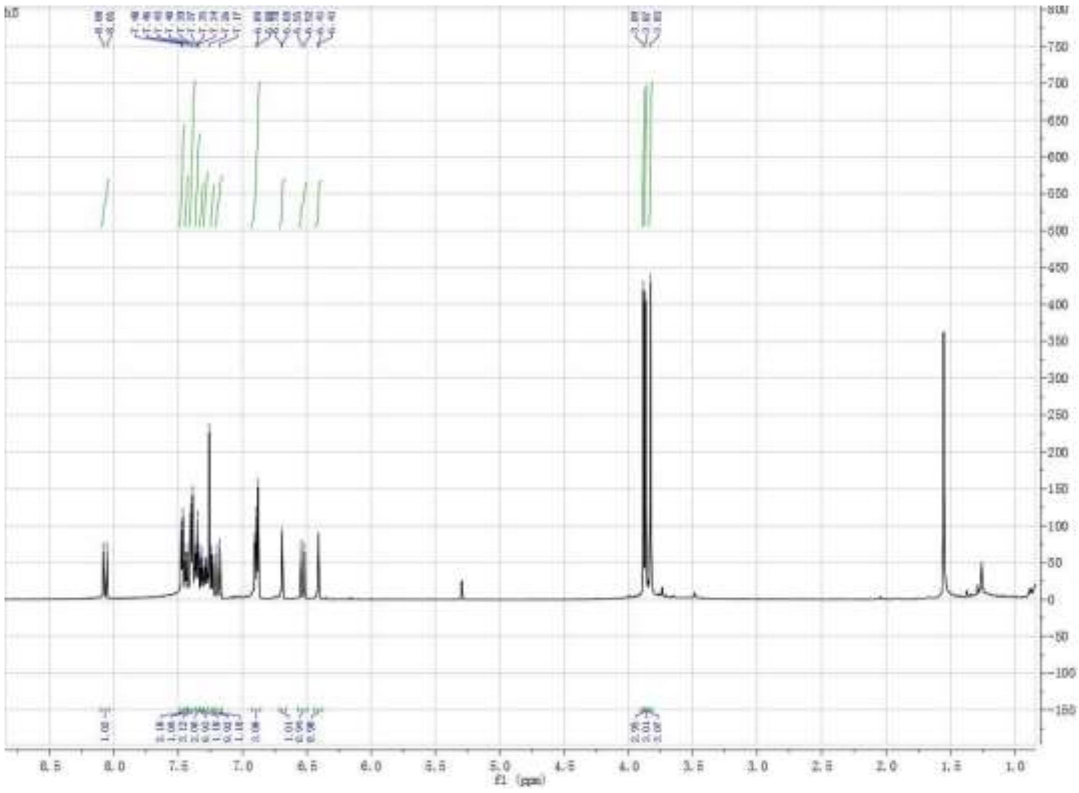

C^{13} NMR

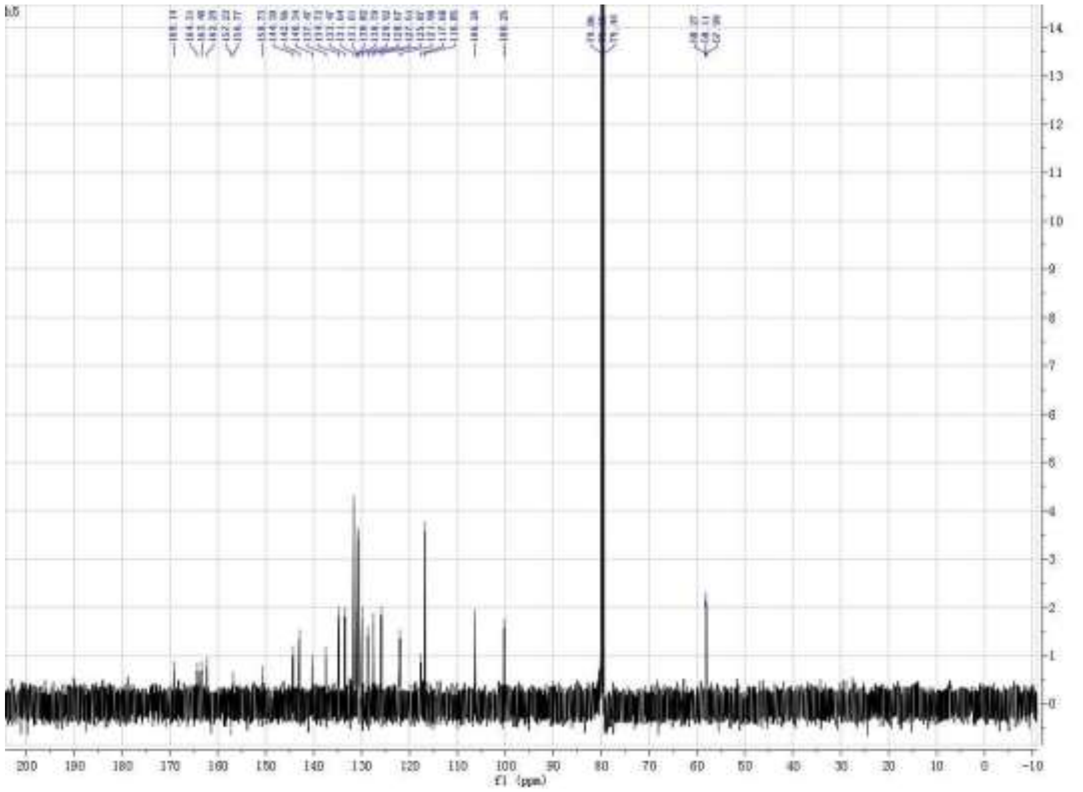

MS (EI): 493.2 ($C_{32}H_{28}O_5$, $[M+H]^+$).

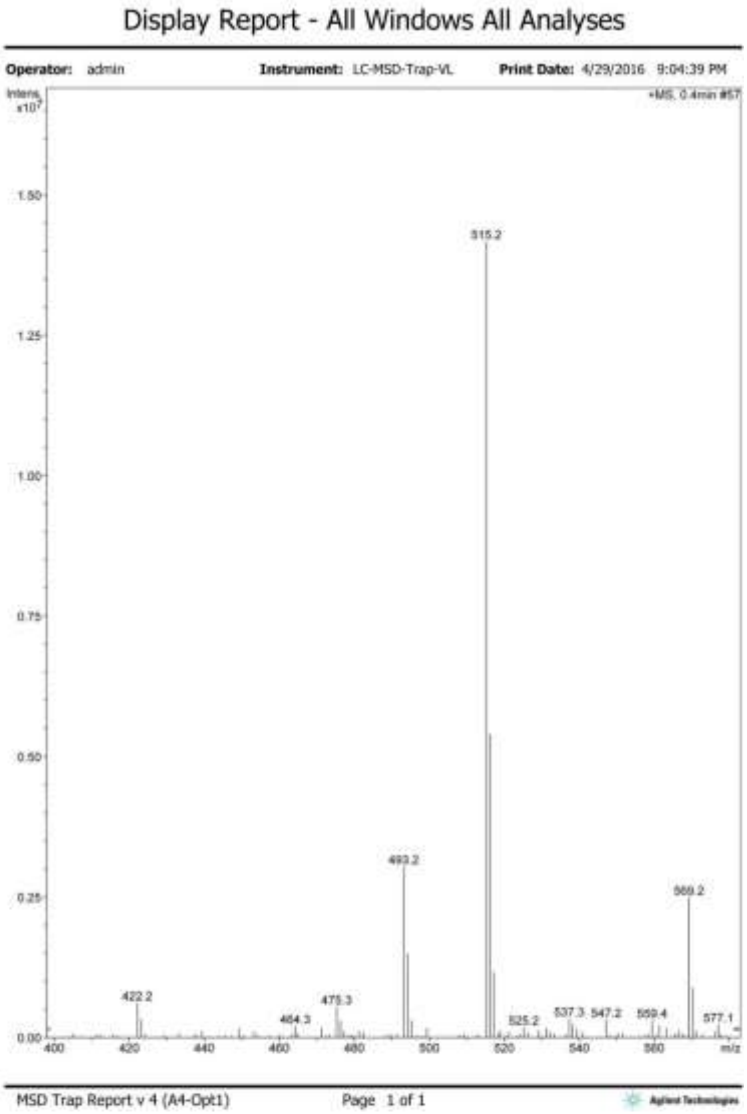
Display Report - All Windows All Analyses
Operator: admin
Instrument: LC-MSD-Trap-VL
Print Date: 4/29/2016 9:04:39 PM
+MS, 0.4min #57
Intens. x10^7
515.2
493.2
569.2
422.2
475.3
464.3
525.2
537.3
547.2
559.4
577.1
m/z
MSD Trap Report v 4 (A4-Opt1)
Page 1 of 1
Agilent Technologies

D20:

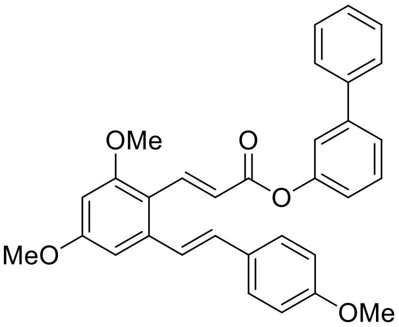
OMe
O
O
MeO
OMe

H^1 NMR

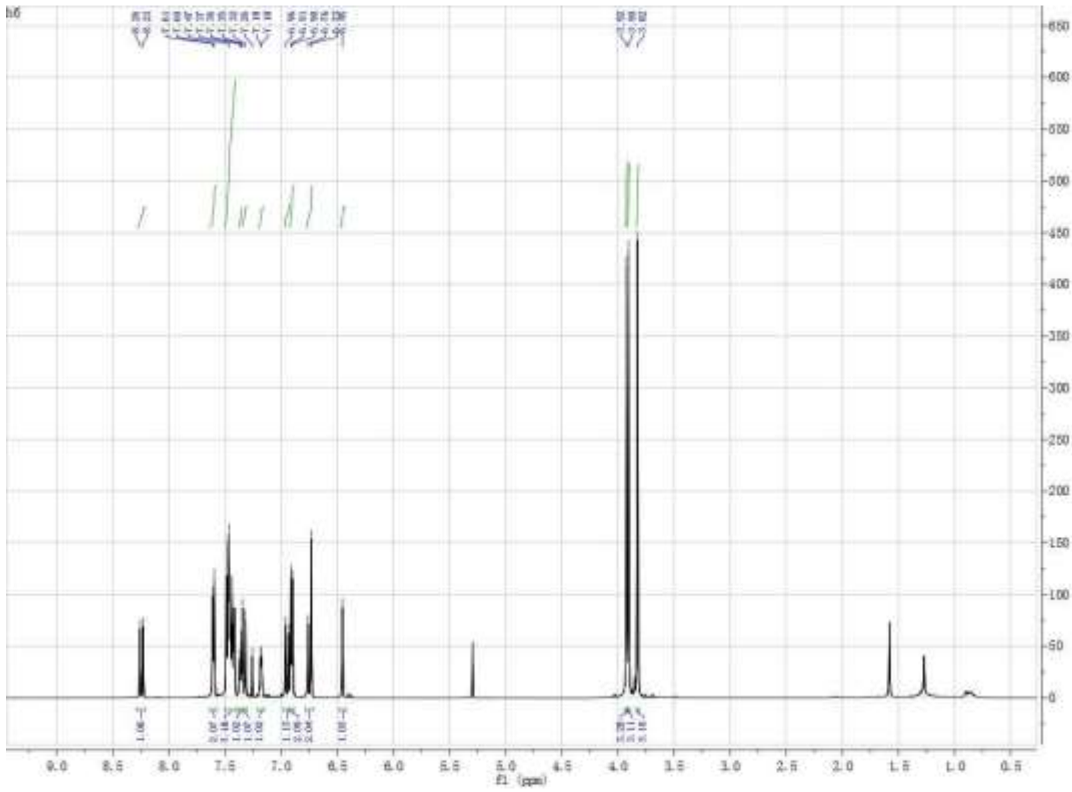

C^{13} NMR

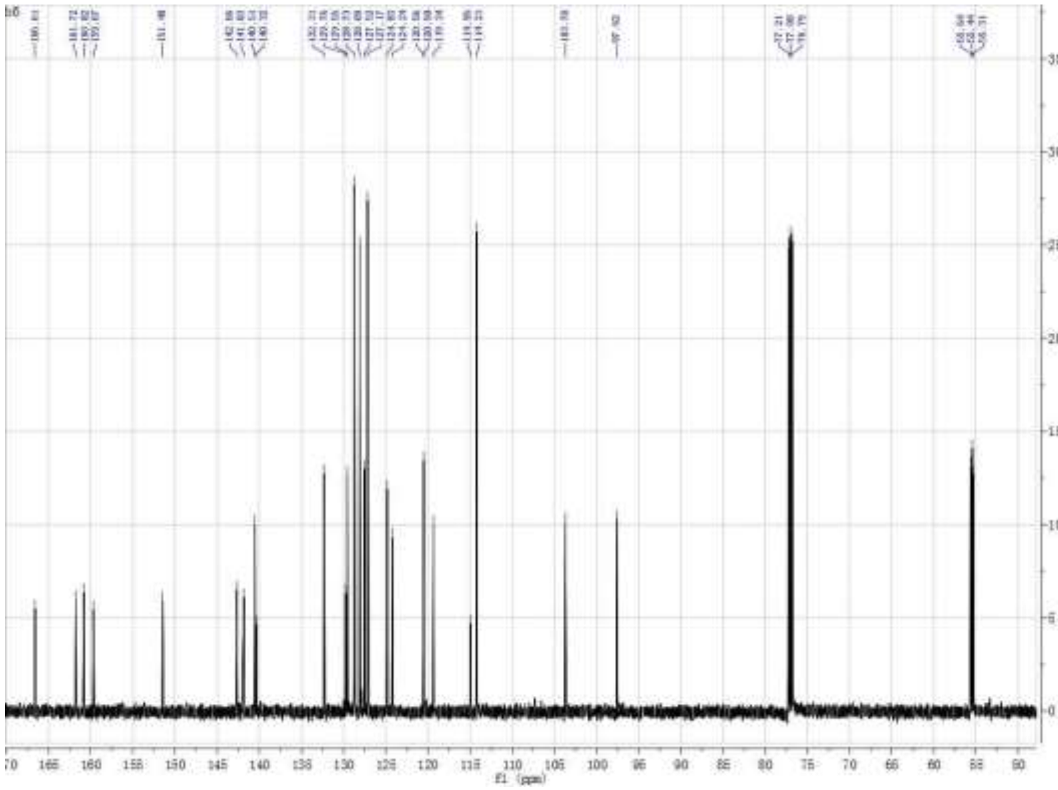

MS (EI): 493.2 ($C_{32}H_{28}O_5$, $[M+H]^+$).

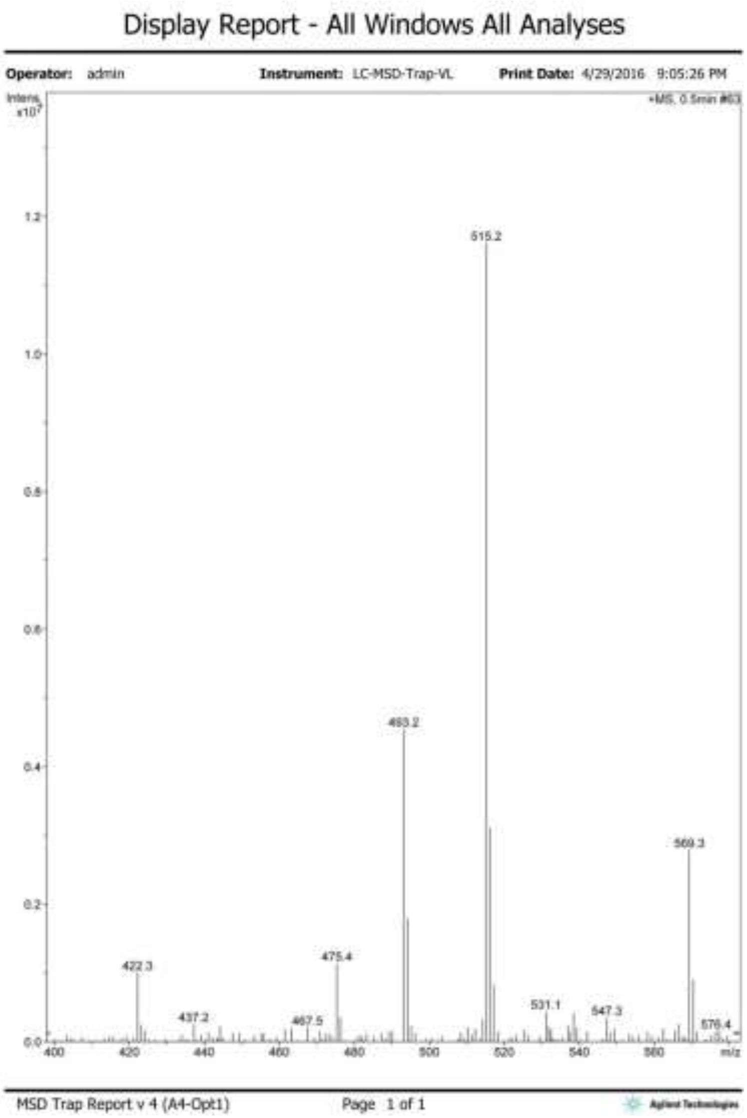

Display Report - All Windows All Analyses
Operator: admin
Instrument: LC-MSD-Trap-VL
Print Date: 4/29/2016 9:05:26 PM
Intens. x10^7
+MS, 0.5min #63
1.2
1.0
0.8
0.6
0.4
0.2
0.0
515.2
493.2
569.3
422.3
475.4
437.2
467.5
531.1
547.3
576.4
400
420
440
460
480
500
520
540
560
m/z
MSD Trap Report v 4 (A4-Opt1)
Page 1 of 1
Agilent Technologies

D21:

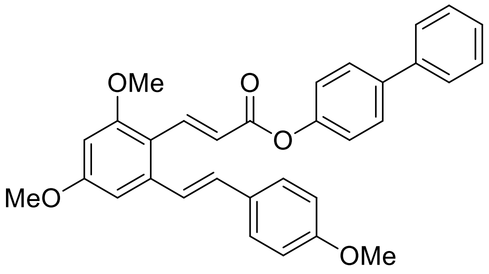

OMe
O
O
MeO
OMe

H^1 NMR

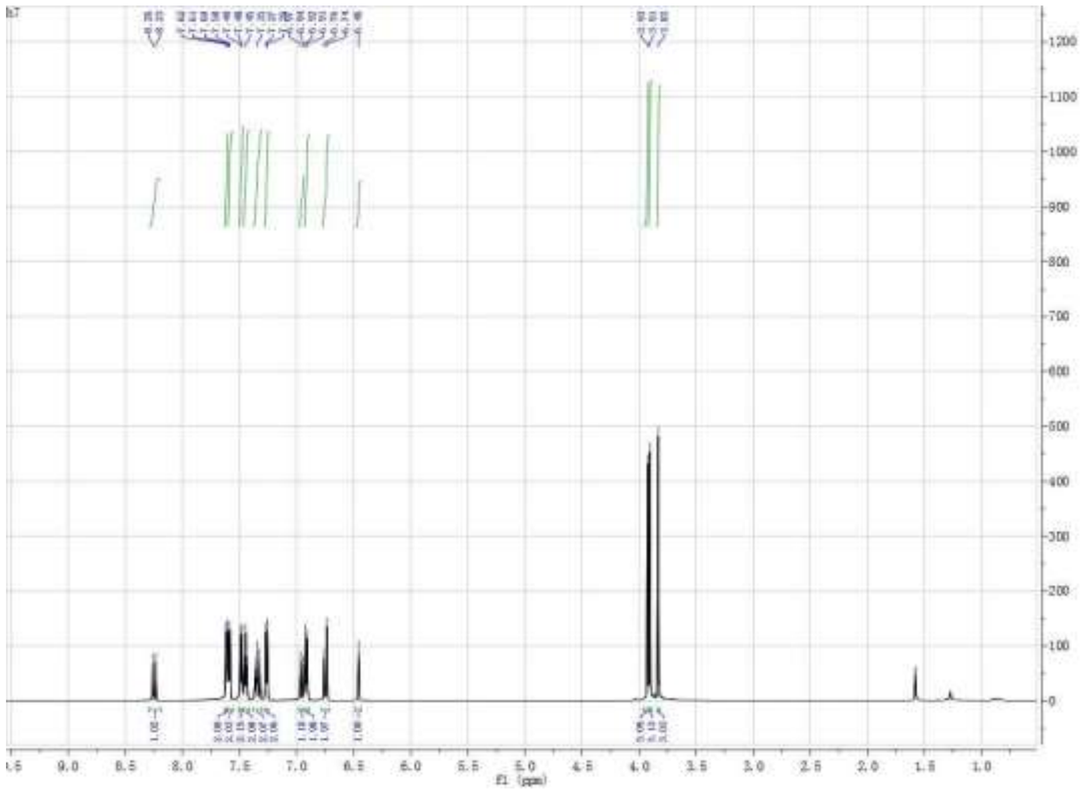

C^{13} NMR

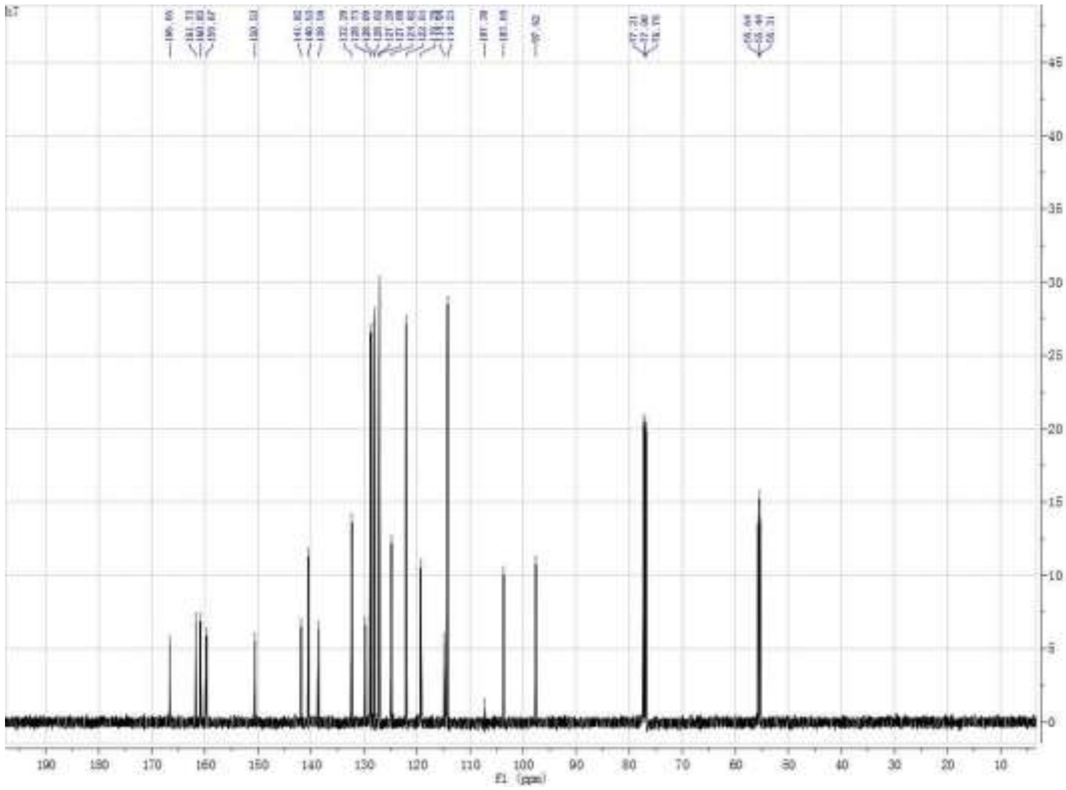

MS (EI): 493.2 ($C_{32}H_{28}O_5$, $[M+H]^+$).

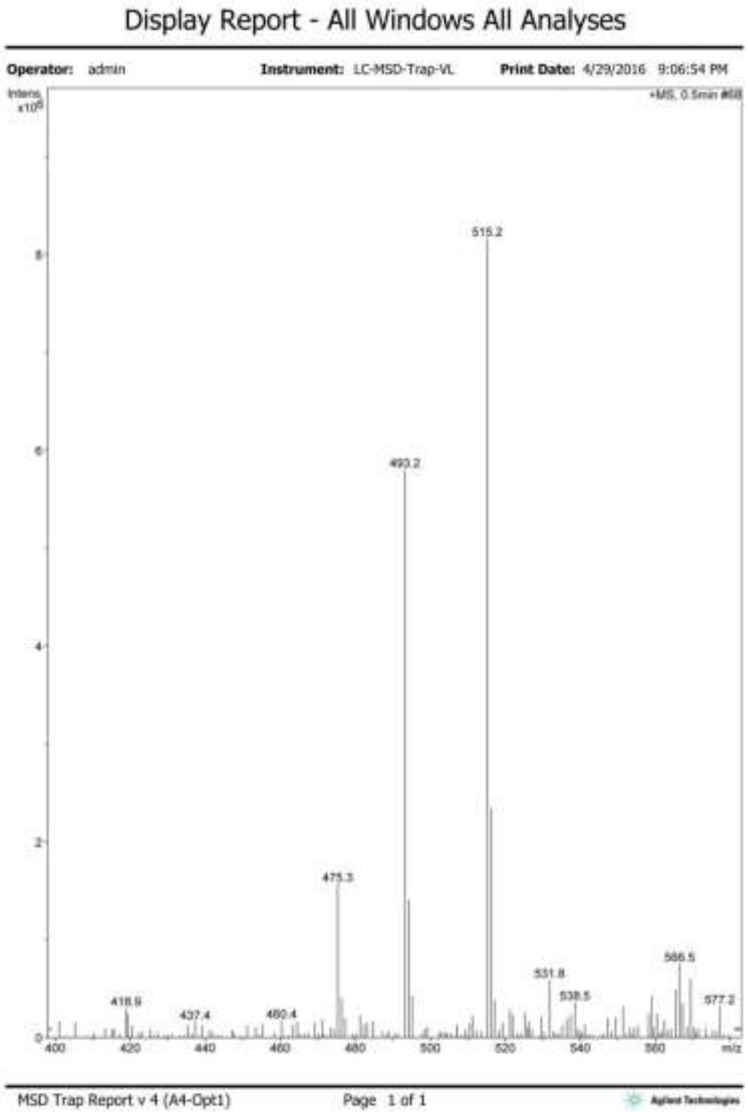
Display Report - All Windows All Analyses
Operator: admin
Instrument: LC-MSD-Trap-VL
Print Date: 4/29/2016 9:06:54 PM
Intens.
x10^6
515.2
493.2
475.3
418.9
437.4
460.4
531.8
538.5
566.5
577.2
m/z
MSD Trap Report v 4 (A4-Opt1)
Page 1 of 1

D22:

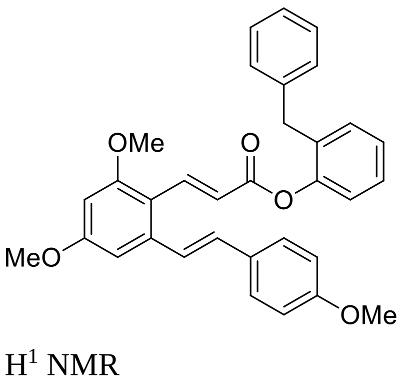
OMe
O
O
MeO
OMe

H^1 NMR

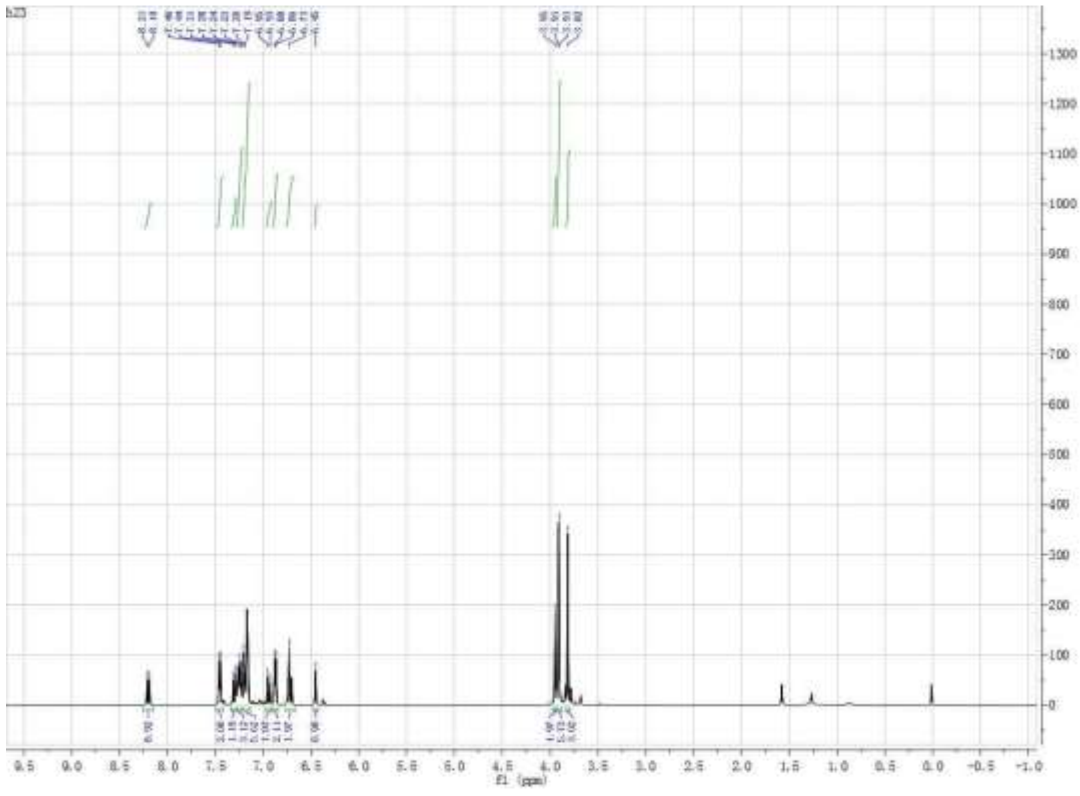

C13 NMR

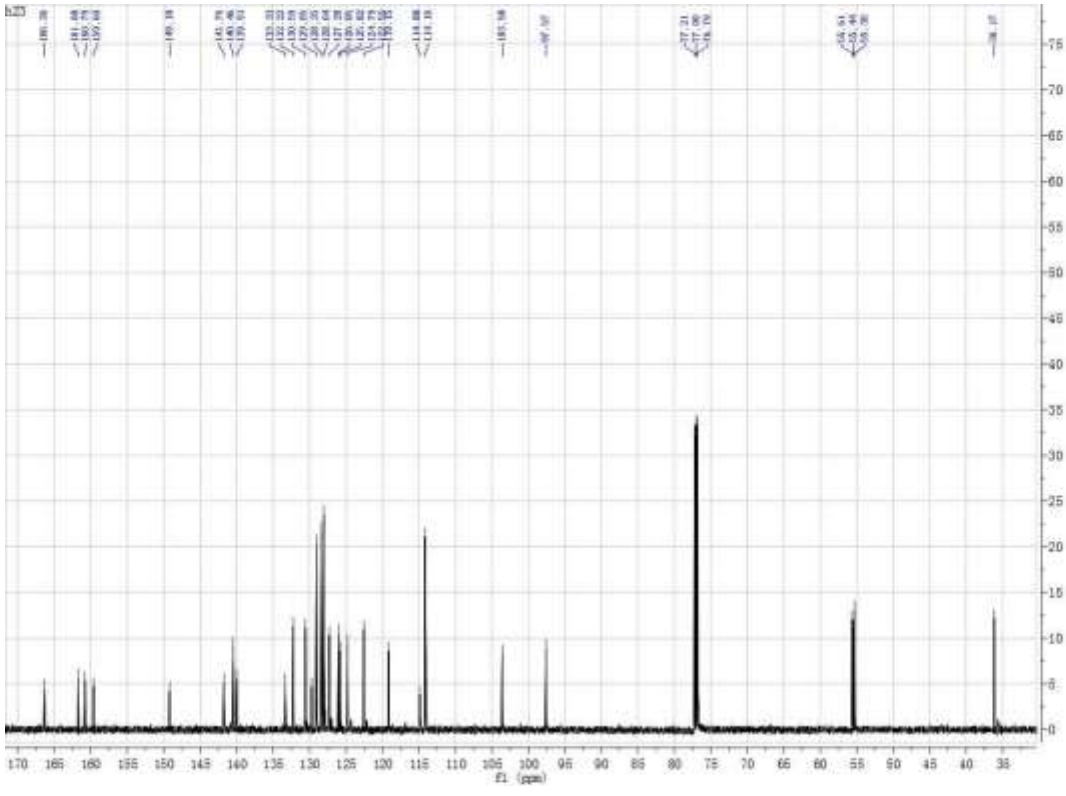

MS (EI): 507.2 ($C_{33}H_{30}O_5$, $[M+H]^+$).

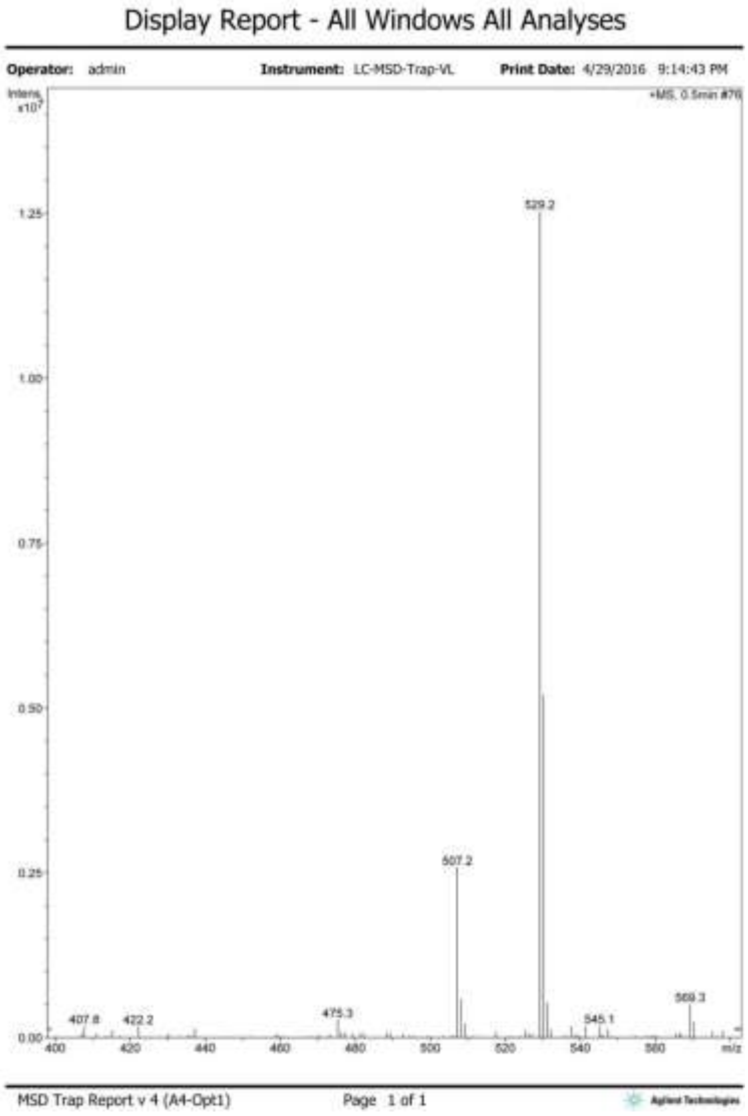
Display Report - All Windows All Analyses
Operator: admin
Instrument: LC-MSD-Trap-VL
Print Date: 4/29/2016 9:14:43 PM
Intens. x10^7
529.2
507.2
569.3
545.1
475.3
422.2
407.8
m/z
MSD Trap Report v 4 (A4-Opt1)
Page 1 of 1
Agilent Technologies

D23:

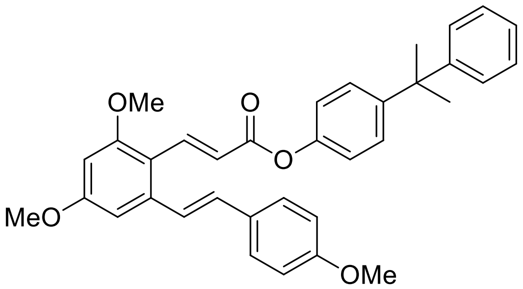
OMe
O
O
MeO
OMe

H[1] NMR

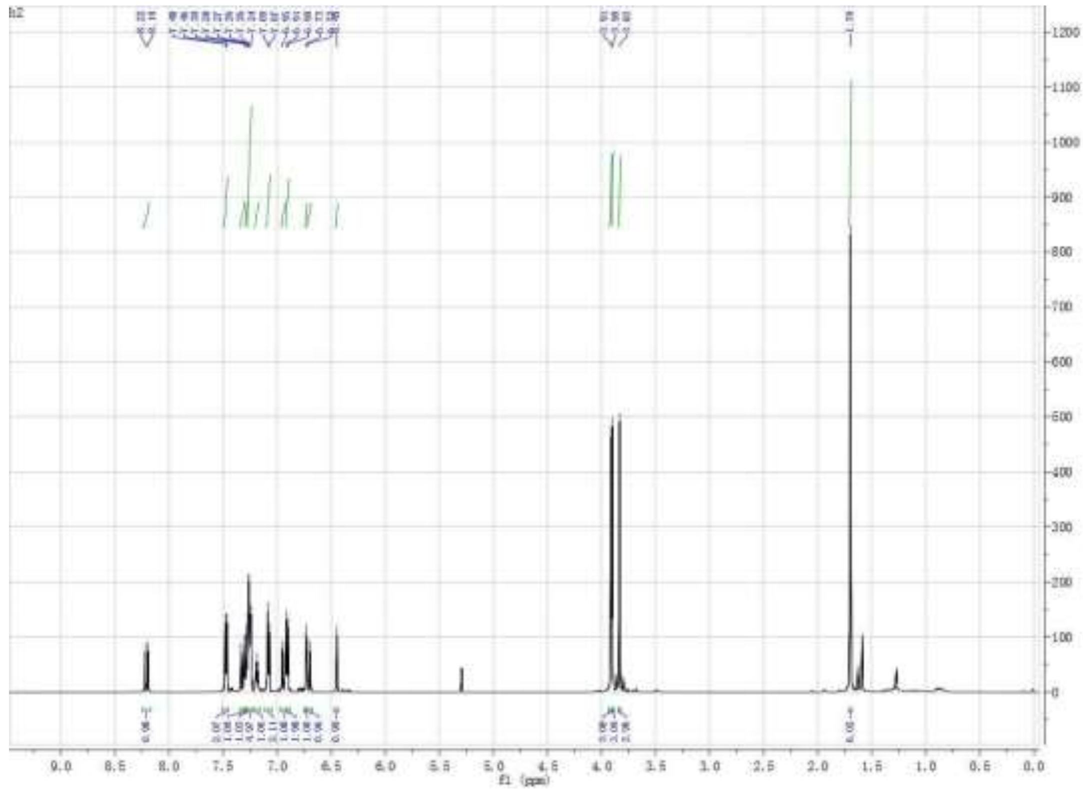

C13 NMR

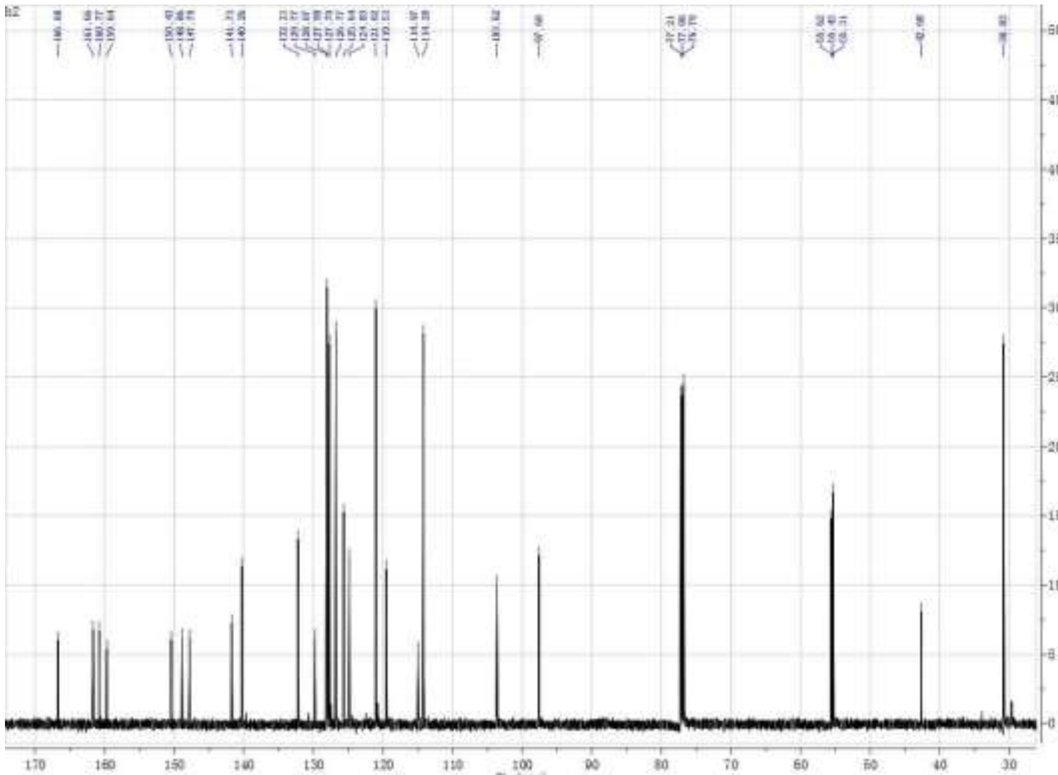

MS (EI): 535.3 ($C_{35}H_{34}O_5$, $[M+H]^+$).

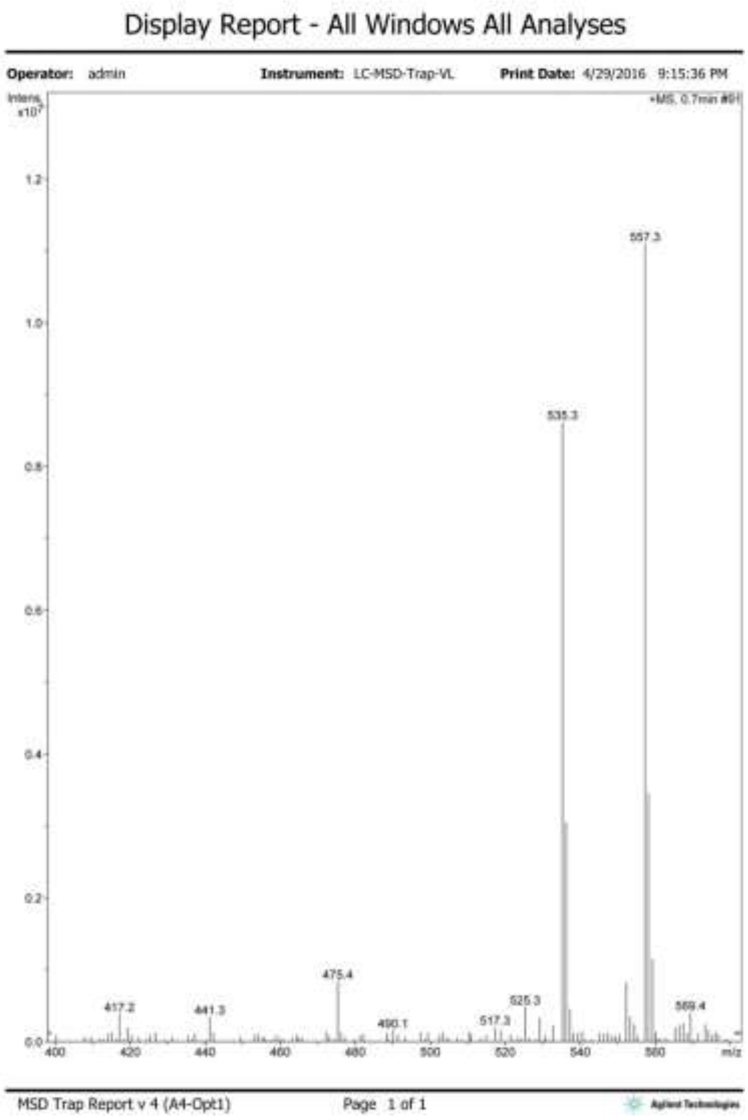
Display Report - All Windows All Analyses
Operator: admin
Instrument: LC-MSD-Trap-VL
Print Date: 4/29/2016 9:15:36 PM
Intens. x10^7
+MS, 0.7min #91
1.2
1.0
0.8
0.6
0.4
0.2
0.0
557.3
535.3
475.4
417.2
441.3
490.1
517.3
525.3
569.4
400
420
440
460
480
500
520
540
560
m/z
MSD Trap Report v 4 (A4-Opt1)
Page 1 of 1
Agilent Technologies
